# Comparison of Prolonged Exposure vs Cognitive Processing Therapy for Treatment of Posttraumatic Stress Disorder Among US Veterans: A Randomized Clinical Trial

**DOI:** 10.1001/jamanetworkopen.2021.36921

**Published:** 2022-01-19

**Authors:** Paula P. Schnurr, Kathleen M. Chard, Josef I. Ruzek, Bruce K. Chow, Patricia A. Resick, Edna B. Foa, Brian P. Marx, Matthew J. Friedman, Michelle J. Bovin, Kristina L. Caudle, Diane Castillo, Kyle T. Curry, Michael Hollifield, Grant D. Huang, Christine L. Chee, Millie C. Astin, Benjamin Dickstein, Kerry Renner, Carolina P. Clancy, Claire Collie, Kelly Maieritsch, Su Bailey, Karin Thompson, Michael Messina, Laurel Franklin, Steve Lindley, Karen Kattar, Brandi Luedtke, Jennifer Romesser, John McQuaid, Patrick Sylvers, Ruth Varkovitzky, Lori Davis, David MacVicar, Mei-Chiung Shih

**Affiliations:** 1 Executive Division, National Center for PTSD, White River Junction, Vermont; 2 Geisel School of Medicine at Dartmouth, Hanover, New Hampshire; 3 Cincinnati VA Medical Center, Cincinnati, Ohio; 4 University of Cincinnati, Cincinnati, Ohio; 5 Department of Psychiatry and Behavioral Sciences, Stanford University, Stanford, California; 6 Palo Alto University, Palo Alto, California; 7 Department of Psychology, University of Colorado, Colorado Springs; 8 VA Cooperative Studies Program Coordinating Center, Palo Alto, California; 9 Duke Health, Durham, North Carolina; 10 University of Pennsylvania Perelman School of Medicine, Department of Psychiatry, Philadelphia; 11 Behavioral Science Division, National Center for PTSD, Boston, Massachusetts; 12 VA Boston Healthcare System, Boston, Massachusetts; 13 Boston University School of Medicine, Boston, Massachusetts; 14 Center of Excellence, Central Texas VA Health Care System, Waco; 15 Minneapolis VA Medical Center, Minneapolis, Minnesota; 16 Tibor Rubin VA Medical Center, Long Beach, California; 17 The George Washington University School of Medicine and Health Sciences, Washington, District of Columbia; 18 Department of Psychiatry and Human Behavior, University of California, Riverside; 19 Cooperative Studies Program Central Office, Department of Veterans Affairs Office of Research & Development, Washington, District of Columbia; 20 Raymond G. Murphy VA Medical Center, Albuquerque, New Mexico; 21 Atlanta VA Medical Center, Atlanta, Georgia; 22 VA Northeast Ohio Healthcare System, Cleveland; 23 Durham VA Medical Center, Durham, North Carolina; 24 Edward Hines Jr. VA Hospital, Hines, Illinois; 25 Michael E. DeBakey VA Medical Center, Houston, Texas; 26 Menninger Department of Psychiatry and Behavioral Sciences, Baylor College of Medicine, Houston, Texas; 27 William S. Middleton Memorial Veterans Hospital, Madison, Wisconsin; 28 Department of Psychiatry, University of Wisconsin–Madison School of Medicine and Public Health, Madison; 29 New Orleans VA Medical Center, New Orleans, Louisiana; 30 South Central VA Mental Illness Research, Education and Clinical Center, New Orleans, Louisiana; 31 Palo Alto VA Medical Center, Palo Alto, California; 32 Department of Psychiatry and Behavioral Sciences, Stanford School of Medicine, Stanford University; 33 Phoenix VA Medical Center, Phoenix, Arizona; 34 George E. Whalen VA Medical Center, Salt Lake City, Utah; 35 San Francisco VA Medical Center, San Francisco, California; 36 Department of Psychiatry and Behavioral Sciences, Weill Institute of Neuroscience, University of California, San Francisco; 37 VA Puget Sound Health Care System, American Lake Division, Tacoma, Washington; 38 Department of Psychiatry and Behavioral Sciences, University of Washington, Seattle; 39 Tuscaloosa VA Medical Center, Tuscaloosa, Alabama; 40 Department of Psychiatry, University of Alabama Heersink School of Medicine, Birmingham

## Abstract

**Importance:**

Posttraumatic stress disorder (PTSD) is a prevalent and serious mental health problem. Although there are effective psychotherapies for PTSD, there is little information about their comparative effectiveness.

**Objective:**

To compare the effectiveness of prolonged exposure (PE) vs cognitive processing therapy (CPT) for treating PTSD in veterans.

**Design, Setting, and Participants:**

This randomized clinical trial assessed the comparative effectiveness of PE vs CPT among veterans with military-related PTSD recruited from outpatient mental health clinics at 17 Department of Veterans Affairs medical centers across the US from October 31, 2014, to February 1, 2018, with follow-up through February 1, 2019. The primary outcome was assessed using centralized masking. Tested hypotheses were prespecified before trial initiation. Data were analyzed from October 5, 2020, to May 5, 2021.

**Interventions:**

Participants were randomized to 1 of 2 individual cognitive-behavioral therapies, PE or CPT, delivered according to a flexible protocol of 10 to 14 sessions.

**Main Outcomes and Measures:**

The primary outcome was change in PTSD symptom severity on the Clinician-Administered PTSD Scale for *DSM-5* (CAPS-5) from before treatment to the mean after treatment across posttreatment and 3- and 6-month follow-ups. Secondary outcomes included other symptoms, functioning, and quality of life.

**Results:**

Analyses were based on all 916 randomized participants (730 [79.7%] men and 186 [20.3%] women; mean [range] age 45.2 [21-80] years), with 455 participants randomized to PE (mean CAPS-5 score at baseline, 39.9 [95% CI, 39.1-40.7] points) and 461 participants randomized to CPT (mean CAPS-5 score at baseline, 40.3 [95% CI, 39.5-41.1] points). PTSD severity on the CAPS-5 improved substantially in both PE (standardized mean difference [SMD], 0.99 [95% CI, 0.89-1.08]) and CPT (SMD, 0.71 [95% CI, 0.61-0.80]) groups from before to after treatment. Mean improvement was greater in PE than CPT (least square mean, 2.42 [95% CI, 0.53-4.31]; *P* = .01), but the difference was not clinically significant (SMD, 0.17). Results for self-reported PTSD symptoms were comparable with CAPS-5 findings. The PE group had higher odds of response (odds ratio [OR], 1.32 [95% CI, 1.00-1.65]; *P* < .001), loss of diagnosis (OR, 1.43 [95% CI, 1.12-1.74]; *P* < .001), and remission (OR, 1.62 [95% CI, 1.24-2.00]; *P* < .001) compared with the CPT group. Groups did not differ on other outcomes. Treatment dropout was higher in PE (254 participants [55.8%]) than in CPT (215 participants [46.6%]; *P* < .01). Three participants in the PE group and 1 participant in the CPT group were withdrawn from treatment, and 3 participants in each treatment dropped out owing to serious adverse events.

**Conclusions and Relevance:**

This randomized clinical trial found that although PE was statistically more effective than CPT, the difference was not clinically significant, and improvements in PTSD were meaningful in both treatment groups. These findings highlight the importance of shared decision-making to help patients understand the evidence and select their preferred treatment.

**Trial Registration:**

ClinicalTrials.gov Identifier: NCT01928732

## Key Points

**Question:**

How do prolonged exposure and cognitive processing therapy compare for the treatment of posttraumatic stress disorder (PTSD)?

**Findings:**

In this randomized clinical trial among 916 veterans of 2 evidence-based psychotherapies for PTSD, PTSD symptoms improved in both treatment groups. Prolonged exposure was more effective than cognitive processing therapy for reducing PTSD symptoms, but the difference between treatments did not reach the predetermined threshold for clinical significance.

**Meaning:**

These findings suggest that although prolonged exposure had an advantage over cognitive processing therapy for PTSD symptoms, patient preferences should be considered because both treatments resulted in meaningful improvements and did not differ in their effects on other outcomes.

## Teaser

This randomized clinical trial compares the effectiveness of prolonged exposure vs cognitive processing therapy for treating posttraumatic stress disorder in veterans.

## Introduction

In 2007, the US Department of Veterans Affairs (VA) began a national training program in evidence-based psychotherapy for VA clinicians that includes 2 cognitive-behavioral therapies for posttraumatic stress disorder (PTSD): cognitive processing therapy (CPT) and prolonged exposure (PE).^1^ Both treatments are recommended as first-line treatments in all PTSD practice guidelines,^2^ including the guideline issued by the VA and the Department of Defense.^3^ PTSD occurs after traumatic events, such as combat, assault, accidents, and disasters.^4^ Lifetime prevalence of PTSD in US adults is 6.1%.^5^ Among veterans who received VA health care in 2019, 12.1% had PTSD, including 26.5% of veterans who served in Iraq or Afghanistan.^6^

Despite the strong recommendations for trauma-focused psychotherapies like PE and CPT,^2,3^ their comparative effectiveness is largely unknown. A 2018 meta-analysis^7^ found standardized mean differences (SMDs) of 1.23 for exposure therapy (including PE) and 1.35 for CPT. In the only trial to compare CPT with PE to our knowledge, a 2002 study by Resick et al,^8^ treatments did not differ on PTSD or depression outcomes, although CPT produced greater reductions in some domains of guilt. Consequently, patients and clinicians must consider treatment options for PTSD without knowing how these options compare. Information about the comparative effectiveness of PTSD treatments can help patients make an informed choice^9^ and guide decision-making about which treatments to prioritize in health care systems, such as the VA.

The Agency for Healthcare Research and Quality has called for studies that compare psychological treatments for PTSD with the best evidence of efficacy.^7^ Therefore, we conducted a multisite randomized clinical trial comparing PE and CPT among veterans with PTSD. To our knowledge, no study has compared these treatments directly in veterans, who can be challenging to treat successfully.^10,11^ The study was a practical trial, conducted in multiple VA clinics, using broad inclusion and exclusion criteria, and with treatment flexibly delivered by many VA clinicians. The primary outcome was PTSD symptom severity. Hypothesis testing was nondirectional because there was no basis for predicting that one treatment would be more effective.

## Methods

This randomized clinical trial was approved by the VA’s Central Institutional Review Board. All participants gave written informed consent before participation. This study is reported following the Consolidated Standards of Reporting Trials (CONSORT) reporting guideline.

Study methods have been published previously^12^ and are available in the trial protocol in Supplement 1. The study was a parallel 2-arm randomized clinical trial in which participants at 17 VA medical centers were randomized to receive either PE or CPT using a 1:1 allocation ratio within each site in permuted blocks. A VA Cooperative Studies Program centralized coordinating center conducted computer-generated randomization and transmitted information to the Coordinator at each site after participant eligibility was confirmed.

### Participants

Participants were veterans with military-related PTSD (Figure). Inclusion criteria were current PTSD according to *Diagnostic and Statistical Manual of Mental Disorders* (Fifth Edition) (*DSM-5*)^4^ and severity of 25 points or greater on the Clinician-Administered PTSD Scale for *DSM-5*^13^ (CAPS-5), agreement to not receive nonstudy PTSD psychotherapy during treatment and allow recording of interviews and therapy, and access to a telephone for remotely-conducted diagnostic assessments (or agreement to come to the VA). Medications for PTSD and other mental or physical conditions, psychotherapy for other problems, brief visits with an existing therapist, and self-help groups were allowed. Individuals using medication were initially required to have no changes in drugs or dosage for 2 months before entry; after consultation with the study data safety and monitoring board, the duration was reduced to 1 month to enhance recruitment. Exclusion criteria were substance dependence not in remission for 1 month (not having or needing detoxification), current psychotic symptoms or mania, current suicidal or homicidal intent requiring immediate attention, or moderate or severe cognitive impairment.

**Figure.**
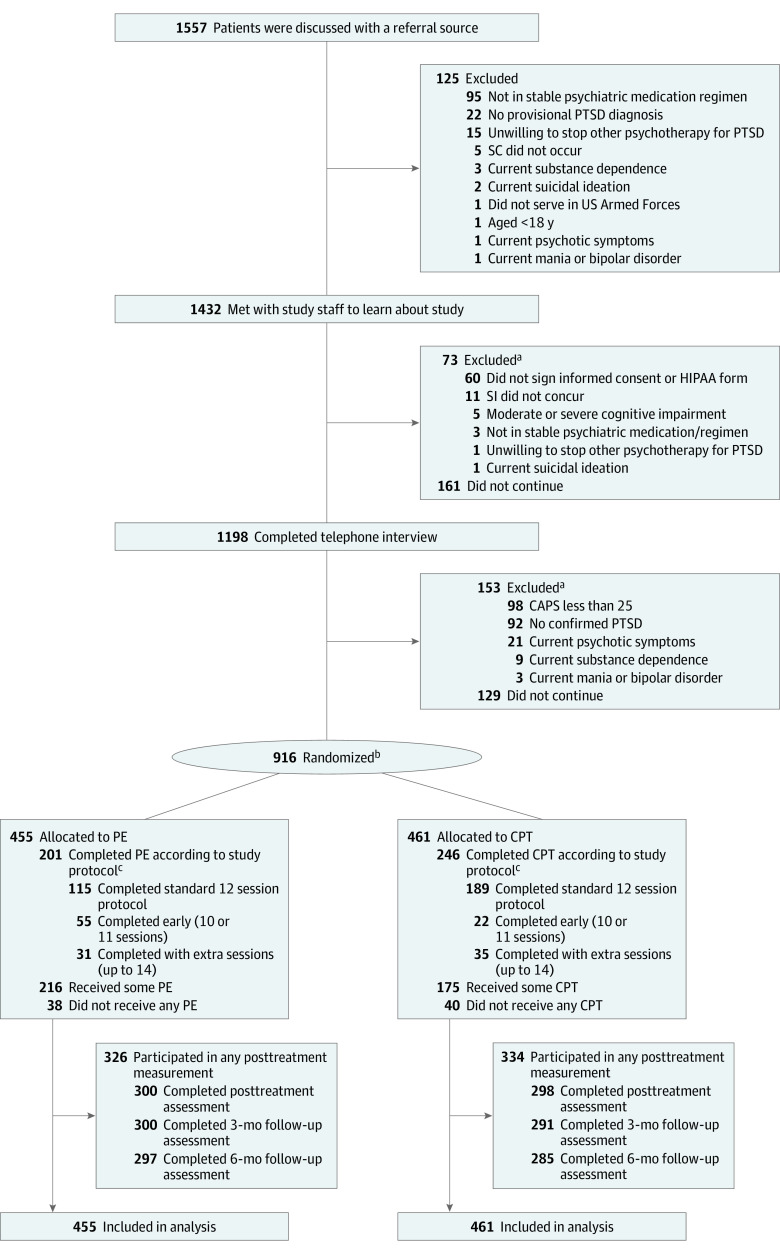
Participant Recruitment Flowchart ^a^Participants may have multiple reasons for exclusion. ^b^One ineligible patient was randomized and met the Clinician-Administered PTSD Scale for DSM-5 (CAPS-5) cutoff score but did not meet on Criterion E. One patient who was randomized to cognitive processing therapy (CPT) inadvertently received prolong exposure (PE), and 1 patient who was randomized to PE received CPT owing to staff error. ^c^Completion according to study protocol could be between 10 and 14 sessions; the number of sessions could be flexed according to patient need.

### Measures

The primary outcome, as specified in the trial protocol, was change in PTSD symptom severity on the CAPS-5, a clinician-administered structured interview,^13^ from before treatment to the mean after treatment across posttreatment and 3- and 6-month follow-ups. The 20 PTSD symptoms on the CAPS-5 are rated on a 0 to 4 scale and are summed for total severity (range, 0-80). Symptoms are counted present if they are rated at 2 or greater. We used the CAPS-5 to compute additional outcomes^14^: response (≥10-point improvement in severity), loss of diagnosis (response plus no longer meeting *DSM-5* symptom criteria and severity score <25 points), and remission (loss of diagnosis plus severity score <12 points). Categorizations had previously been validated using measures of functioning and quality of life.^14^

Prior to each session, participants completed the PTSD Checklist for *DSM-5* (PCL-5)^15^ for PTSD and 9-item Patient Health Questionnaire (PHQ-9)^16^ for depression, per therapy protocols, and not for outcome assessment. The PTSD Diagnostic Scale for *DSM-5* (PDS-5)^17^ and Beck Depression Inventory II (BDI-II)^18^ scores were independent outcomes. Additional secondary outcomes were anger,^19^ substance use,^20,21^ functioning,^22^ quality of life,^23^ and satisfaction.^24^ All secondary outcomes are reported except service utilization, which will be reported separately. Owing to administrative error, secondary outcomes were not preregistered. Measures to establish eligibility and for sample description included the Research Version of the Structured Clinical Interview for *DSM-5* (SCID-5-RV),^25^ Montreal Cognitive Assessment,^26^ outcome expectancy,^27^ and questions about demographic characteristics.^12^ Race and ethnicity were self-reported and were included for sample description.

We randomly selected 200 CAPS-5 assessments and 100 SCID assessments to be rated by independent doctoral-level assessors to assess interrater reliability. The intraclass correlation was 0.97 for total severity on the CAPS-5. Median (range) κ was 0.91 (0.80-0.98) for current SCID-5 diagnosis and 0.98 (0.66-1.00) for past SCID-5 diagnoses.

### Recruitment

Recruitment occurred between October 31, 2014, and February 1, 2018, with follow-up through February 1, 2019. Participants were enrolled using a 3-phase procedure to minimize participant burden and increase efficiency.^12^ In phase 1, site coordinators consulted a referring clinician to establish provisional PTSD diagnosis and other eligibility criteria. In phase 2, coordinators obtained participant consent, administered questionnaire assessments, read a standardized description of each treatment, and gave participants a brochure describing the treatments. In phase 3, participants completed a telephone assessment to establish eligibility.

### Assessment

Participants were assessed at baseline, during treatment, after completing treatment, and at 3- and 6-month follow-ups. Independent doctoral-level assessors at 2 centralized sites who were blinded to treatment condition administered CAPS-5 and SCID-5 telephone interviews and questions about suicidal and homicidal ideation, treatment preference, and current medications (also assessed from clinical records). Questionnaire measures were obtained at each site. Data were transmitted electronically to the centralized coordinating center.

### Treatment

Treatment was delivered in outpatient clinics. There were 12 weekly sessions, but participants could finish in 10 or 11 sessions if, beginning in session 8, they reported a PCL-5 score of 18 points or less in 2 consecutive sessions. Participants with PCL-5 scores of 38 points or greater at session 12 could receive up to 2 additional sessions. Participants also could have 2 nonprotocol sessions to address stressors that presented obstacles to study participation.^28^ Standard PE sessions were 90 minutes, and standard CPT sessions were 60 minutes. Because this was a practical trial, we did not equate session duration.

#### PE Intervention

In PE, the primary components are in vivo and imaginal exposure followed by processing imaginal experience.^29^ In vivo exposure consists of gradually and systematically having patients approach distressing trauma-related situations, places, and people that have been avoided and remaining in the situation until distress reduces by half. Imaginal exposure involves repeated revisiting of the trauma memory and recounting aloud the traumatic events in detail, while vividly imagining the events. Treatment sessions are audio-recorded and patients are asked to listen to recordings daily between sessions. Psychoeducation and controlled breathing exercises are also included.

#### CPT Intervention

CPT consisted of cognitive therapy and writing 2 trauma accounts (now an optional component in the newest version of CPT^30^). Patients briefly process their trauma by writing an account of the event that they read to themselves and to therapists after sessions 3 and 4. Most of the sessions help patients challenge their beliefs through Socratic dialogue and use of progressive daily worksheets. The initial focus is on challenging beliefs caused by hindsight bias, just world violations, and self-blame or erroneous other-blame and then shifts to overgeneralized beliefs about self, others, and the world. Narrative statements about the causes and impact of the trauma are written at the beginning and end of therapy to begin to identify problematic thoughts and allow patients to see changes in their thinking.

### Therapy Supervision and Fidelity Monitoring

By design, there were 4 CPT and 4 PE therapists at each site; actual numbers fluctuated owing to therapist turnover. A total of 142 master’s- and doctoral-level therapists participated, and they all completed required VA training and supervision in CPT or PE. Before treating study participants, therapists watched 4 hours of training videos, participated in a 1-day online training, and demonstrated adequate therapy fidelity on 2 audiotapes of prior treatment sessions. Most therapists delivered only 1 treatment, but 4 switched during the study to accommodate site needs (1 therapist treated 1 patient in each treatment group simultaneously). Therapists participated in weekly group consultation calls and could receive individual supervision if needed.

All sessions were audio-recorded. An independent expert clinician rated fidelity for 2 randomly-sampled sessions from each therapist (1 therapist had only one available recording). CPT and PE did not differ in global ratings of adherence or competence, which had ranged from means of 4.40 to 4.67, between very good (4) and excellent (5).

### Statistical Analysis

The study biostatistician (B.K.C.) performed all analyses. Baseline characteristics were compared using χ^2^ tests or 2-sample *t* tests. All analyses were performed on the intention-to-treat sample of randomized participants. We attempted to assess all participants regardless of treatment dropout. Multiple imputation^31^ was conducted using PROC MI and MI ANALYZE in SAS statistical software version 9.4 (SAS Institute) with the Markov chain using Monte Carlo method^32^ to impute missing values.

Outcomes were analyzed using a generalized linear mixed model using SAS PROC MIXED and PROC GLIMMIX in SAS. The analysis for each outcome consisted of a longitudinal model including therapist as a random cluster effect and baseline severity, treatment group, time, site, and the treatment × time interaction as fixed effects. For brevity, we do not report treatment × time interactions because none were significant. Longitudinal analyses were supplemented by cross-sectional comparisons.

Within- and between-groups effect sizes were computed as *d*, the SMD. Using the variance estimate and intraclass correlation within therapist from a prior PE study,^12^ and assuming that each therapist would treat 8 patients, we estimated that 900 participants would be needed to have 90% power at 2-tailed *P* = .05 to detect an SMD of 0.25, reasoning anything smaller would be clinically insignificant. Data were analyzed from October 5, 2020, to May 5, 2021.

## Results

Analyses were based on all 916 randomized participants (730 [79.7%] men and 186 [20.3%] women; mean [range] age 45.2 [21-80] years). Most veterans served in the Iraq or Afghanistan Wars (530 participants [57.9%]). There were 249 Black participants (27.1%) and 590 White participants (64.4%), and 139 participants (15.2%) were Hispanic. Most participants were unemployed (534 participants [58.3%]) (Table 1). A total of 455 participants were randomized to PE, and 461 participants were randomized to CPT. Participants reported exposure to a mean of 7.7 (95% CI, 7.4-7.9) traumatic events in the PE group and 7.4 (95% CI, 7.2-7.7) traumatic events in the CPT group. More than 70% of participants in both groups reported combat exposure (PE: 357 participants [78.5%]; CPT: 347 participants [75.3%]) and just over one-third reported sexual trauma (PE: 166 participants [36.5%]; CPT: 163 participants [35.4%]). Almost 80% of participants had a current comorbid psychiatric disorder (PE: 343 participants [75.4%]; CPT: 371 participants [80.5%]), and more than 90% of participants had a lifetime history of comorbid psychiatric disorder (PE: 417 participants [91.7%]; CPT: 424 participants [92.0%]) (Table 1). Severity of PTSD and other symptoms was high, with a mean CAPS-5 score at baseline of 39.9 (95% CI, 39.1-40.7) points in the PE group and 40.3 (95% CI, 39.5-41.1) points in the CPT group. Groups did not differ at baseline, except that the CPT group was more likely to have a lifetime history of anxiety disorder. Half of each group preferred the treatment to which they had been assigned. Treatment credibility and expectancy of benefit were high and did not differ between groups. After treatment and during follow-up, 326 participants (71.6%) in the PE group and 334 participants (72.5%) in the CPT group participated in outcome measurement (Figure).

**Table 1.** Participant Characteristics at Baseline

Characteristic	No. (%) (N = 916)	
	PE (n = 455)	CPT (n = 461)
Gender		
Men	361 (79.3)	369 (80.0)
Women		
Age, mean (95% CI), y	45.5 (44.3-46.8)	44.9 (43.7-46.1)
Service era^a^		
Vietnam	82 (18.0)	77 (16.7)
Gulf War	85 (18.7)	87 (18.9)
OEF/OIF/OND	260 (57.1)	270 (58.6)
Other	66 (14.5)	59 (12.8)
>High school education	216 (47.5)	192 (41.7)
Unemployed	271 (59.6)	263 (57.1)
Married or cohabitating	246 (54.1)	237 (51.4)
Race^b^		
American Indian or Alaskan Native	18 (4.0)	15 (3.3)
Asian	14 (3.1)	11 (2.4)
Black	119 (26.2)	130 (28.2)
Native Hawaiian or Pacific Islander	7 (1.5)	10 (2.2)
White	301 (66.1)	289 (62.7)
Other	21 (4.6)	25 (5.4)
Spanish, Hispanic or Latino ethnicity	67 (14.8)	72 (15.6)
Positive VA screen		
Military sexual trauma	134 (29.4)	133 (28.9)
Traumatic brain injury	294 (64.6)	281 (61.0)
Lifetime trauma exposure		
Mean (95% CI), No.	7.7 (7.4-7.9)	7.4 (7.2-7.7)
Combat exposure	357 (78.5)	347 (75.3)
Any sexual trauma	166 (36.5)	163 (35.4)
Physical assault	386 (84.8)	408 (88.5)
Disaster exposure	391 (85.9)	385 (83.5)
Serious accident	385 (84.6)	389 (84.4)
Life-threatening illness or injury	154 (33.9)	163 (35.4)
Other traumatic event	371 (81.5)	354 (76.8)
PTSD disability claim		
Approved	186 (41.1)	202 (44.0)
Pending	116 (25.6)	129 (28.1)
Denied	26 (5.7)	19 (4.1)
Never applied	125 (28)	109 (24)
Approved PTSD disability percentage^c^	54.2 (22.7)	54.9 (24.8)
Receiving psychotherapy^d^	95 (20.9)	98 (21.3)
Using psychotropic medication^d^	303 (66.6)	317 (68.8)
Current comorbid psychiatric disorder		
Any	343 (75.4)	371 (80.5)
Mood disorder	309 (67.9)	332 (72.0)
Anxiety disorder	139 (30.6)	166 (36.0)
Substance use disorders	32 (7.0)	40 (8.7)
Obsessive compulsive disorder	19 (4.2)	29 (6.3)
Lifetime comorbid psychiatric disorder		
Any	417 (91.7)	424 (92.0)
Mood disorder	398 (87.5)	400 (86.8)
Anxiety disorder	149 (32.8)	181 (39.3)
Substance use disorders	130 (28.6)	112 (24.3)
Obsessive compulsive disorder	24 (5.3)	36 (7.8)
CAPS-5 score, mean (95% CI)	39.9 (39.1-40.7)	40.3 (39.5-41.1)
Posttraumatic Diagnostic Scale, mean (95% CI)	50.7 (49.5-52.0)	50.5 (49.3-51.7)
BDI-II, mean (95% CI)		
Overall	30.3 (29.4-31.3)	30.0 (29.0-30.9)
Suicidality^e^	163 (35.9)	156 (33.8)
STAI, mean (95% CI)		
State anger	17.8 (17.1-18.5)	17.9 (17.3-18.6)
Trait anger	24.1 (23.5-24.8)	24.2 (23.6-24.8)
Anger expression	37.3 (36.3-38.3)	36.4 (35.5-37.4)
BAM, mean (95% CI)^f^	0.8 (0.6-0.9)	0.8 (0.7-0.9)
SIP-R, mean (95% CI)	3.5 (2.7-4.3)	3.3 (2.5-4.1)
World Health Organization Disability Adjustment Scale-II, mean (95% CI)	29.4 (28.6-30.3)	29.7 (28.9-30.5)
WHOQoL-BREF, mean (95% CI)		
Physical health	44.4 (43.4-45.3)	43.7 (42.7-44.7)
Psychological	46.5 (45.2-47.8)	46.4 (45.2-47.7)
Social relationships	41.3 (39.4-43.3)	40.6 (38.7-42.5)
Environment	58.2 (56.6-59.7)	57.4 (55.9-59.0)
Prefer PE treatment	232 (51.6)	214 (46.8)
Credibility and Expectancy Questionnaire, mean (95% CI)	20.9 (20.3-21.5)	21.8 (21.2-22.3)

Abbreviations: BAM, Brief Addiction Monitor; BDI-II, Beck Depression Inventory-II; CAPS-5, Clinician-Administered PTSD Scale for *DSM-5*; CPT, cognitive processing therapy; OEF, Operation Enduring Freedom; OIF, Operation Iraqi Freedom; OND, Operation New Dawn; PE, prolonged exposure; PTSD, posttraumatic stress disorder; SIP-R, Short Inventory of Problems–Revised; STAI, Spielberger State Trait Anxiety Inventory; WHOQoL-BREF, World Health Organization Quality of Life.

^a^ Service era was coded by including any Vietnam, Gulf, or OEF/OIF veteran in their respective categories (including if they served in more than one era, eg, Vietnam and Gulf). If a veteran did not serve in Vietnam, Gulf, or OEF/OIF, they were coded as other.

^b^ Participants self-reported their race and could report more than 1. Other race included biracial/mixed, Puerto Rican, Hispanic, Spanish, Latino, Mexican, Moor, Creole, New Native, Caribbean, European, Romanian, Persian, Estonian, and declined to report.

^c^ Refers to the mean percentage of time (0%-100%) of approved service-connected disability compensation related to PTSD diagnosis.

^d^ Within 6 months prior to study enrollment.

^e^ Suicidality was coded by grouping “I have thoughts of killing myself, but I would not carry them out,” “I would like to kill myself,” and “I would kill myself if I had the chance” from item 9 of the BDI-II together as endorsing suicidality.

^f^ The Brief Addiction Monitor scores number of days drinking more than 5 drinks and number of days using illegal drugs converted into points,^20^ where a higher number of points indicates greater substance use.

Table 2 provides information about treatment participation and satisfaction. CPT participants attended a mean of 9.1 (8.7-9.5) sessions, 1 more session than PE participants, who attended a mean of 8.2 (95% CI, 7.8-8.6) sessions. Dropout was higher in PE (254 participants [55.8%]) than in CPT (215 participants [46.6%]; χ^2^ = 7.73; *P* = .005). CPT participants were more likely to complete in 12 sessions (115 participants [25.3%]), whereas PE participants were more likely to be early completers (55 participants [12.1%]). Few participants in either group needed additional sessions. Less than 15% of participants used stressor sessions. Satisfaction at the end of treatment was high and did not differ between CPT and PE.

**Table 2.** Treatment Characteristics

Characteristic	No. (%) (N = 916)	
	Prolonged exposure	Cognitive processing therapy
Total sessions, mean (95% CI), No.	8.2 (7.8-8.6)	9.1 (8.7-9.5)^a^
Treatment dropout^b^	254 (55.8)	215 (46.6)^c^
Completed early owing to therapist error^d^	7 (1.5)	3 (0.7)
Completed 12 session	115 (25.3)	189 (41.0)^a^
Completed early^d^	55 (12.1)	22 (4.8)^a^
Received extra sessions^e^	31 (6.8)	35 (7.6)
Used a stressor session	71 (15.6)	62 (13.4)
Stressor sessions among patients using a stressor session, mean (95% CI), No.	1.18 (1.09-1.28)	1.05 (0.99-1.10)^a^
Client Satisfaction Questionnaire score^f^	1.5 (1.4-1.6)	1.5 (1.4-1.6)

Abbreviations: CPT, cognitive processing therapy; PE, prolonged exposure.

^a^ *P* < .05.

^b^ Dropout includes all patients who ended before 10 sessions, or otherwise ended treatment not according to study protocol or did not start treatment at all.

^c^ *P* < .01.

^d^ Early completion includes patients who ended at 10 or 11 sessions according to the study protocol for early completion.

^e^ Extra sessions includes patients who had 13 or 14 treatment sessions according to the study protocol for extra sessions.

^f^ Client satisfaction was a self-reported rating of satisfaction with the received treatment on a 4-point Likert scale, with lower numbers reflecting higher satisfaction. *P* values reflect the comparison between PE and CPT.

### Primary Outcome Analyses

PTSD severity on the CAPS-5 improved substantially in both PE (SMD, 0.99) and CPT (SMD, 0.71) groups from before to after treatment (Table 3). Overall improvement was greater in PE than CPT, but the effect size of the difference was small (SMD, 0.17) and the absolute difference was not clinically significant (least square mean, 2.42 [95% CI, 0.53-4.31] points; *P* = .01) (Table 3; eFigure in Supplement 2). PE had better outcomes than CPT at posttreatment and the 3-month follow-up, but not at the 6-month follow-up (Table 3). Because of the high and differential attrition, we performed sensitivity analysis for the primary outcome assuming that data were not missing at random. Results were comparable to the primary findings showing greater improvement in PE (least square mean, 2.15 [95% CI, 0.34-3.96]; *P* = .02).

**Table 3.** Outcomes as a Function of Treatment Group

Measure	Pre-post effect size^a^		Between-groups, effect size^b^	Posttreatment^b^		3 mo^b^		6 mo^b^	
	PE	CPT		PE	CPT	PE	CPT	PE	CPT
CAPS-5	0.99^c^	0.71^c^	0.17^d^	24.3 (22.8-25.2)	27.2 (25.5-28.9)^e^	26.4 (25.1-27.8)	28.7 (27.2-30.2)^d^	24.8 (23.2-26.2)	26.9 (25.4-28.4)
PDS-5	0.74^c^	0.64^c^	0.17^d^	33.5 (31.3-35.6)	36.7 (34.7-38.7)^d^	34.5 (32.5-36.4)	37.5 (35.6-39.4)^d^	33.6 (31.6-35.5)	36.7 (34.8-38.7)^d^
BDI	0.51^c^	0.50^c^	0.08	22.0 (20.5-23.5)	22.7 (21.3-24.1)	22.2 (20.9-23.5)	23.6 (22.3-24.9)	21.9 (20.6-23.3)	22.9 (21.5-24.2)
STAI	0.39^c^	0.34^c^	0.07	93.4 (91.4-95.4)	94.6 (92.7-96.5)	93.2 (91.2-95.3)	94.7 (92.8-96.6)	93.2 (91.1-95.4)	94.5 (92.6-96.4)
SIP-R	0.10^d^	0.12^d^	0.07	2.61 (1.89-3.34)	2.49 (1.76-3.23)	2.42 (1.58-3.25)	2.29 (1.60-2.97)	3.09 (2.22-3.95)	1.72 (0.93-2.51)^d^
BAM	0.07	0.05	0.06	0.88 (0.73-1.03)	0.86 (0.69-1.02)	1.0 (0.84-1.16)	0.94 (0.78-1.1)	0.96 (0.82-1.11)	0.80 (0.64-0.97)
WHO-DAS-II	0.11^d^	0.11^d^	0.03	28.6 (27.7-29.4)	28.6 (27.8. 29.4)	28.0 (27.1-29.0)	28.7 (27.7-29.7)	28.5 (27.5-29.4)	28.5 (27.4-29.5)
WHOQoL-BREF									
Physical health	0.18^c^	0.19^c^	0.004	47.0 (44.5-49.5)	48.6 (46.0-51.1)	47.0 (45.7-48.2)	47.0 (45.8-48.2)	47.1 (45.8-48.4)	46.9 (45.6-48.2)
Psychological	0.15^c^	0.14^e^	0.04	49.0 (47.5-50.5)	48.9 (47.3-50.5)	49.1 (47.7-50.5)	48.1 (46.7-49.4)	49.5 (47.9-51.1)	49.1 (47.6-50.7)
Social relationships	0.17^e^	0.12^d^	0.06	45.4 (43.1-47.8)	43.9 (41.6-46.3)	46.2 (43.6-48.8)	44.1 (41.6-46.6)	46.2 (43.8-48.6)	45.5 (42.8-48.2)
Environment	0.17^c^	0.08	0.10	61.1 (59.3-62.9)	59.5 (57.6-61.3)	61.5 (59.8-63.2)	59.8 (58.2-61.4)	62.3 (60.5-64.1)	60.9 (59.2-62.6)

Abbreviations: BAM, Brief Addiction Monitor; BDI, Beck Depression Inventory; CAPS-5, Clinician-Administered PTSD Scale for *DSM-5*; CPT, cognitive processing therapy; PDS-5, Posttraumatic Diagnostic Scale; PE, prolonged exposure; PTSD, posttraumatic stress disorder; SIP-R, Short Inventory of Problems–Revised; STAI, Spielberger State Trait Anxiety Inventory, State subscale; WHO-DAS-II, World Health Organization Disability Assessment Schedule; WHOQoL-BREF, World Health Organization Quality of Life.

^a^ Pre-post effect sizes (Cohen *d*) were calculated from analyses to generate least squares means for within-groups comparisons.

^b^ Between-groups comparisons. *P* values at each assessment point reflect the comparison between PE and CPT.

^c^ *P* < .001.

^d^ *P* < .05.

^e^ *P* < .01.

At posttreatment, 332 PE participants (73.0%) and 277 CPT participants (60.1%) had responded (Table 4). The overall odds of response (odds ratio [OR], 1.35 [95% CI, 1.06-1.65]; *P* < .001), loss of diagnosis (OR, 1.46 [95% CI, 1.11-1.80]; *P* < .001), and remission (OR, 1.63 [95% CI, 1.26-2.00]; *P* < .001) were higher in PE than in CPT, differences that were observed at all posttreatment assessments.

**Table 4.** Response, Loss of Diagnosis, and Remission in PE and CPT Groups

Outcome	Overall treatment effect, OR (95% CI)^a^	No. (%)					
		Posttreatment		3 mo		6 mo	
		PE (n = 455)	CPT (n = 461)	PE (n = 455)	CPT (n = 461)	PE (n = 455)	CPT (n = 461)
Response^b^	1.32 (1.00-1.65)^c^	332 (73.0)	277 (60.1)^c^	293 (64.4)	258 (56.0)^d^	328 (72.1)	299 (64.9)^e^
Loss of diagnosis^f^	1.43 (1.12-1.74)^c^	184 (40.4)	130 (28.2)^c^	152 (33.4)	110 (23.9)^d^	171 (37.6)	134 (28.9)^d^
Remission^g^	1.62 (1.24-2.00)^c^	93 (20.4)	58 (12.6)^c^	62 (13.6)	43 (9.3)^e^	85 (18.7)	55 (11.9)^e^

Abbreviations: CPT, cognitive processing therapy; OR, odds ratio; PE, prolonged exposure.

^a^ ORs were calculated with CPT as the reference group and reflect the overall main effect of treatment across all outcome assessments (posttreatment, 3-months, and 6-months).

^b^ Defined as an improvement of at least 10 points in severity.

^c^ *P* < .001 between PE and CPT.

^d^ *P* < .01 between PE and CPT.

^e^ *P* < .05 between PE and CPT.

^f^ Defined as response, plus no longer meeting *Diagnostic and Statistical Manual of Mental Disorders (Fifth Edition)* symptom criteria and severity less than 25.

^g^ Defined as loss of diagnosis plus severity less than 12.

### Secondary Outcome Analyses

Pre-post effect sizes showed improvement from before to after treatment in all outcomes in PE and CPT (Table 3), except for heavy drinking or drug use in both groups and environmental quality of life in CPT. There was a small (SMD, 0.17) but statistically significant overall greater improvement in PE than in CPT for self-reported PTSD severity on the PDS (least square mean, 3.14 [95% CI, 0.7-5.16] points) that was observed at all time points. Treatments did not differ on other measures.

### Safety

The eTable in Supplement 2 provides details about serious adverse events (SAEs). Few events, and no deaths or suicide attempts, were attributed or possibly attributed to treatment. PE and CPT did not differ in SAEs except psychiatric hospitalization was more likely in CPT (23 participants [5.0%]) than PE (9 participants [2.0%]; χ^2^ = 6.16; *P* = .01). Three participants in PE and 1 participant in CPT were withdrawn from treatment owing to SAEs. An additional 3 participants in PE and 3 participants in CPT dropped out owing to SAEs that were hospitalizations for physical illnesses unrelated to study treatment. PE and CPT did not differ in the number of participants whose CAPS-5 scores worsened by 10 points or more at posttreatment.

## Discussion

To our knowledge, this randomized clinical trial of PE and CPT is the largest study of psychotherapy for PTSD ever conducted. Both treatments resulted in meaningful decreases in clinician-rated PTSD severity, the primary outcome. PE was more effective than CPT, but the difference was not clinically significant. There were comparable findings for self-reported PTSD severity. PE was more likely to result in treatment response, loss of diagnosis, and remission, but owing to administrative error, these outcomes were not preregistered and therefore must be interpreted with caution. Treatments did not differ on measures of other symptoms, functioning, or quality of life. The fact that we observed a difference for PTSD symptoms when a prior study by Resick et al^8^ did not is likely owing to our higher statistical power.

The greater effects in PE were not explained by higher therapist adherence or competence. A possible reason that PE had better outcomes is that PE sessions were 90 minutes long, whereas CPT sessions were 60 minutes. Although the amount of treatment received in PE was lessened by higher dropout, PE participants still had more minutes of care. However, we do not think this difference is a likely explanation for our findings. A 2015 randomized clinical trial^33^ that varied session length in PE found that 60-minute sessions were statistically noninferior to 90-minute sessions, which suggests that our results would have been comparable if we had used 60-minute PE sessions. In addition, research on dose-response in psychotherapy does not indicate that more treatment is necessarily better. Results are inconsistent regarding whether more sessions yield better outcomes, and having fewer sessions is associated with faster response.^34,35^

The PE group had higher treatment dropout than the CPT group, although the PE group also had more early completers. The relatively high treatment dropout in both groups was comparable with dropout for PE or CPT groups in other recent studies with veterans.^10^ We might have had high dropout because our sample was clinically realistic, with high severity and multiple comorbidities, and study therapists were clinicians who did not receive the amount of specialized training and supervision that is typical in psychotherapy efficacy trials. Also, we defined dropout strictly, as failure to complete 100% of protocol sessions. Another possible explanation is the high percentage (58%) of Iraq and/or Afghanistan War veterans, who are more likely than other veterans to drop out of PE and CPT in VA care.^36^

Despite high dropout, the amount of improvement in both treatment groups was meaningful and comparable with that observed in recent studies of veterans and military personnel.^37,38^ Concerns have been raised about the effectiveness of guideline-recommended treatments, such as PE and CPT, for veterans and military personnel.^10^ One systematic review and meta-analysis by Kitchiner et al^11^ concluded that these treatments are effective but noted their lower effectiveness and higher dropout in military and veteran samples relative to nonveterans. Kitchiner et al called for research to develop and evaluate more effective treatments for military personnel and veterans. We agree with the need to obtain better outcomes and suggest incorporating other strategies, such as measurement-based care, decision aids and shared decision-making, and telehealth,^39^ to improve benefit. An additional strategy is treatment matching. A recent article by Neria^40^ suggested that diagnostic heterogeneity in PTSD may limit treatment effectiveness. Identifying which treatment is optimal for which patient could enhance outcome. To do that, well-powered studies of treatment moderators are needed.

In our study. psychiatric SAEs were infrequent, with few (and no suicide attempts) attributed or possibly attributed to treatment. There also was little symptom worsening during treatment. PE and CPT were comparable in terms of safety, except that psychiatric hospitalization was more likely in CPT. However, the 5.0% occurrence in CPT is similar to the overall 4.2% in VA patients with PTSD.^6^

### Limitations

This study has some limitations. Participants were veterans, most with comorbidity and functional impairment; therefore, results might not generalize to nonveterans or patients with less complex conditions. Results may not generalize to women because 80% of participants were men. Dropout was high, which may have attenuated the potential benefits of the treatments. Also, the need to impute outcome data for 28% of participants could have impacted findings, although sensitivity analyses suggested that the primary results are robust.

## Conclusions

The findings of this randomized clinical trial support the VA’s strategy of promoting PE and CPT^1^ and reinforce guideline recommendations for these treatments as front-line therapies.^2,3^ Given that the difference on the primary outcome was not clinically significant, lack of differences between treatments on outcomes other than PTSD, and higher attrition in PE, we do not believe our findings support a recommendation for PE over CPT. Clinicians and systems of care may prioritize the categorical outcomes of response, loss of diagnosis, and remission because these outcomes have benefit at the population level. In contrast, patient preferences may be more influenced by treatment characteristics, such as session content and homework. We recommend shared decision-making to help patients understand the evidence and select their preferred treatment.

## Notes

Supplement 1.
Trial Protocol and Statistical Analysis Plan

Supplement 2. **eTable.** Serious Adverse Events **eFigure.** Posttraumatic Stress Disorder Symptom Severity on the CAPS-5 as a Function of Treatment Group

Supplement 3.
Data Sharing Statement
