# Supplementary material for: Comparison of Prolonged Exposure vs Cognitive Processing Therapy for Treatment of Posttraumatic Stress Disorder Among US Veterans A Randomized Clinical Trial
Source: JAMA Netw Open. Author manuscript; Available in PMC 2022 Feb 4. (PMC8771295; doi:10.1001/jamanetworkopen.2021.36921)
Supplement: Supplement 3 — Data Sharing Statement [file NIHMS1773712-supplement-Supplement_3.pdf]

Data Sharing Statement

Schnurr. Comparison of Prolonged Exposure vs Cognitive Processing Therapy for Treatment of Posttraumatic Stress Disorder Among US Veterans. *JAMA Netw Open*. Published January 19, 2022. doi:10.1001/jamanetworkopen.2021.36921

Data
Data available: No

Additional Information
Explanation for why data not available: Data are available to VA investigators who obtain a signed data use agreement from the VA Cooperative Studies Program. Because the study was approved and initiated before VA implemented its current data sharing policies, the consent form did not include consent language for open data sharing. We are glad to accommodate requests for data by collaboration on secondary analyses when feasible.
