# Supplementary material for: Comparison of Prolonged Exposure vs Cognitive Processing Therapy for Treatment of Posttraumatic Stress Disorder Among US Veterans A Randomized Clinical Trial
Source: JAMA Netw Open. Author manuscript; Available in PMC 2022 Feb 4. (PMC8771295; doi:10.1001/jamanetworkopen.2021.36921)
Supplement: Supplement 2 — eTable. Serious Adverse Events eFigure. Posttraumatic Stress Disorder Symptom Severity on the CAPS-5 as a Function of Treatment Group [file NIHMS1773712-supplement-Supplement_2.pdf]

Supplemental Online Content

Schnurr PP, Chard KM, Ruzek JI, et al. Comparison of prolonged exposure vs cognitive processing therapy for treatment of posttraumatic stress disorder among US veterans: a randomized clinical trial. *JAMA Netw Open*. 2022;5(1):e2136921. doi:10.1001/jamanetworkopen.2021.36921

eTable. Serious Adverse Events
eFigure. Posttraumatic Stress Disorder Symptom Severity on the CAPS-5 as a Function of Treatment Group

This supplemental material has been provided by the authors to give readers additional information about their work.

© 2022 Schnurr PP et al. *JAMA Network Open.*

eTable. Serious Adverse Events

	Prolonged Exposure	Cognitive Processing Therapy
	Patients with SAEs (Total Events)	Patients with SAEs (Total Events)
Total SAEs	55 (78)	63 (97)
Attributed	1 (2)	2 (2)
Possibly attributed	3 (3)	9 (9)
SAE type: Death	1 (1)	2 (2)
Attributed	0 (0)	0 (0)
Possibly attributed	0 (0)	0 (0)
SAE type: Psychiatric Hospitalization	9 (13)	23 (29)
Attributed	0 (0)	1 (1)
Possibly attributed	1 (1)	6 (6)
SAE type: Suicide Attempts	2 (5)	3 (3)
Attributed	0 (0)	0 (0)
Possibly attributed	0 (0)	0 (0)

Note. Adverse events were recorded from patient reports during treatment and assessment visits, and review of VA electronic medical records through 30 days post-6-month follow-up. Prolonged Exposure and Cognitive Processing Therapy did not differ in SAEs except more Cognitive Processing Therapy participants experienced psychiatric hospitalization, $\chi^2 = 6.16$, $p < .05$.

© 2022 Schnurr PP et al. *JAMA Network Open.*

eFigure. Posttraumatic Stress Disorder Symptom Severity on the CAPS-5 as a Function of Treatment Group

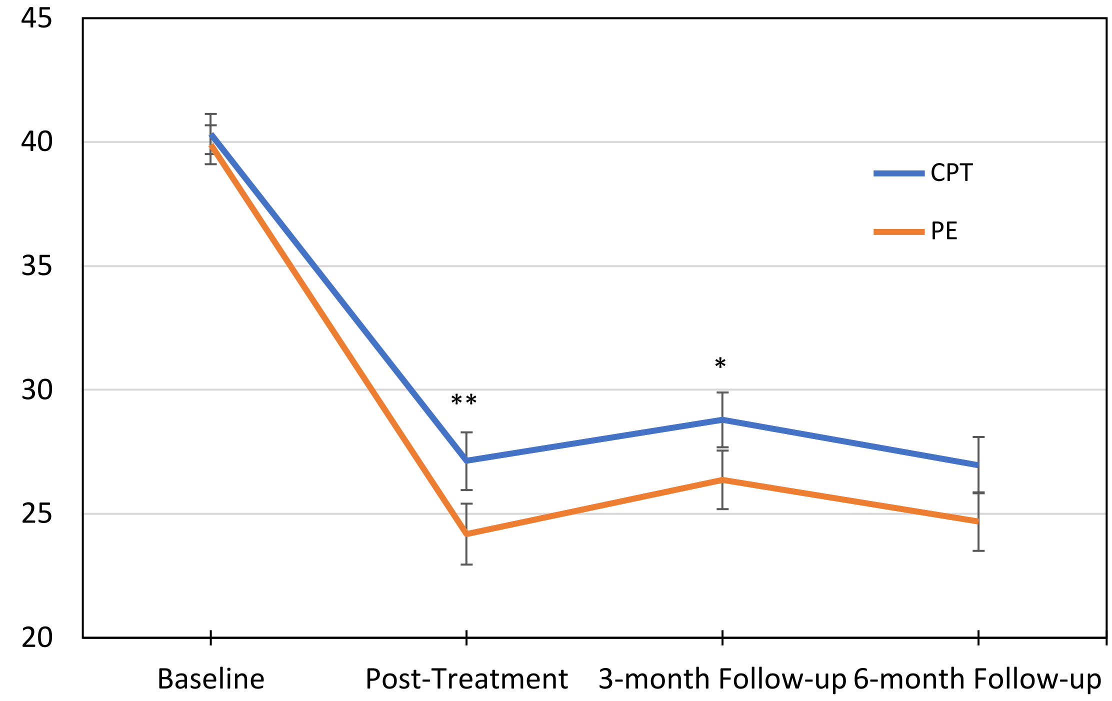

© 2022 Schnurr PP et al. *JAMA Network Open.*
