# Supplementary material for: Comparison of Prolonged Exposure vs Cognitive Processing Therapy for Treatment of Posttraumatic Stress Disorder Among US Veterans A Randomized Clinical Trial
Source: JAMA Netw Open. Author manuscript; Available in PMC 2022 Feb 4. (PMC8771295; doi:10.1001/jamanetworkopen.2021.36921)
Supplement: Supplement 1 — Trial Protocol and Statistical Analysis Plan [file NIHMS1773712-supplement-Supplement_1.pdf]

**VA Cooperative Study No. 591**

# Comparative Effectiveness Research in Veterans with PTSD

# (CERV-PTSD)


**Study Co-Chairs:**
**Paula P. Schnurr, PhD**
**VAMC, White River Junction**

**Kathleen M. Chard, PhD**
**VAMC Cincinnati**

**Josef I. Ruzek, PhD**
**VA Palo Alto Healthcare System**

**Cooperative Studies Program Coordinating Center, Palo Alto VA:**
**Bruce Chow, MS, Study Biostatistician**
**Mei-Chiung Shih, PhD, Senior Biostatistician**
**Lisa Zehm, MS, Study Project Manager**

**Cooperative Studies Program**
**Clinical Research Pharmacy Coordinating Center, Albuquerque VA:**
**Amanda Snodgrass, PharmD, Study Adverse Event Specialist**



**February 16, 2018**
**Version 11.0**



**PRIVILEGED AND CONFIDENTIAL**

This page intentionally left blank.

# TABLE OF CONTENTS


**I. BACKGROUND AND SIGNIFICANCE ........ 1**
A. Introduction ........ 1
B. Treatment of PTSD ........ 1
C. Comparative Effectiveness of Prolonged Exposure and Cognitive Processing Therap ........ 4
D. Importance of the Proposed Research to VA ........ 8
E. Feasibility of a Cooperative Study within the VA ........ 10
F. Summary ........ 14
**II. SPECIFIC OBJECTIVES ........ 16**
A. Primary Objective ........ 16
B. Secondary Objective ........ 16
C. Tertiary Objective ........ 16
D. Exploratory Analyses ........ 16
**III. SUMMARY OF STUDY DESIGN ........ 17**
A. Study Population ........ 17
B. Study Treatments ........ 18
C. Outcome Measures ........ 18
D. Sample Size ........ 18
E. Study Monitoring ........ 18
**IV. PATIENT POPULATION AND PATIENT RECRUITMENT ........ 19**
A. Inclusion and Exclusion Criteria ........ 19
B. Recruitment ........ 19
C. Screening and Consent ........ 23~~21~~
**V. TREATMENT ........ 26~~24~~**
A. Prolonged Exposure ........ 26~~24~~
B. Cognitive Processing Therapy ........ 29~~26~~
**VI. TREATMENT ASSIGNMENT ........ 31~~29~~**
A. Randomization ........ 31~~29~~
B. Blinding ........ 32~~29~~
**VII. MEASURES ........ 33~~31~~**
A. Screening and Eligibility ........ 33~~31~~
B. Primary Outcome ........ 35~~33~~
C. Secondary Outcomes ........ 35~~33~~

**VIII. STUDY PROCEDURES** .......... **38~~35~~**
A. Assessment .......... 38~~35~~
B. Treatment .......... 43~~40~~
C. Discontinuation of Study Treatment .......... 49~~46~~
D. Withdrawals .......... 50~~47~~
E. Post Follow-up Procedures .......... 50~~47~~
F. Training and Supervision .......... 50~~47~~
G. Therapy Fidelity Monitoring .......... 51~~48~~
**IX. DATA COLLECTION AND MANAGEMENT** .......... **51~~48~~**
A. Data Management .......... 52~~49~~
B. Data Security .......... 53~~50~~
C. Proposed Data Collection Forms .......... 54~~51~~
**X. BIOSTATISTICAL CONSIDERATIONS** .......... **55~~52~~**
A. Expected Treatment Effect .......... 55~~52~~
B. Sample Size and Power Considerations .......... 56~~53~~
C. Number of Participating Sites and Duration of Study .......... 59~~56~~
D. Final Statistical Analysis .......... 60~~57~~
E. Interim Analysis .......... 63~~60~~
F. Procedure for Handling Missing Data .......... 65~~61~~
G. Procedures for Reporting Modifications to the Original Statistical Plan .......... 65~~62~~
**XI. MONITORING AND REPORTING ADVERSE EVENTS** .......... **66~~63~~**
A. Importance of Adverse Event Reporting .......... 66~~63~~
B. Role of the Local Site Investigator in Adverse Event Monitoring .......... 66~~63~~
C. Collection of Safety Information .......... 66~~63~~
D. Expedited Reporting of Serious Adverse Events .......... 70~~67~~
E. Reporting Adverse Events and Serious Adverse Events to the VA Central IRB .......... 70~~67~~
**XII. QUALITY CONTROL** .......... **71~~68~~**
A. Standardization/Validation of Measurements .......... 71~~68~~
B. Treatment .......... 71~~68~~
C. Masking .......... 71~~68~~
D. Monitoring Participant Intake and Probation/Termination of Participating Centers .......... 71~~68~~
**XIII. ORGANIZATION & ADMINISTRATION** .......... **72~~69~~**
**XIV. GOOD CLINICAL PRACTICE (GCP)** .......... **76~~73~~**
A. Role of GCP .......... 76~~73~~

B. Summary of Monitoring and Auditing Plans ........ 76~~73~~

**XV. PUBLICATIONS ........ 77~~74~~**

A. Publication Plan ........ 77~~74~~

B. Planned publications ........ 78~~75~~

**XVI. REFERENCES ........ 80~~77~~**

**APPENDICES**

**Appendix A. CSP 591 INFORMED CONSENT FORM MODEL**

**Appendix B. BIOSTATISTICAL AND RESEARCH DATA PROCESSING (BRDP)**

**Appendix C. RESEARCH DATA FORMS**

**Appendix D. OWNERSHIP, CONTROL AND ACCESS TO STUDY DATA**

**Appendix E. UPDATES TO THE PROTOCOL**

This page intentionally left blank.

# I. BACKGROUND AND SIGNIFICANCE

## A. Introduction

Posttraumatic stress disorder (PTSD) is a serious mental health problem in Veteran and non-Veteran populations. PTSD can develop following exposure to a traumatic event such as combat, assault, disaster, and accidents (American Psychiatric Association, 1994). Lifetime prevalence in US adults is higher in women (11.7%) than in men (4.0%) (Kessler et al., 2012) and is especially high among military Veterans (Kulka et al., 1990; Ramchand et al., 2010). According to a report by the RAND Corporation (Tanelian & Jaycox, 2008), the prevalence of PTSD is 14% in military personnel who served in Operations Enduring Freedom, Iraqi Freedom, or New Dawn (OEF/OIF/OND).

The symptoms of PTSD include re-experiencing the traumatic event, avoidance of stimuli associated with the event or numbing of general responsiveness, and increased arousal, with the increased arousal manifested by such symptoms as sleep disturbance, irritability or anger outbursts, and an exaggerated startle response. However, PTSD has much broader effects on the lives of individuals who develop it. PTSD is associated with a range of comorbid conditions and functional difficulties, including depression, substance abuse, anxiety disorders, psychosocial impairment, poor physical health, and greater service utilization (e.g., Kessler et al., 2005; Kulka et al., 1990; Schnurr et al., 2009). Without adequate treatment, PTSD can become chronic (Kessler et al., 1995), lasting even into old age (Kessler et al., 2012; Pietrzak et al., 2011; Schnurr et al., 2002). Unfortunately, individuals with PTSD are less likely than those with other common psychiatric disorders to seek treatment. The National Comorbidity Survey Replication (Wang et al., 2005) estimated that the cumulative lifetime probability of treatment contact was only 65.3% for PTSD, versus 88.1% for major depression and 95.3% for panic disorder. Time to initial contact was also substantially longer in PTSD than in these other disorders.

## B. Treatment of PTSD

### *1. Treatment effectiveness*

Practice guidelines for PTSD recommend Cognitive Behavioral Therapy (CBT), Eye Movement Desensitization and Reprocessing (EMDR), selective serotonin reuptake inhibitors (SSRIs), and the serotonin norepinephrine reuptake inhibitor (SNRI) venlafaxine as primary treatments (American Psychiatric Association Work Group on ASD and PTSD, 2004; Departments of

Veterans Affairs and Defense, 2010; Foa et al., 2008). CBT is a type of psychotherapy that uses systematic techniques based on learning theory and cognitive psychology to help patients identify and correct dysfunctional thoughts, behaviors, and emotions. Two of the most well-studied types of CBT for treating PTSD are being disseminated nationally across VA (Karlin et al., 2010): a type of exposure therapy known as Prolonged Exposure (PE; Foa et al., 2007) and a type of cognitive therapy known as Cognitive Processing Therapy (CPT; Resick et al., 2010)..

The evidence demonstrating the effectiveness of PE and CPT is particularly strong. A report by the Institute of Medicine (IOM; 2008) found that only CBT with an exposure component had sufficient evidence of effectiveness. However, both PE and CPT were classified in that report as exposure therapies, although CPT is predominantly a cognitive therapy. A more recent report by the Agency for Healthcare Research and Quality (AHRQ; 2012) that categorized PE and CPT separately found that evidence of effectiveness for exposure therapies (including PE) was strong for reducing PTSD and depression symptoms and moderate for achieving loss of PTSD diagnosis. The AHRQ report found the evidence was moderate for cognitive therapies (like CPT) and for mixed types of CBT. No other types of psychotherapy were judged to have moderate or better evidence for all three outcomes.

In contrast, the IOM (2008) found that the evidence for the effectiveness of medication was inconclusive, mostly due to the potential for bias introduced by extensive use in the available studies of the last-observation-carried-forward method of handling missing data. The AHRQ report found moderate evidence of effectiveness for the SSRI paroxetine and the SNRI venlafaxine for treating PTSD severity, depression severity, and loss of PTSD diagnosis.

*2. The need for comparative effectiveness research on treatments for PTSD*

A report by the IOM in 2009 set out a national agenda for comparative effectiveness research (CER), in response to a Congressional allocation of over $1 billion to facilitate optimal decisions about healthcare. There have been very few comparative effectiveness studies of treatments for PTSD, and none have been sufficiently large to have adequate power to compare active treatments. Consequently, the recent AHRQ report (2012) on PTSD treatment calls for studies that compare psychological treatments with the best evidence of efficacy, following a similar recommendation by the IOM (Institute of Medicine, 2008). The IOM report specifically mentioned the need for more research on the treatment of PTSD in military Veterans. Comparing PE to CPT would directly respond to these recommendations.

**Table I.1** presents information from the AHRQ report (2012) on between-group differences in reduction of PTSD symptom severity for all psychotherapies and medications that had moderate or high evidence of effectiveness across all outcomes examined. The data are presented as the difference in pre-post change between active treatment and control on the Clinician-Administered PTSD Scale (CAPS; Weathers et al., 2001), the gold standard for PTSD assessment.

***Table I.1. AHRQ findings for Treatments with Moderate or High Evidence Across Outcomes***

| Treatment | Strength of Evidence | Pre-post Difference in PTSD Severity vs. Controls (95% CI) |
|---|---|---|
| CBT–Cognitive Processing Therapy[a] | Moderate | -35.9 (-52.8 to -19.0) |
| CBT–Exposure[b] | High | -24.4 (-37.2 to -11.5) |
| CBT–Mixed | Moderate | -27.6 (-40.0 to -15.3) |
| Venlafaxine | Moderate | -7.2 (-11.0 to -3.3) |
| Paroxetine | Moderate | -12.6 (-15.7 to -9.5) |

*Note*. PTSD severity was measured on the Clinician-Administered PTSD Scale (Weathers et al., 2001). A difference of 10 points is considered to be the minimum indicating treatment response (Schnurr et al., 2001). [a]All 3 studies in the CBT-Cognitive Restructuring category for this outcome were Cognitive Processing Therapy (Chard et al., 2005; Monson et al., 2006; Resick et al., 2002). [b]Four of the 5 studies in the CBT-Exposure category for this outcome were Prolonged Exposure (Asukai et al., 2010; Resick et al., 2002; Rothbaum et al., 2005; Schnurr et al., 2007).

These data illustrate a consistent finding across other reviews: that psychotherapy is more effective than medication. It is difficult to directly compare the effectiveness of psychotherapy and medication because of differences in study design, particularly the type of control group. Whereas placebos are used in medication trials, psychotherapy studies use less active controls (waitlist) and more active controls (nonspecific treatment, treatment-as-usual) (Schnurr, 2007). However, it is unlikely that methodological factors completely account for the difference between the psychotherapy and medication findings.

### *3. Patient preferences*

Studies of patient preferences have found that patients prefer psychotherapy over medication for the treatment of PTSD. In a study that used equipoise-stratified randomization, Shalev et al. (2012) found that 43% declined to be randomized to medication, whereas only 3.3% declined cognitive therapy (63% of whom also declined medication) and 1.2% declined PE (67% of whom also declined medication). A recent randomized clinical trial of sertraline versus PE (Feeney et al., 2010) found that 61% of study participants preferred PE. In addition, discrepancy between a patient's preference and assigned group predicted lower response to both treatments. An ongoing study in the Netherlands found that only 4% of participants wanted medication, preferring PE (50%) or EMDR (46%) instead (van Minnen, 2012).

## C. Comparative Effectiveness of Prolonged Exposure and Cognitive Processing Therapy

### *1. Direct and indirect comparisons*

In contrast to the amount of evidence indicating the effectiveness of PE and CPT, there is almost no direct evidence about their effectiveness relative to one another. The only study to compare the treatments was a single-site trial in civilian female rape survivors (Resick et al., 2002). Both PE and CPT were highly effective but the effect size of the difference between them was neither clinically nor statistically significant ($d = 0.14$ favoring CPT). Follow-up of the sample an average of 6 years later found an effect size < .01 (Resick et al., 2012). However, with 62 participants per group, the study was not powered to detect an effect smaller than medium ($d = .50$; Cohen, 1988), which is unlikely for two highly effective treatments. Thus, the lack of difference between treatments is inconclusive.

Other findings suggesting that CPT is more effective than PE are similarly inconclusive. A meta-analysis currently under review (Watts et al., 2012) found that the standardized mean difference (vs. control) was nonsignificantly larger for CPT ($d = 1.69$, 95% CI = 1.27, 2.11) than for PE ($d = 1.38$, 95% CI = 0.90, 1.86). According to the AHRQ report (2012; **Table I.1**), which was based on fewer studies because the authors eliminated studies judged to have a high risk of bias, the decrease in PTSD severity scores on the CAPS between intervention and control was larger in CPT than in PE: -35.9 (-52.81, -18.97) in CPT versus -24.4 (-37.2, -11.5) in PE. However, confidence intervals for PE and CPT were overlapping in both studies. Also, the estimate for exposure in the AHRQ report did not include data from two trials that had found a substantial effect of exposure versus waitlist because these trials did not use the CAPS (Foa et al., 1999, 2005). In addition, the difference may be explained by the fact that all of the CPT studies included in both reviews used an untreated control group, which results in larger effects. In contrast, some of the PE studies used a treated control group; CSP #494 (Schnurr et al., 2007), which introduced significant heterogeneity in the AHRQ analysis, had a distinctively active comparison group.

Evaluation data from VA's rollouts (**Table I.2**) showing a larger effect size for CPT than for PE are difficult to interpret as well because key decisions about factors that could affect outcome (such as the criterion for determining treatment completion) are not standardized across treatments. Nevertheless, these data demonstrate that both treatments are effective in VA patients.

***Table I.2. Pre-post Change in PTSD Severity on the PTSD Checklist in VA's National Rollouts***

| | N | Pre Mean (*SD*) | Post Mean (*SD*) | Decrease | Standardized Pre-Post Mean Difference (*d*) |
|---|---|---|---|---|---|
| Prolonged Exposure | 1,354 | 63.0 (11.9) | 44.9 (16.8) | 18.1 | 1.21 |
| Cognitive Processing Therapy | 689 | 64.8 (10.6) | 44.5 (14.2) | 20.3 | 1.57 |

*Note*. PTSD severity was measured on the PTSD Checklist (PCL, Weathers et al., 1993). Using a CAPS difference of 10 points as a benchmark (Schnurr et al., 2001), a difference of 8 points would be considered to be the minimum indicating treatment response based on a regression of the PCL on CAPS scores (Monson et al., 2008).

In summary, the data comparing PE and CPT are inconclusive. There has been only one direct comparison. It was not sufficiently powered and the study population was exclusively female non-Veteran rape survivors. Findings from recent quantitative reviews suggest that CPT has a somewhat larger effect on PTSD severity scores, but methodological factors may explain the difference. Methodological factors also may account for the larger effect size observed for CPT than for PE in the VA rollouts. CSP #591 would resolve the ambiguity about the comparative effectiveness of PE and CPT by providing a definitive test between the treatments.

*2. Provider beliefs about PE and CPT*

Data from an ongoing study by Cook (including Co-Proponents Schnurr and Ruzek) suggest that VA clinicians prefer CPT over PE and believe it to be more effective than PE, even though there is no evidence to substantiate this belief. In the study, 201 providers from 38 VA residential PTSD treatment programs participated in a web-based survey about implementation of PE and CPT (Cook et al., 2012). Questions were derived from Greenhalgh et al.'s (2005) model of implementation that was based on Roger's (2003) classic model.

***Table I.3. Innovation Characteristics and Construct Ratings for PE and CPT in VA Clinicians***

| | PE | CPT |
|---|---|---|
| | *M* (*SD*) | *M* (*SD*) |
| Relative advantage* | 6.02 (1.40) | 6.65 (1.67) |
| Compatibility* | 7.11 (1.61) | 7.65 (1.62) |
| Complexity | 7.33 (1.49) | 7.24 (1.77) |
| Trialability* | 6.21 (1.47) | 7.26 (1.39) |
| Observability | 7.39 (1.34) | 7.54 (1.35) |
| Potential for reinvention* | 7.08 (1.56) | 8.00 (1.46) |
| Risk* | 5.95 (2.76) | 5.19 (1.76) |
| Task issues* | 6.87 (1.94) | 7.31 (1.63) |
| Nature of knowledge | 7.95 (1.41) | 8.01 (1.37) |
| Augmentation-technical support* | 7.63 (1.42) | 7.91 (1.55) |
| Skills* | 2.66 (2.02) | 3.08 (1.61) |
| Dedicated time and resources* | 6.60 (1.68) | 7.57 (1.71) |
| Incentives and mandates* | 6.53 (2.28) | 7.53 (2.06) |

*p < .05

The data reported in **Table I.3** show that CPT was rated more positively than PE on a range of innovation characteristics, including *relative advantage, compatibility, trialability*, *potential for reinvention, task issues,* and *augmentation-technical support*, and lower than PE on *perceived risk*). Consistent with the pattern noted for innovation characteristics, participants reported significantly higher skill with CPT than with PE, and both more *dedicated time and resources* and *incentives and mandates* for CPT than for PE.

Other findings suggest that some clinicians incorrectly believe that exposure therapies like PE are difficult for patients to tolerate and that these treatments can lead to symptom worsening and increased dropout, despite evidence to the contrary (Feeny et al., 2003; van Minnen et al., 2010). For example, van Minnen (2012) found that 82% of participants rated PE as an acceptable treatment and 50% preferred it over EMDR or medication. Also, there is no difference in dropout across studies of exposure therapy, cognitive therapy, other types of CBT, and EMDR (Hembree et al., 2003). Reliable data comparing PE and CPT could help to conclusively address unfounded beliefs and biases and encourage therapists to present treatment options to patients in a neutral manner that supports patient choice.

*3. Patient preferences*

There are no published data on patients' preferences for PE versus CPT. Results of a recent randomized clinical trial of a PTSD decision aid that are currently being prepared for publication, 34% of the participants wanted CPT after viewing the decision aid, versus 3.8% who wanted PE and 17.7% who wanted medication; 43.9% had no preference and 1.5% preferred EMDR (Watts & Schnurr, 2012). The low percentage of participants who wanted PE is surprising in light of van Minnen's (2012) finding that 50% of participants preferred PE. However, the difference could be explained by the fact that CPT or other types of cognitive therapy were not given as an option in van Minnen's study. Regardless of the reason, providing patients with reliable information about the comparative effectiveness could help them make informed choices about their care.

*4. Scientific and practical issues*

Given the limited evidence about the comparative effectiveness of treatments for PTSD, the proposed study would generate information that would be relevant not only to VA but also to DoD and the broader scientific community. Although there is no specific reason to indicate that Resick et al.'s (2002) results would not generalize to men and to other types of trauma

survivors, the applicability of the findings beyond female civilian rape survivors would be strengthened by a comparison in a more heterogeneous sample.

A comparison of PE to CPT has significant scientific relevance because each treatment reflects a different theoretical model of the etiology of PTSD. Prolonged Exposure is based on the Emotional Processing Theory of anxiety disorders and their treatment (Foa & Kozak, 1986) and its extension to explain the natural recovery after a traumatic experience, the maintenance of chronic PTSD, and treatment of the disorder (Foa & Cahill, 2001). Emotional Processing Theory proposes that PTSD can be conceptualized as a specific emotional structure that is characterized by two erroneous basic negative perceptions: the world is entirely dangerous and the PTSD sufferer is entirely incompetent. These perceptions are common in the immediate aftermath of a traumatic event, but are maintained by avoiding thinking about the traumatic event, which prevents processing of the event, and avoiding situations and objects that are distressing, which maintains the perception about the world as entirely dangerous and the self as entirely incompetent. According to the theory, to reduce PTSD symptoms, trauma memory must be activated and information that is incompatible with the basic erroneous perception must be incorporated in the trauma memory. This is accomplished by confronting the trauma through revisiting the traumatic memory in imagination and recounting it and processing it (to enhance organization of the traumatic memory and correct misconception about it) as well as in vivo exposure to distressing (but actually safe) stimuli which disconfirm that misconception that the world is entirely dangerous. Both kinds of exposure help disconfirm the perception of oneself as incompetent and unable to cope with stress.

According to the model of Cognitive Processing Therapy (Resick et al., 2002), PTSD develops because trauma survivors distort their beliefs about themselves and the world in an attempt to protect themselves from future trauma. They also tend to blame themselves or non-perpetrating others in order to maintain a belief in a just world ("I must have done something wrong, for this outcome to have occurred"). Treatment begins by focusing on distorted beliefs such as denial and self-blame and then shifts to distorted beliefs about oneself and the world ("No one can be trusted"). During treatment, patients are taught through Socratic questioning and daily worksheets to challenge their beliefs and assumptions. As beliefs become less distorted, patients generate more balanced self-statements for practice and PTSD symptoms lessen. Patients also write detailed accounts of the most traumatic incident(s) that they read to themselves and to the therapists in order to experience their natural emotions emanating from the event rather that those generated by erroneous beliefs.

If one treatment is found to be superior, this can further the development of understanding the etiology of PTSD and also may lead to enhanced prevention efforts, as well as refinement of existing treatments. There are also practical considerations. The standard PE protocol consists of 9-12 1.5-hour sessions, whereas the standard CPT protocol consists of 12 1-hr sessions. More sessions can be added to either treatment to achieve desired outcomes. However, the length of CPT sessions is easier to accommodate in VA, where mental health treatment sessions last 1 hour or less. CPT can also be implemented in group settings. In contrast, an important advantage of PE is that it can be used to treat other anxiety disorders such as simple and social phobia, panic disorder, and obsessive-compulsive disorder. Thus, PE offers a versatile approach that can be used to treat a wide range of patients.

In addition to knowing how PE and CPT compare overall, there is a similar need for information about the relative benefits for subgroups of patients. The study that compared PE and CPT (Resick et al., 2002) offers little guidance. The homogeneity of the sample in terms of gender and trauma type prevented the investigators from looking at the potential differences related to these variables. Subsequent analyses from this study (Rizvi et al., 2009) examined age, education, intelligence, depression, anger, and general (non-trauma) guilt as predictors of treatment outcome in PE and CPT. The investigators found evidence of differential symptom response to treatment for age only. Among younger women, those who received CPT had greater improvements in PTSD than those who received PE, whereas among older women, those who received PE had greater improvements. The investigators also looked at dropout, another important outcome, and found that higher baseline anger was related to dropout from PE, but not from CPT.

There is not enough evidence about predictors of differential treatment response in PTSD to justify powering a study to perform subgroup analysis, e.g., to examine whether men and women differ in response to PE and CPT. However, a study conducted in a large heterogeneous sample of male and female Veterans would permit exploratory analyses of predictors of response to PE and CPT. The information obtained would guide future research about what works for whom, a key goal of comparative effectiveness research and a necessary ingredient in delivering optimal, Veteran-centered care.

### D. Importance of the Proposed Research to VA

Of the almost 5.4 million Veterans who used VA care in FY 2011, 8.9% had a diagnosis of PTSD, including 8.7% of men and 11.6% of women. Prevalence is even higher in returning

Veterans who use VA care: almost 1 in 4 OEF/OIF/OND Veterans seeking VA care has PTSD (VA Northeast Program Evaluation Center, 2012). Prevalence is also high in Veterans of other cohorts (Fontana & Rosenheck, 2008), including those who have experienced military sexual trauma (Kimerling et al., 2008) and mild traumatic brain injury (Hoge et al., 2008). Furthermore, the costs associated with disability compensation for PTSD have increased substantially since the wars in Iraq and Afghanistan. In FY 2011, PTSD was the 3rd most prevalent service-connected disability, with 501,280 Veterans receiving some level of disability compensation for PTSD (Veterans Benefits Administration, 2012).

VA has a vested interest in understanding the relative effectiveness of PE and CPT (see **Section XVII** for a letter of support from Dr. Antonette Zess, Chief Consultant for Mental Health Services). Both treatments are recommended at the highest level in the VA/DoD PTSD Practice Guideline (Departments of Defense and Veterans Affairs, 2010). According to the Uniform Mental Health Services Handbook, VA is required to make these treatments available to Veterans seeking PTSD care. PE and CPT are being disseminated nationally across the VA system in order to enhance the availability of evidence-based treatments to Veterans with PTSD (Karlin et al., 2010). In FY 2011, VA instituted a new quality measure to enhance the likelihood that patients with PTSD will receive an evidence-based therapy like PE or CPT: specifically, the percentage of OEF/OIF/OND Veterans who engage in a new episode of care who receive at least 8 psychotherapy sessions in 14 weeks, which is a minimum frequency for treatments like PE and CPT. VA also has developed a national PTSD Mentoring Program for PTSD Program Administrators to help them manage their clinics to permit the delivery of these treatments. Every facility has an evidence-based therapy coordinator as well to facilitate training in evidence-based psychotherapy. In FY 2013 VA will be launching templates to facilitate note-writing in order to support delivery of PE and CPT.

Information about the relative effectiveness of PE and CPT is needed to help guide VA practice and policy. VA is emphasizing Veteran-centeredness and patient choice in healthcare options. Because there is no definitive information about how PE and CPT compare, Veterans have limited information on which to base their choice between the treatments, if both are available. Therapists are making their decisions about which treatments to offer based on their own experiences and beliefs. As described in **Section I.C.2**, these beliefs can be strong and erroneous. We confronted this problem directly when conducting CSP #494, which evaluated the effectiveness of PE for female Veterans and Soldiers (Schnurr et al., 2007). In the process of recruiting therapists we encountered opposition from some who felt that VA patients were too

complicated and fragile to do exposure therapy. Findings from CSP #494 indicated that PE was not only effective, but also safe and feasible. Yet findings from the ongoing study of the implementation of PE and CPT in VA residential programs described above show that VA providers hold more favorable beliefs about CPT (Cook et al., 2012). Although there is equipoise with respect to the potential effectiveness of the treatments, it is likely that patient choices are not sufficiently informed by evidence.

## E. Feasibility of a Cooperative Study within the VA

A multi-site study is required to attain the statistical power needed for a study that aims to compare two effective treatments and to examine factors that relate to differential treatment response. Power is a key consideration. It would not be possible to obtain sufficient power with data from a single site, or even from a few sites. In addition, the multiple sites enhance generalizability of findings and will help us obtain a more realistic effect size than we might from using just a few sites.

VA is uniquely positioned to conduct a study that would be extremely difficult at best to do in the civilian sector. The VA Cooperative Studies Program is able to efficiently support the number of sites needed. In addition, the national rollouts of PE and CPT have enhanced the ability to do large-scale psychotherapy research on these treatments. As of August 2012, approximately 4,600 VA therapists have been trained in one or both treatments. We will require participating sites to have at least 4 therapists who are proficient in PE and 4 who are proficient in CPT (i.e., they have been trained and undergone case consultation).

At present 75 sites meet this criterion, and more are expected to qualify as the rollouts continue. We have received letters of interest from 36 sites to date (see Volume 2 of the study protocol for letters). There is a high degree of enthusiasm at these sites for taking part in the trial. The locations, and the number of unique outpatients treated for PTSD at these locations in FY 2011, are listed in **Table I.4**. Thirteen of these sites are part of VA's Women's Health Practice-Based Research Network (PBRN), which has agreed to work with us to ensure enrollment of adequate numbers of women in the trial (see **Section XVII** for a letter of support from Dr. Susan Frayne). Five sites are newly approved CSP NODES sites, and 4 of these sites also are PBRN sites. We also will give consideration to sites that have a high number of enrolled OEF/OIF/OND Veterans to ensure adequate representation of this cohort.

***Table I.4. PTSD Outpatients in FY 2011 at Sites Expressing Interest in Participating in CSP #591***

| Site | Number of Patients | Site | Number of Patients |
|---|---|---|---|
| Alexandria, VA | 2133 | Long Beach, CA† | 4278 |
| Asheville, NC | 2845 | Madison, WI | 1650 |
| Atlanta, GA | 6948 | Miami, FL* | 4075 |
| Central CA/Fresno | 2060 | Minneapolis, MN*† | 3205 |
| Coatesville, PA* | 1236 | Montana HCS | 2152 |
| Chicago, IL/Hines*† | 3641 | Nebraska/Western Iowa | 2417 |
| Chicago, IL/Lovell | 1737 | New Orleans, LA* | 4284 |
| Chillicothe, OH | 1756 | Philadelphia, PA* | 5966 |
| Cincinnati, OH | 2973 | Phoenix, AZ | 6325 |
| Cleveland, OH | 5606 | Poplar Bluff, MO | 1917 |
| Columbia, MO | 2179 | Portland, OR*† | 5261 |
| Durham, NC* | 5448 | Salisbury, NC | 5976 |
| Eastern Colorado HCS | 8003 | St. Louis, MO* | 3234 |
| Eastern Kansas HCS* | 3471 | San Francisco, CA* | 3450 |
| Fayetteville, AR | 5633 | San Juan, PR | 2370 |
| Houston, TX*† | 7622 | VA Puget Sound, WA* | 8906 |
| Kansas City, MO | 3146 | VA Maine HCS | 3344 |
| Loma Linda, CA | 5491 | Western NY/Buffalo | 3247 |

*Site in the Women's Health Practice-Based Research Network; †Site in NODES program.

If we assume that only 5% of patients are enrolled, we find that a site with at least 1280 unique patients should be able to recruit 64 patients over a 2.5-year period. All of the potential sites except 1 (Coatesville, PA, which would have to enroll 5.2%) have a number of patients that would exceed this threshold. The remaining sites would have to enroll less than 1% to 3.9%.

Furthermore, our inclusion and exclusion criteria were designed to yield a maximally generalizable sample of patients for comparative effectiveness, replicating insofar as possible the actual patients with whom these treatments could be used (**Section IV.A**). The aim of the criteria is to promote participants' safety and their ability to engage in treatment. Inclusion requires that a person have PTSD and have telephone access or be able to come to VA for phone interview. A participant also must agree to study conditions: recording of assessments and treatment sessions and not receiving other treatment for PTSD (except medication and self-help groups) while receiving study treatment. In CSP #494, of 353 potential participants who met with staff to learn about the study, only 5 refused to agree to study conditions, none refused consent, and 28 simply did not continue, for a total of 9%. Exclusion criteria are nonrestrictive

and most are conditions that could change and allow future eligibility: significant cognitive impairment, suicidality, homicidality, and current (but not past) substance dependence, psychotic symptoms, and mania (including manic phase of bipolar disorder). In FY 2011, 11.8% of VA patients with PTSD had alcohol dependence (D. Kivlahan, personal communication, October 17, 2012), less than 1% of OEF/OIF/OND VA users diagnosed with PTSD also sustained severe TBI (N. Sayer, personal communication, October 17, 2012), and only 1.20% (7,361 of 611,357) of Veterans with a PTSD diagnosis had a reported FY11 non-fatal suicide attempt (J. Kemp, personal communication, October 17, 2012). Although we have been unable to find a data source that would permit us to apply our criteria to derive precise estimates of the number of eligible patients per site and the above data do not reflect all of our criteria, the prevalence of exclusionary rule-outs is not likely to impair feasibility of recruiting a sufficient number of eligible patients.

In reality, the number of potentially eligible Veterans is likely to be larger than indicated in **Table I.4.** The number of patients who received treatment for PTSD in the VA increased by 356,781 (249.4%) between 1997 and 2010, not only because of the wars in Iraq and Afghanistan but also due to increased number of Vietnam Veterans seeking PTSD care (Hermes et al., 2012). Since 2005, growth has increased at an annual rate of 14.8%, compared with 12.6% between 1997 and 2005. In FY11, 476,515 men and 38,002 women who received specialized mental health treatment in VA were seen for a diagnosis of PTSD (VA Northeast Program Evaluation Center, 2012). Approximately 93% used outpatient mental healthcare. The number of mental health visits by PTSD patients increased 1.6% annually from 1997 to 2005, and then jumped 19.6% annually from 2005 to 2010, when the average number of visits was 14.8 (Hermes et al., 2012). Thus, the growing prevalence of PTSD in users of VA healthcare supports the feasibility of the trial as well.

The feasibility of the trial is further enhanced by the fact that treating a patient on the study protocol could help a facility meet the new PTSD quality measure of delivering 8 sessions of psychotherapy in 14 weeks. Another important factor is how the ongoing dissemination initiatives will reduce startup time and overall costs. In our prior VA Cooperative Studies and in psychotherapy research more generally, the initial months of start-up include training therapists and providing expert supervision while they treat practice cases. Costs also are increased by the need to provide careful supervision as they gain increased proficiency while treating study cases. We can decrease the substantial costs of training and supervision by using therapists who have completed the required VA training in PE or CPT and received the case consultation

that is necessary for being specifically designated as a PE or CPT provider. Using trained therapists also decreases study duration by 6-9 months, along with the associated infrastructure costs for study management. We also have developed efficient and cost-effective methods of providing ongoing supervision in the dissemination initiatives. Use of these methods further enhances the feasibility and generalizability of the proposed project.

Controlled studies have demonstrated the effectiveness and acceptability of PE and CPT in male and female Veterans (Monson et al., 2006; Nacasch et al., 2011; Schnurr et al., 2007). Pilot studies have further demonstrated the effectiveness and acceptability in OEF/OIF/OND Veterans (Chard et al., 2010; Rauch et al., 2009; Yoder et al., 2012) and feasibility of treating Veterans with traumatic brain injury (TBI) (Chard et al., 2011). Thus, the treatments would be broadly applicable across a range of VA patients with PTSD.

Our record and experience further support the feasibility of the proposed study. The Principal Proponents and members of the Planning Committee have a demonstrated record of successfully conducting multisite psychotherapy research studies in VA, including 2 VA Cooperative Studies, CSP #420 (group therapy for male Vietnam Veterans; Schnurr et al., 2003) and CSP #494 (PE for female Veterans and active duty personnel; Schnurr et al., 2007). Drs. Foa and Resick are the developers of PE and CPT, respectively, and have conducted many of the most influential trials of PTSD treatment (e.g., Foa et al., 2005; Resick et al., 2002). In addition to his experience in conducting psychotherapy trials, Dr. Friedman brings experience in pharmacotherapy research and programmatic support from his leadership role in the PTSD Mentoring Program.

Members of the Planning Committee also designed the VA's national rollouts of the treatments we propose to compare. Co-Chairs Chard and Ruzek direct these rollouts and Dr. Eftekhari is the manager for the PE rollout. Dr. Tuerk is a PE expert and a trainer for the PE rollout. Furthermore, we have significant support from the Mental Health Services program office: Dr. Sonja Batten, Deputy Chief Consultant for Specialty Mental Health, and Dr. Brad Karlin, National Mental Health Director for Psychotherapy and Psychogeriatrics (who leads VA's Evidence-Based Psychotherapy program), have served as part of the Planning Committee and are committed to facilitating the success of the trial (see the letter of support in **Section XVII**). Dr. Jennifer Vasterling, a neuropsychologist and Co-Proponent of CSP #566 ("Neuropsychological and Mental Outcomes of Operation Iraqi Freedom (OIF): A Longitudinal

Cohort Study"), brings expertise in TBI and the use of conducting diagnostic assessment by telephone.

## F. Summary

Despite solid evidence that PE and CPT are effective treatments for PTSD in Veterans and non-Veterans, there is insufficient evidence about the relative effectiveness of these treatments. A comparative effectiveness trial of PE and CPT would be important from both a practical and a scientific standpoint, and have relevance within and outside VA:

- First, there is a pressing need to understand how the treatments compare with one another in order to assist VA leadership, clinicians, and Veterans in making informed choices about the delivery of PTSD care in VA. The attached letter from Dr. Antonette Zeiss, Chief Consultant in VA Mental Health Services, specifically describes how the study results would help to strengthen policy and practice of mental health care in VA. A unique advantage for this study is that there are administrative structures led by members of the study team—specifically the Evidence-Based Psychotherapy Program and the PTSD Mentoring Program—that exist to facilitate implementation of study findings. The findings would inform clinical practice outside VA as well.

- Second, there is a compelling scientific reason to compare the treatments. They are based on differing theories about the development of PTSD. A demonstration that one treatment is superior to the other would further scientific exploration by challenging theoretical accounts of etiology and treatment. Better evidence about etiology and underlying mechanisms would then have the potential for advancements in the prevention and treatment of PTSD. The Agency for Healthcare Research and Quality (2012) has recommended comparative effectiveness trials of effective PTSD treatments and the Institute of Medicine (2008) specifically noted the need for research on Veterans.

- And third, the available evidence is suggestive but not conclusive. With only one head-to-head comparison that was conducted in a relatively small and select sample of non-Veteran trauma survivors, it is not possible to draw reliable conclusions about the comparative effectiveness of PE and CPT. A large multi-site trial with men and women would substantially strengthen the inferences that could be drawn from the study and the study's impact on the field.

When designing the study, we considered the option of proposing an equivalence design given the limited evidence suggesting that the treatments differ.  We also considered proposing a traditional superiority design, hypothesizing that CPT is superior to PE given the 2012 AHRQ report and the rollout data favoring CPT.  However, because methodological factors may account for the apparent difference between PE and CPT, we decided to propose a traditional superiority design with a nondirectional hypothesis.  We believe the question it allows us to ask—***is one treatment better than the other***?—is the most important and most appropriate given the available evidence.

By designing a study large enough to detect a small difference ($d$ = .25), we are willing to risk the possibility that the true difference between PE and CPT is smaller.  If so, the difference would have little scientific or practical value.  In contrast, finding that one treatment is superior would enhance understanding of both etiology and treatment and yield information that is actionable.  If CPT is more effective than PE, the fact that CPT involves shorter sessions could encourage more efficient use of resources.  If PE is more effective, this would provide important justification for both clinicians and patients about the relative benefits of a treatment that they might otherwise avoid because of the intense trauma focus elements.  Regardless of which treatment is better, patients would have more information to help them make an informed decision about which treatment to choose and VA would have stronger evidence to help make care Veteran-centered.

# II. SPECIFIC OBJECTIVES

This study is designed to provide information for patients, clinicians, administrators, and policymakers about the comparative effectiveness of treatments for PTSD.

## A. Primary Objective

The primary objective is to compare the effectiveness of Prolonged Exposure and Cognitive Processing Therapy for reducing the severity of PTSD symptoms.

## B. Secondary Objective

The secondary objective is to compare the effectiveness of Prolonged Exposure and Cognitive Processing Therapy for reducing the severity of comorbid mental health problems and service utilization and improving functioning and quality of life.

## C. Tertiary Objective

The tertiary objective is to examine whether discrepancy between patient preferences and treatment assignment reduces the effectiveness of each treatment.

## D. Exploratory Analyses

Exploratory analyses will examine whether demographic and clinical characteristics predict differential response to PE and CPT. Although data are insufficient to justify a much larger study to address the question of which treatment works for which patients, these exploratory analyses can generate findings to inform future hypothesis-driven research.

Exploratory analyses also will characterize amount and quality of treatment and examine how treatment dose (e.g., number of sessions, total number of hours), treatment engagement (homework), and treatment fidelity (therapist adherence and competence) relate to outcomes within and between treatments.

## III. SUMMARY OF STUDY DESIGN

The study will be a prospective randomized clinical trial with blinded assessment. The population will be male and female Veterans with PTSD due to any traumatic military event. Patients who are eligible and agree to participate in the study will be randomly assigned to receive Prolonged Exposure or Cognitive Processing Therapy. Prior to randomization, patients will be stratified by hospital.

The primary outcome is improvement in PTSD symptom severity as measured by change on the Clinician-Administered PTSD Scale after treatment (Weathers et al., 2001). The outcome measure will be determined from regular follow-up visits of the participants, which will occur prior to, at the middle and at the end of treatment and then 3 and 6 months later (**Table II.1**).

***Table II.1. Participant Flow Through the Trial***

| Week | Event |
|---|---|
| * | (Initial entry into Mental Health program for self-referrals) |
| * | Screening phase 1: Referral source questioned regarding inclusion and exclusion criteria |
| * | Screening phase 2: First meeting with potential participant to explain the study protocol, obtain consent, gather information about demographic background and MST and TBI history, screen for cognitive impairment, and administer baseline questionnaires |
| * | Screening phase 3: Participant is interviewed by telephone to establish inclusion and exclusion diagnoses and obtain treatment preference |
| * | Randomization assigned |
| * | Scheduling of initial session with therapist |
| 1 | Treatment begins |
| 6 | Midtreatment Assessment** |
| 12 | Treatment ends** |
| 13 | Posttreatment assessment** |
| 24 | Interim assessment (3 months)** |
| 36 | Final assessment (6 months)** |

** Enrollment typically will take about 1 month. **The schedule is presented for a standardized participant. Actual time to complete treatment may vary as described in **Section VIII.B.2** and **Section VIII.B.3**.*

### A. Study Population

Participants will be male and female Veterans with PTSD due to any military event. Inclusion and Exclusion Criteria are described in **Section IV.A**.

**B. Study Treatments**

The study treatments are Prolonged Exposure and Cognitive Processing Therapy. The treatments are described in **Section V**.

**C. Outcome Measures**

The primary outcome is improvement in PTSD symptom severity on the Clinician-Administered PTSD Scale. A complete description of the measures is provided in **Section VII** and the assessment procedures are described in **Section VIII.A**.

**D. Sample Size**

In order to detect a standardized mean difference in improvement in PTSD symptom severity of $d = .25$, a sample size of 900 randomized participants provides 90% power to detect a difference between arms using the linear mixed effects model with a two-sided $\alpha = .05$. A detailed description on sample size and power considerations is given in **Section X**.

**E. Study Monitoring**

The intake rate and operational aspects of this study will be monitored continuously by the Study Chair and Study Biostatistician. Participating medical centers will continue in the study only if adequate patient intake is maintained, as defined by the Data Monitoring Committee (DMC) at its first meeting prior to the start of the study. A complete description of interim study monitoring is given in **Sections E and XII.D**.

## IV. PATIENT POPULATION AND PATIENT RECRUITMENT

### A. Inclusion and Exclusion Criteria

Participants will be male and female Veterans with PTSD due to any military event. Selection criteria will follow those used in CSP #494 and other trials of PE and CPT as well as the PE and CPT rollouts to ensure feasibility and participant safety.

**Inclusion criteria**: Current PTSD and symptom severity of the DSM-V equivalent of 45 or higher on the DSM-IV Clinician- Administered PTSD Scale (Weathers et al., 2001); agreement to not receive psychotherapy for PTSD during study treatment; to allow digital recording of phone interviews and therapy; and regular access to a telephone (or agreement to come to the VA for centrally conducted telephone interviews for participants who do not have telephone access). Medication for PTSD and other mental or physical conditions, psychotherapy for other problems, brief visits with an existing therapist, substance abuse treatment, and self-help groups will be allowed. Individuals who are taking psychoactive medication must be on a stable regimen (no dose or medication change) for 30 days prior to study entry. Monitoring for psychoactive medications will occur at the Phase 1 Screen and immediately prior to the Phase 2 Screen (when participants give informed consent and formally enter the study). Site personnel will document that they checked each participant’s psychoactive medication regimen at study entry in the medical record or the patient binder.

**Exclusion criteria**: Substance dependence not in remission for at least 1 month; current psychotic symptoms or mania (or manic phase of bipolar disorder); significant current suicidal or homicidal ideation that includes a specific plan; or moderate to severe cognitive impairment defined as 1 *SD* below age-graded norms on the Montreal Cognitive Assessment [MoCA] (Nasreddine et al., 2005).

Patients who are currently incarcerated are not eligible to participate in the study. If a patient becomes incarcerated during the course of their participation in the study, the patient will be withdrawn immediately. Patients may be re-consented and re-enroll in the study if they are still eligible to participate upon their release.

### B. Recruitment

Participants will be recruited from VA specialty and general mental health clinics, primary care, deployment health clinics, Vet Centers, and the community. The sites will be encouraged to use

a variety of recruitment strategies: presentations by the Local Site Investigator (LSI) or Study Coordinator (SC) to clinical personnel at the referral programs to remind them about the study; attendance at clinical team meetings; follow-up with individual clinicians; networking with Veterans groups likely to yield potential participants; advertising; and direct contacts of Veterans who have not opted out after receipt of an introductory letter. We also have engaged the support of the Women's Health Practice-Based Research Network to enhance the enrollment of women in the trial (see the attached letter from Dr. Susan Frayne, the Network's Director, and see attached "Women's Enhanced Recruitment Process [WERP]" memo) and expect support from NODES directors at participating sites as well. Under WERP, special efforts will be made to enhance recruitment of women at the CSP NODES sites that are co-located with PBRN sites and, as much as possible, at all CERV-PTSD sites.. In particular, CSP NODES sites that are co-located with PBRN sites will have supplemental SC time that they will invest in recruitment of women to this study, applying IRB-approved recruitment procedures. Women are an extreme minority group among Veterans in VA and are likely to be under-represented without such active outreach efforts. Achieving enhanced representation of women is important so as to allow for exploratory analyses of whether gender predicts differential response to the PTSD treatments studied here.

At each site, potential participants will be referred to the Site Coordinator by clinical staff in VA, Vet Center, or non-VA settings. Potential participants also may contact the SC directly. Alternatively, after receipt of an introductory letter, potential participants who do not opt out may be contacted directly by the SC.

*1. Clinician referrals*

The SC or LSI will provide clinicians at their site with information about inclusion and exclusion criteria to assist them in making appropriate referrals. To make a referral, a clinician will need sufficient information about the potential participant to answer the Phase I screening questions about probable PTSD DSM-5 diagnoses. In cases where the referring clinician does not have sufficient information for Phase 1 screening, he or she must obtain it from the potential participant if the recruitment process is to continue.

A clinician may mention the study to a patient who the clinician feels would be an appropriate referral, or a patient may ask the clinician about the study after having heard about it through advertising. Clinicians at the sites will be provided with brief information sheets about the study

in order to facilitate this process. If the patient agrees to be referred, the clinician would then contact the SC, who would then conduct the Phase 1 screening with the clinician.

At times, informal “offline” discussions between the SC/LSI and a referring clinician to determine eligibility of potential study participants may help facilitate the referral process.  For example, a clinician might first call the SC/LSI to discuss whether approaching a particular Veteran would be appropriate for the study if the Veteran had PTSD due to military trauma experienced outside a warzone (yes). These discussions may be more frequent at the beginning of the study than at the end, by which time clinicians are likely to have a better sense of patients who are appropriate for referral.

*2. Self-referrals*

Potential participants who contact the Site Coordinator directly will be given information that will enable them to decide whether they want to be considered for the study, i.e., purpose of the study, the two treatment conditions, use of random assignment, time commitment required for both treatment and assessment, and schedule of payments they would receive for participation. Potential participants who are currently in treatment will be asked to discuss their participation with their current therapist, and if this therapist is not a staff member at the site, the potential participant will be referred to the clinical program at the site for an initial intake into that program.  Once the potential participant has received an intake interview, the recruitment process would proceed as described in the clinician referral procedures in section **IV.B.1**.

*3. SC-initiated direct contacts to women Veterans who appear to be potentially eligible for the study*

An additional approach which may be used as needed to boost recruitment of women Veterans is for research staff to directly contact patients.  This will involve several steps.

*Step 1: Identify potentially eligible women Veterans in the sampling frame.*  One approach to developing the sampling frame  may be for the SC to review clinic lists (available locally) of potentially eligible women Veterans with upcoming clinic appointments in primary care or mental health clinics who are potentially eligible for the study (based upon evidence of PTSD in the local clinical databases, or based upon clinician input).  Another potential approach to developing the sampling frame may be for research staff in the CSPCC to pull lists of potentially eligible women Veterans (women Veterans with evidence of PTSD in VINCI databases),

including their name, SSN, and mailing address. CSPCC research staff would then move this "sampling frame" for particular participating site to a site-specific folder on the secure server at CSPCC; each Site Coordinator can access his/her own site-specific folder on the CSPCC server.

*Step 2: Refine the list via chart review.* The SC may review the chart of women Veterans on the sampling frame list, to eliminate those who clearly do not meet study criteria. The SC may also consult with a clinician who is familiar with the patient, if additional information is needed to supplement the chart review.

*Step 3: Mail introductory letter.* For women Veterans who appear potentially eligible for the study based upon Steps 1 and 2, the SC will mail a letter notifying the Veterans that they may be contacted about possible participation in a research study. A call-back number and a self-addressed stamped envelope and an opt-out card will be included in the mailing. Opt-in instructions will also be included, for those who want to self-refer to the study.

*Step 4: Directly contact Veterans who do not opt out.* For women Veterans who do not opt out by telephone or mail within 14 days of sending the introductory letter, the SC will make two attempts to contact the Veteran by telephone to orient her to the study (see Telephone Script). If the potential participant is interested, then (a) the SC will invite her to attend a mental health intake visit to assess potential eligibility for the study, or, (b) if, after Phase 1 referral conversation with the patient's clinician, the SC has already determined that she is appropriate for referral to the study, then the SC will invite her directly to a Phase 2 visit (see IV.C.1).

*4. SC-initiated direct contacts to all women patients at a facility*

The prior section describes the process of sending a letter to women Veterans who appear to have PTSD based upon chart review. An additional recruitment option that sites may use as needed is to apply the above procedures (opt-out letter followed by a phone call) to *all* women Veteran patients at the facility, as a mechanism for identifying women with PTSD symptoms whose PTSD is not identified by searching clinical records or VINCI databases. A letter will be sent (in batches) to all women at the facility. Women with evidence of PTSD (as described in the prior section) who do not opt out will be called by research staff to ascertain their interest in the study. Women without evidence of PTSD in VINCI/on chart review will be contacted only if they actively opt-in; this has the advantage of being an approach that will allow for identification of some women with PTSD symptoms not currently being addressed in VA.

### *5. Women's Enhanced Recruitment Process (WERP)*

To ensure women are well represented in the study sample, the recruitment processes described above will take into consideration approaches that maximize recruitment of women. For example, Women's Health providers may be among the clinicians approached about this study (with the support of the Women's Health Practice-Based Research Network [PBRN] Site Lead, at those locations that are part of the PBRN); presentations may be given to groups of women Veterans, among other networking venues; advertisements may be posted in areas frequented by women Veterans, among other locations; and women Veterans may be directly called by the SC after sending an introductory letter, as described above.

### *6. Efforts to support recruitment*

The National Study Coordinator at the Chair's office will contact each Site Coordinator by phone every other week to support recruitment efforts. The National Study Coordinator also will conduct monthly conference calls with all Site Coordinators in order to discuss issues related to all aspects of study management. Our experience in CSP #420 and CSP #494 was that a combination of individual and group contact helped to identify and resolve problems and to share lessons learned across sites. In addition, the Chair's office also will place a notice about the study on the National Center for PTSD website and will explore other centralized mechanisms of generating referrals.

## C. Screening and Consent

The diagnostic assessments done at study entry will provide final determination of a participant's eligibility for the study by confirming the inclusion and exclusion psychiatric diagnoses. We propose to use screening and consent processes the study team has employed successfully in CSP #420 and CSP #494. All participants, including self-referrals, will enter the study through referral by a mental health clinician or other qualified clinician at the participating site. Participants who are not currently receiving treatment at the site will first undergo an intake at the site's mental health program so that a clinician there can refer them. This strategy helps provide continuity of care for potential participants who do not enter the study, or who terminate from treatment early or need additional treatment during the study.

Screening information will be obtained in three phases, structured so as to minimize both participant burden and cost to the study due to extensive assessment of ineligible participants.

*1. Phase 1*

In the first phase of screening, the Site Coordinator will consult the referring clinician in order to establish a provisional PTSD diagnosis and other inclusion and exclusion criteria. In CSP #420 and CSP #494 this strategy resulted in a highly efficient screening process. For example, in CSP #494, 43 of the 396 patients who were discussed with a referral source were ruled out at this phase. Of the 353 patients who met with study staff, 320 were screened and 284 were randomized—71.7% of those discussed and 88.8% of those screened.

The referral clinician also would be asked to agree at this time to not treat the participant for PTSD while the participant is receiving study treatment, and would be reminded that treatment for other problems and brief check-ins are possible. The Site Coordinator will then schedule potential participants who are eligible based on the information provided by the referral source for Phase 2 screening. The SC will contact the potential participants initially by phone (up to 5 times) and then by mail with a letter.

*2. Phase 2*

During the second phase of screening, the Site Coordinator will review the Informed Consent form with a potential participant to explain the study in more detail. Participants will be fully informed of the nature and extent of study participation, the objectives of the study, and the two treatments to which they will be randomly assigned. In order to enhance participants' understanding of the treatments, the Site Coordinator will also read a brief standardized description of each treatment and will provide a written description of each treatment for participants to take home. Participants will be informed of the fee payment structure that they will receive for completing assessments they undergo as part of the project. Site Coordinators will be trained to make sure that all participants comprehend the nature of the study and the wording of the consent form and will provide a copy of the form for participants to take home.

After obtaining consent, the Site Coordinator will administer demographic questions, the VA TBI and MST screens, suicide and homicide screening, and the MoCA (Nasreddine et al., 2005). If responses on the suicide or homicide screen indicate significant risk, the Veteran is not eligible for the study, and the Site Coordinator will contact a project-affiliated mental health clinician who will conduct suicide risk assessment guided by VA's Suicide Risk Assessment Guide Reference Manual and VA Assessment Pocket Card (2007). If on the basis of the risk assessment the Veteran is found to be at risk for suicide or homicide and in need of

intervention, the clinician will develop a safety plan agreed upon with the Veteran. All safety plans for suicidal patients will be created according to the standard procedures described in the VA manual, “Safety Plan Treatment Manual to Reduce Suicide Risk: Veteran Version” (Stanley & Brown, 2008). Safety plans for homicidal patients will be developed following local and national directives. All plans may include support from the VA, family contacts and friends, and other people the patient trusts. Safety plans will also incorporate the VA Veterans Crisis Line phone number: 1-800-273-TALK (8255) as a support outlet.

In addition, the Site Coordinator will contact the referring VA clinician to ensure that patient and clinician work together to address any clinical need. Any safety plan that has been created by the project-affiliated clinician will be communicated to the referring VA clinician.

The MoCA was initially designed to detect mild cognitive impairment. However, new findings based on a larger, more diverse normative sample have shown that (a) the initially recommended cutpoint of 26 is too sensitive, especially for the goal of excluding only cases of more extensive cognitive impairment and (b) there are meaningful differences across age groups not taken into account by the original cutpoint of 26 (Rossetti et al., 2012). Based on these new findings, we propose defining impairment as 1 SD below age-graded norms as follows: younger than 35 years (< 21); 35-44 years (< 20); and 45 years or older (< 18).

Potential participants who do not rule out based on the MoCA or suicide or homicide screens and who agree to continue will then complete baseline questionnaires and be scheduled for a screening interview with one of the centralized Assessors in Phase 3.

*3. Phase 3*

In the third phase of screening, Masters- or Doctoral-level Assessors at the Boston VA Medical Center will contact potential participants by telephone to assess PTSD and other psychiatric diagnoses, employing procedures currently in use in CSP #566 and Project VALOR, a registry study of OEF/OIF/OND Veterans (**described in Section VIII.A.2**).

Because the study would begin after the adoption of DSM-5 criteria (scheduled for finalization in January 2013 and official release in May 2013), we will use versions of the CAPS (Weathers et al., 2001) and the Structured Clinical Interview for DSM-IV (SCID; Spitzer et al., 1995) that have been updated to reflect changes to the diagnostic criteria for PTSD and other disorders. The phone interview also will include measures of treatment preference. The phone interviews are estimated to require 2-2.5 hours, and may be broken into two sessions, as needed.

Participants meeting eligibility criteria will then be assigned to treatment. As indicated in **Section VI.A**, the Site Coordinator will use the system established by the Palo Alto CSPCC to obtain the participant's treatment assignment. The Site Coordinator will contact the participant by phone to provide information about the participant's treatment assignment and schedule the participant's initial therapy appointment. If more than 30 days elapses between the Phase 3 appointment and the first treatment session, the CAPS will be re-administered to allow for an accurate baseline measurement. The SC will attempt to contact the participant by phone 5 times and finally with a letter requesting a response of interest in the study. If there is stil no response it will be assumed that the potential participant is no longer interested in the study.

## V. TREATMENT

### A. Prolonged Exposure

***Prolonged Exposure*** (Foa et al., 2007) is a manualized, 90-minute, 8-15 week treatment program based on emotional processing theory (Foa & Kozak, 1986; Foa & Cahill, 2001), which posits that anxiety disorders, including PTSD, reflect pathological fear structures in which emotional and cognitive associations among different elements do not accurately represent reality and renders the individual dysfunctional and distressed. PE is designed to correct erroneous connections in the targeted memory structure. PTSD sufferers typically experience two key pathological emotional response sets and related cognitions: "The world is an utterly dangerous place," and "I am completely incompetent and unable to cope with stress." In this study, the 12-session protocol will be followed, but participants who improve more rapidly may finish in 10 sessions and those who improve more slowly may have up to 2 additional sessions to continue working on exposure. The procedure for determining number of sessions is described in **Section VIII.B.3**.

The central components of PE are in vivo and imaginal exposure. In vivo exposure consists of gradually and systematically having patients approach trauma-related situations, places, and people that elicit distress and have been avoided. Repeated exposure to these stimuli disconfirms the negative, unrealistic expectations of harm and the individual experiences a reduction in the associated fear response. Between sessions homework of in vivo exposure consists of systematically confronting trauma-related situations that are avoided and to remain in the situation until distress reduces by half. Imaginal exposure involves repeated revisiting of the memory in imagination and recounting aloud the traumatic event(s) in detail, while vividly imagining the event(s) and paying specific attention to emotions and thoughts that occurred *at*

the time of the event.  Typically, as patients move through the imaginal exposure process, and distress reduces, they can focus on increasingly specific details of the event and integrate “new” information that had been overlooked due to longstanding habitual and willful avoidance of the memory.  The revising and recounting of the traumatic event is followed by processing the revisiting experience. Processing provides an opportunity for patients to examine their beliefs related to the trauma memory, integrate the meaning of newly available information, and to gain a new perspective on the trauma, as well as to realize that they can handle successfully engaging with the traumatic memory rather than avoiding thinking about it.  Similar to in vivo exposure, repeated and prolonged imaginal exposure provides experience that disconfirms negative erroneous cognitions (e.g., if I engage with the traumatic memory rather than avoid it I will “fall apart”) and reduces emotional distress associated with confronting the memory. Treatment sessions are audio-recorded and patients are asked to listen to their recounting of the trauma daily.  Participants will give their consent to be audio-recorded during therapy for the purpose of imaginal exposure homework exercises .  As this audio-recording is not analyzed data, but simply part of the usual and manualized PE treatment protocol, this audio-recording will not be beind VA firewall.  Participants may borrow a tape recorder from their PE provider, or use their own audio recording device (e.g. cell phone) if they prefer.  Psychoeducation and controlled breathing exercises play a secondary role in PE.

Psychoeducation comprises a discussion about what maintains PTSD, common reactions to trauma, and reasons why facing fears in a safe environment is therapeutic. Controlled breathing training is designed to impede the person’s sympathetic nervous system response by slowing down oxygen intake, it is a tool used early on in the treatment process to encourage self efficacy and mastery of symptoms.

Session 1 begins with an overview of the treatment program and a general rationale for how exposure works.  The therapist gathers information using a standardized interview, focusing on the patient’s symptoms, details of the trauma, history of previous trauma, and social and occupational functioning.  Breathing retraining is introduced and the patient practices slow and uniform breathing techniques.  Homework is daily breathing exercises, auditing a recording of the session, and reviewing a “Rationale for Treatment” handout.

Session 2 focuses on education, treatment planning, and development of the in vivo exposure hierarchy.  The therapist provides an explanation of PTSD, discusses common reactions to trauma, discusses a rationale for the treatment, and provides a description of each treatment

component.  The use of Subjective Units of Distress (SUDS) ratings is explained.  A list of avoided phobic situations is compiled, and an exposure hierarchy is developed.  Homework includes practicing breathing exercises daily, listening to the recording of the session at least once, reviewing the list of avoided situations and adding items to the hierarchy if appropriate, reviewing a “Common Reactions to Trauma” handout daily, and in vivo exposure.

Session 3 reviews the rationale for PE and introduces prolonged imaginal exposure.  The patient is to be guided through 60 minutes of imaginal reliving of the trauma.  The patient is instructed to relive the trauma as vividly as possible, and to recount it aloud in the present tense.  This procedure is repeated until the exposure period is expended.  If the patient exhibits reluctance to engage fully in reliving the trauma, the therapist reiterates the rationale for the treatment and reminds the patient to confront the feared image gradually.  SUDS ratings are obtained every 5 minutes and vividness, every 10 minutes.  After the imaginal reliving, the patient is encouraged to talk about reactions to reliving the trauma and to discuss related thoughts and remembered details.  The patient will learn to identify, evaluate, and modify disturbing thoughts and feelings, and develop more realistic beliefs about personal coping ability with stress and the dangerousness of the world.  In vivo exercises are selected from the hierarchy and discussed for practice between sessions.  Daily homework is to use the recording to relive the trauma, in vivo exposure, breathing practice, and to review the session recording.

Sessions 4 and up to the session before termination focus on imaginal in vivo exposure for 60 minutes, followed by a discussion of the thoughts and feelings about the reliving.  During imaginal exposure the therapist asks specific questions to clarify the patient's thoughts, feelings, and physical reactions while reliving the trauma to facilitate confrontation with fear-evoking cues.  The parts of the scenario that are the most anxiety-producing to the patient are identified, and emphasized in repeated exposure.  After each exposure the therapist and patient discuss reactions to the reliving, as in previous sessions.  In vivo exercises are selected from the hierarchy for homework practice.  As for previous sessions, daily homework is to use the recording to relive the trauma, in vivo exposures selected during session, breathing practice, and to review the session recording.

Last Session (Termination):  Imaginal exposure lasts 30 minutes.  The therapist and patient review treatment progress and discuss applications of treatment principles to daily life. This discussion will address the potential for temporary increases in PTSD symptoms, and how these can be managed.

## B. Cognitive Processing Therapy

**Cognitive Processing Therapy** (Resick et al., 2010) consists of cognitive therapy and a written trauma narrative. Patients are taught to challenge their beliefs through Socratic questioning and the use of daily worksheets. The initial focus is on beliefs such as denial and self-blame, and then shifts to overgeneralized beliefs about self and the world. Patients process their trauma directly by writing a narrative of their traumatic event(s) that they read to themselves and to therapists. The typical protocol consists of 12 1-hr sessions. In this study, the 12-session protocol will be followed, but participants who improve more rapidly may finish in 10 sessions and those who improve more slowly may receive up to 2 additional sessions to continue working on stuck points with challenging beliefs worksheets. The procedure for determining number of sessions is described in **Section VIII.B.3**.

Session 1 consists of education about symptoms of PTSD and the recovery model of PTSD from a cognitive theory perspective. The therapist and patient determine the worst trauma (if different from that which was the focus of the CAPS) and the patient gives a brief description of the event. The therapist gives an overview of the therapy, explains what a stuck point is (a distorted cognition about one’s role in the trauma, or implications about oneself, the world or other people) and gives the patient handouts to read as well as a stuck point log. Finally, the therapist explains the first practice assignment, to write an impact statement about the patient’s beliefs about why the worst traumatic event occurred and how it has affected beliefs about self and others, particularly in the areas of safety, trust, power/control, esteem and intimacy.

Session 2: The patient reads the impact statement (or constructs it orally if the patient didn’t do it). The therapist and patient move stuck points from the impact statement to the stuck point log as the therapist conducts gentle Socratic questioning about any erroneous self or other blame and has the patient label emotions they experience when they think the distorted thought. The next assignment is introduced: the ABC sheets on which the patient records events, thoughts, and feelings. The therapist uses examples from the impact statement to illustrate how the worksheets are used and how different thoughts lead to different emotions. Patients are assigned to complete one worksheet each day and at least one must be on the worst traumatic event.

Session 3: The therapist and patient review the ABC worksheets the patient completed and may complete others during the session. Gentle corrections may be made if the patient has difficulty identifying his/her thoughts or emotions and helping the patient construct stuck points

into a more easily challenged format (e.g., “if only I had done X, the event would not have happened”). The therapist uses Socratic questions to examine the evidence supporting or refuting the patient’s beliefs. The next assignment is for the patient to handwrite an account, at home, of the worst traumatic event. The patient is instructed to draw a line on the paper and stop briefly if he/she becomes very emotional. Also unlike PE, the account is written in the past tense and they may take more than one occasion to complete the assignment. The patient is asked to read the account to him or herself every day until the next session but this typically takes only a few minutes. They are asked to continue completing ABC sheets each day about items on the stuck point log or stuck points that emerge through the written account.

Sessions 4 and 5: The patient reads the account at the beginning of the session. The therapist does not interrupt the reading and sits quietly if the patient experiences emotions in the process of reading the account. When the patient has reoriented to the therapist, the therapist asks about emotions experienced at home while writing or reading the account, and in session and then asks about any parts of the event that may have been omitted or glossed over. The rest of the session is spent doing Socratic dialogue regarding any erroneous self or other blame, determining, who if anyone had intent to do harm (and therefore is the actual cause of the event). Hindsight bias, outcome based reasoning and mislabeling of guilt are all corrected through Socratic dialogue. The Just World myth may be reexplored. The patient is assigned to write the account a second time and notice any changes in emotions or beliefs. The patient continues to complete ABC worksheets. At session 5 the patient reads the new account and Socratic dialogue continues. In the last third of the session, the therapist introduces the challenging questions sheet in which the patient writes one of his/her stuck points at the top of the page and asks him/herself a series of questions about the validity of the statement. At this point the therapy begins to shift to teach the patient to challenge his/her own thinking. The patient may be assigned to continue reading the account or to write an account about another trauma.

Session 6: The therapist and patient review the challenging questions worksheets and any further work on accounts. The problematic thinking patterns worksheet is introduced and examples are generated. In this worksheet, the patient is asked to notice stuck points and everyday thoughts that are tendencies for them to engage in such as jumping to conclusions, mindreading, emotional reasoning, or all or none thinking.

Sessions 7-12: After reviewing the problematic thinking patterns, the final worksheet and first of five themes is introduced. The Challenging Beliefs Worksheet compiles the content of all the previous worksheets and adds a final column in which the patient generates a more balanced and fact-based statement. The patient is asked to rate how much he or she believes this new statement and how much they now believe the old stuck point. The patient is also asked to name and rate the intensity of emotions before and after completing the worksheet. One theme is explored each week in addition to the person's individual stuck points. Each of the themes, safety, trust, power/control, esteem and intimacy can be self or other oriented. Three other assignments are given in the last few sessions in addition to reading handouts on the themes and completing worksheets. Patients are asked to practice giving and receiving compliments to reengage with other people and to challenge their core beliefs and are asked to do at least one pleasurable or worthwhile activity for themselves each day. This latter assignment is to help the patient reestablish what they used to enjoy doing and to build self-worth. Either of these assignments may trigger stuck points that can then be challenged. Behavioral activation can serve as relapse prevention for depression as well. The final assignment before session 12 is to rewrite the impact statement about how the patient now thinks about the causes of the trauma.

Session 12 begins with a review of the intimacy work sheets and then the patient reads the new impact statement. The therapist reads the original statement and they compare the differences. They also notice areas that the patient needs to continue to practice working on stuck points and review the whole course of therapy.

## VI. TREATMENT ASSIGNMENT

### A. Randomization

After the Site Coordinator has gone through the checklist to verify that the participant has signed the informed consent form, met all the enrollment criteria, and completed the baseline assessments, he/she can use the system established by the Palo Alto CSPCC to randomize the participant. Participants will be randomly assigned in a 1:1 ratio to receiving PE or CPT. Participant randomization will be based on permuted blocks within each study center. After the participant is randomized, the Site Coordinator will obtain the treatment assignment and complete the case report form (CRF) on randomization.

## B. Blinding

Using the standard double-blinding procedures employed in medication research is not feasible or desirable in psychotherapy research. Therapists need to be aware of which intervention they are delivering, and participants need to know as well. Instead, the gold standard in psychotherapy trials is to use blinded assessment, as described in **Section VIII.A.4**. Study staff at each site will not be involved in the collection of the primary outcome data. Use of centralized phone assessment for the primary outcome enhances blinding because assessors are not physically located where participants are receiving treatment, which offers an additional layer of protection from accidental unblinding. For secondary outcomes, the Site Coordinator will collect participant self-reported questionnaires by providing folders containing the questionnaire measures to participants and then collecting these folders from participants after completion (**Section VIII.A.4**).

## VII. MEASURES

The measurement protocol follows closely the protocols used in CSP #420 and #494. An important difference in CSP #591 is that new diagnostic criteria for PTSD and other disorders were implemented between submission of the proposal and the study's beginning. Members of the study team were active participants in the revision of the PTSD criteria for DSM-5. Dr. Friedman chaired the workgroup, with Drs. Schnurr and Resick as participants. A DSM-IV to DSM-5 crosswalk study found that the new criteria applied well to both Veteran and non-Veteran samples.

The National Center for PTSD has revised and validated two key instruments, the CAPS and the PCL, according to the new diagnostic criteria in DSM-5. Similarly, the PTSD Diagnostic Scale (PDS; Foa et al., 1997) is being revised and validated for DSM-5. Below we describe the existing measures but will use the new DSM-5 measures, noting important exceptions where needed. Criteria for other disorders may change as well, so we propose to use the DSM-5 criteria for these disorders also.

### A. Screening and Eligibility

The Clinician-Administered PTSD Scale (Weathers et al., 2001) is a clinician-administered interview that measures diagnostic criteria for PTSD according to the American Psychological Association's Diagnostic and Statistical Manual. The CAPS has excellent reliability and validity and is the gold standard for PTSD treatment research (Weathers et al., 2001). Each of the 17 DSM-IV symptoms is rated on a 0-4 (low to high) scale for both frequency and intensity. In DSM-5 these two rating scales will be combined into a single severity scale, which substantially reduces the amount of time needed to administer the CAPS. This will facilitate telephone administration.

In DSM-IV, symptoms of PTSD are categorized into 3 clusters: 5 reexperiencing symptoms (B Cluster), 7 avoidance and numbing symptoms (C Cluster); and 5 hyperarousal symptoms (D cluster). In order to receive a diagnosis of PTSD (American Psychiatric Association, 1994), a person must be exposed to a traumatic event; have 1 or more B symptoms, 3 or more C symptoms, and 2 or more D symptoms; experience significant distress or impairment because of the symptoms; and have symptoms for at least 30 days. Symptom severity is computed by summing the totals for the 17 items.

In CSP #494, diagnosis required that a participant meet DSM-IV diagnostic criteria (frequency ≥ 1 and intensity ≥ 2 for a symptom to be counted) and have a minimum level of severity (overall severity ≥ 45). In DSM-5, the avoidance and numbing symptoms have been split into separate clusters and additional symptoms have been added. In CSP #591 we will use the new diagnostic criteria along with a severity score for entry that corresponds to a score of 45 on the current DSM-IV version. Dr. Marx, who will oversee the telephone assessment procedures of the study, is currently conducting a validation study (in collaboration with CAPS developer Dr. Frank Weathers) that will allow us to identify the score that corresponds to a score of 45 on the CAPS for DSM-IV.

The CAPS includes a lifetime trauma checklist (the Life Events Checklist, or LEC) and questions about stressor exposure, which will be used to ensure that participants meet the DSM-IV criterion of stressor exposure that is required for diagnosis. The trauma checklist also will provide descriptive information about participants at study entry. In addition, we will supplement the interviewer questions about Criterion A exposure to permit categorization of trauma types according to the categories developed by Stein et al. (2012): life threat to self, life threat to others, aftermath of violence, traumatic loss, moral injury by self, and moral injury by others.

The Structured Clinical Interview for DSM-IV (SCID), patient version (First et al., 2002), assesses Axis I psychiatric disorders. It will be used during screening to establish exclusion diagnoses; the data also will be used for sample description and for exploratory analysis to examine non-PTSD psychiatric disorders as predictors of treatment outcome. It is arguably the most widely used clinician-administered instrument for assessing psychiatric disorder. That study estimated reliability to be good using a conservative method in which different clinicians independently interview and rate the same participant; the overall weighted kappa was .61 for current diagnosis and .68 for lifetime diagnosis. We will use the SCID for DSM-5. As in CSP #420 and CSP #494, we will administer the modules for mood disorders, anxiety disorders (other than PTSD), and substance abuse, along with the psychotic screen.

Demographic information will be collected during screening, along with information about cognitive impairment on the Montreal Cognitive Assessment (Nasreddine et al., 2005). We will screen for history of traumatic brain injury and exposure to military sexual trauma using the standard VA screening measures for these constructs and for suicidality using two items drawn from the University of Washington Suicide Risk/Distress Assessment Protocol (Linehan, Comtois, & Ward-Ciesielski, 2012); because there are no comparable measures of homicidality,

we will modify the two suicide items to assess homicidality as well. The suicide screening questions will be administered by the interview assessor at posttreatment and follow-up interviews. Treatment preferences will be measured as in a recent study by Feeny et al. (2010), in which participants are asked for a counterbalanced forced choice among treatment options and then rate their confidence in their choice rating. We also will measure expectations and treatment credibility for both treatments as in CSP #494 using the Expectancy of Therapeutic Outcome Scale (Borkovec & Nau, 1972).

### B. Primary Outcome

Improvement in the CAPS PTSD severity score from baseline will serve as the primary outcome.

### C. Secondary Outcomes

As in CSP #494, we will use the CAPS to compute additional measures of clinical outcomes: response (defined as at least 10-point improvement in severity), loss of diagnosis (response plus no longer meeting DSM symptom criteria), and remission (loss of diagnosis plus the DSM-5 score that corresponds to a DSM-IV severity score < 20).

The PTSD Checklist (Weathers et al., 1993) is a brief questionnaire measure of PTSD symptom severity that is widely used within and outside VA. In both PE and CPT, therapists administer the PCL at the beginning of each session and review progress with participants during the treatment. The scale consists of the 17 DSM-IV symptoms rated on a 1-5 scale of how much that symptom bothered the individual in the prior month. The PCL is an efficient measure of PTSD symptoms and has high sensitivity and specificity for a CAPS PTSD diagnosis. It has excellent psychometric properties (Bliese et al., 2008) and is sensitive to treatment-related change (Monson et al., 2008). At the beginning of the VA rollouts, therapists administered the Beck Depression Inventory-II (BDI-II; Beck et al., 1996) in PE and CPT to monitor depression symptoms. Therapists now administer the Patient Health Questionnaire-9 (PHQ-9; Kroenke et al., 2001) instead. The PHQ-9 has been well-validated as a depression outcome measure (e.g., Lowe et al., 2004).

However, because the unblinded therapists will administer the PCL and PHQ-9 as part of treatment, it is important to use different measures of PTSD and depression symptoms as outcomes. Having confronted a similar issue in a recent RCT of collaborative care for Veterans with PTSD in which the treatment protocol required care managers to administer the PCL as

part of care management (Schnurr et al., 2013), we turned to the PTSD Diagnostic Scale (Foa et al., 1997) to measure PTSD. The PDS consists of the 17 DSM-IV symptoms of PTSD rated on a 0-3 scale of how often the symptom has occurred in the past month. Like the PCL, it has excellent psychometric properties (Foa et al., 1997; Griffin et al., 2004) and has been used in many trials of treatments for PTSD (e.g., Ehlers et al., 2005; Foa et al., 2005; Foa et al., 1999; Resick et al., 2008). Thus, we will use the DSM-5 version of the both the PDS and PCL.

For similar reasons, we will use the BDI-II to measure depression during blinded assessment. It has excellent psychometric properties and is widely used in PTSD treatment research (e.g., Foa et al., 2005; Monson et al., 2012; Resick et al., 2002; Schnurr et al., 2007). Other secondary outcomes include several brief questionnaires. Anger symptoms will be assessed with the State Anger subscale of the State-Trait Anger Inventory (Spielberger, 1988). We will assess substance abuse using the Short Inventory of Problems-Revised (SIP-R; Kiluk et al., 2012) and selected items from the Brief Addition Monitor (Cacciola et al., in press)—number of heavy drinking days and number of drug use days)—chosen because of evidence that measures like this are clinically sensitive endpoints (Falik et al., 2010). We also will assess functioning (WHODAS-II; World Health Organization, 2000), quality of life (WHOQOL-BREF; World Health Organization, 1996), and satisfaction (CSQ; Attkisson & Zwick, 1982).

We will use a combination of self-report and VA administrative data to measure service utilization. VA and Non-VA utilization will be measured using a brief questionnaire adapted from one developed for the HSR&D-funded Barriers to Care Study (Vogt, ongoing). We will use VA administrative databases to obtain information about VA-funded utilization. We will collect information on individual PE and CPT sessions (based on the new session templates to be launched in FY 2013), outpatient prescriptions, other outpatient services, and inpatient care within all settings (acute, rehabilitation, and long-term care). We will use the Medical SAS datasets (OPC for outpatient care, PTF for inpatient care) to capture all VA inpatient and outpatient utilization. To obtain detailed information about non-study treatment before, during, and after treatment, we will develop a questionnaire to measure types of psychosocial treatment and medication use by adapting questionnaires used in CSP #494. At all NODES sites, and at other study sites wishing to participate in collecting this information, we will additionally administer Veteran Feedback Forms (VFFs) which are adapted from an Ohio State University instrument [Miser], and intended to collect information about how participants heard about the study, participants' motivations for joining the study, and participants' feedback on study recruitment processes ("VFF Baseline", administered at Phase 2), and feedback regarding

experience with being a research participant (“VFF Follow-up”, administered at the in-person 6-month Post-treatment Assessment). Feedback responses will inform efforts to optimize Veteran-centric recruitment in future studies.

## VIII. STUDY PROCEDURES

We relied heavily on our experiences in CSP #420 and CSP #494 when designing the study procedures, including the selection and timing of measures, methods of assessment, delivery of treatment, and methods to ensure and monitor protocol fidelity. Modifications have been made when necessary, e.g., to accommodate the centralized telephone assessment and the possibility of additional treatment sessions, and also to enhance compatibility with other trials, e.g., assessing the primary outcome at mid-treatment.

We also utilized current experience in CSP #566 with procedures to facilitate telephone assessments. We have recruited Dr. Brian Marx from the Behavioral Sciences Division of the National Center for PTSD in Boston, who oversees the telephone assessment in CSP #566, to help us design this part of the procedure for CSP #591. He has agreed to oversee telephone assessment in CSP #591. The Behavioral Science Division is an ideal site to provide leadership. The Division led the development of the CAPS and PCL, and is internationally recognized for leadership in the assessment of PTSD. The division houses a number of doctoral-level staff members who are experienced in administering the CAPS and SCID in research protocols.

### A. Assessment

#### *1. Schedule*

The schedule of assessments is presented in **Table VIII.1**. The primary outcome, the CAPS, will be administered via telephone before, during, and after treatment, and at 3- and 6-month follow-up. In addition, the PCL and the PHQ-9 will be administered weekly during the course of therapy as part of the treatment protocol. Participants' expectations and preference will be measured at the beginning of treatment and treatment satisfaction will be collected at the end of treatment only. Other secondary outcomes will be collected before and after treatment and at 3- and 6-month follow-up, with the exception of utilization, which will be measured before and after treatment and at 6-month follow-up.

***Table VIII.1. Assessment Schedule***

| Measure | Baseline | During treatment | Post-treatment | 3-months | 6-months |
|---|---|---|---|---|---|
| Clinician-Administered PTSD Scale | X | X (session #6) | X | X | X |
| Posttraumatic Diagnostic Scale | X | | X | X | X |
| Beck Depression Inventory-II | X | | X | X | X |

***Table VIII.1. Assessment Schedule***

| Measure | Baseline | During treatment | Post-treatment | 3-months | 6-months |
|---|---|---|---|---|---|
| Spielberger State Anger Inventory | X | | X | X | X |
| Brief Addiction Monitor (2 items) | X | | X | X | X |
| Short Inventory of Problems-Revised | X | | X | X | X |
| WHO-DAS-II | X | | X | X | X |
| WHOQOL-BREF | X | | X | X | X |
| Client Satisfaction Questionnaire | | | X | | |
| Treatment preference | X | | | | |
| Expectancy of Therapeutic Outcome | | X (session #1) | | | |
| Utilization | X (6 months prior) | | X (time since baseline) | | X (time since posttreatment) |
| Structured Clinical Interview for DSM-5 | X | | | | |
| MoCA | X | | | | |
| Demographic information | X | | | | |
| VA TBI Screen | X | | | | |
| VA MST Screen | X | | | | |
| Suicide Screen | X | | X | X | X |
| Homicide Screen | X | | | | |
| Life Events Checklist | X | | | | |
| PTSD Checklist | | X (weekly) | | | |
| Patient Health Questionnaire-9 | | X (weekly) | | | |
| Veteran Feedback Form (VFF Baseline) (at NODES and selected other sites) | X | | | | |
| Veteran Feedback Form (VFF Follow-up) (at NODES and selected other sites) | | | | | X |

### *2. Telephone Assessment*

Independent Assessors located at the Boston VA Medical Center will conduct the third phase of screening and perform all outcome assessments by telephone. Boston will conduct and oversee all aspects of the clinician-administered interviews performed in the study, including supervision and fidelity monitoring. Boston was chosen as the site given the extensive experience of Dr. Brian Marx and staff there in conducting telephone interviews in CSP #566 and Project VALOR, a registry study of OEF/OIF/OND Veterans. The independent assessors are members of the research team and are VA or WOC employees.

Although we considered conducting in-person interviews, we elected to conduct telephone interviews for several reasons. First, being able to complete the interviews by phone is convenient for participants because it prevents them from having to make an additional trip to the VA in order to be interviewed. Second, centralized assessment enhances quality control by reducing site-level variation in interview fidelity and quality. Third, the psychometric quality and

acceptability to research participants of psychiatric phone interviews are now well-established in Veteran (Aziz et al., 2004; Magruder et al., 2005; Schnurr et al., 2002) and non-Veteran (e.g., Rohde et al., 1997; Sartor et al., 2012; Shalev et al., 2012) samples. For example, Rohde et al. (1997) found that the inter-rater reliability of phone interviews was excellent (kappa = .96 for major depressive disorder and .87 for anxiety disorders), exceeding the .80 benchmark for excellent reliability. Magruder et al. (2005), using the CAPS with a sample of Veterans seeking VA care, found 100% agreement across interviewers. Fourth, because we are using separate therapists to administer PE and CPT, centralized phone assessment assures that the person who is collecting the primary outcome (the CAPS) will not see the therapist and participant together, inadvertently breaking the blind. In summary, the phone interviews will provide a valid method of assessing mental disorder constructs, yet be considerably more cost-effective and more convenient for participants than in-person interviews.

The Assessors will introduce the telephone interview portion of the study to the participant informing the participant about the content of the interview and then reminding them that the interview is being recorded. We will digitally record telephone assessments for the purposes of checking interviewer reliability, following procedures used in CSP #566.

All computers used for audio recordings will be VA-issued. Desktop computers will be networked within the VA system and password-protected. Laptops will be VA-issued, encrypted, password protected, and have VPN capabilities. Recorded files for CSP #591 will be saved directly to a secured drive behind the VA firewall. As an additional security measure, recorded files will be password protected.

### *3. Procedures to Enhance Completion of Assessment Protocols*

A number of procedures will be used to minimize the likelihood that participants will fail to complete the schedule of assessments. Detailed contact information will be obtained at study entry to facilitate follow-up, even for participants who move. This information will be updated at subsequent assessments. During treatment, the PCL and PHQ-9 questionnaires will be administered prior to a treatment session. Interview assessments that include the primary outcome will be conducted in telephone appointments scheduled for this purpose. Scheduling will occur by phone when possible. Participants who do not have telephones will be contacted by mail and asked to call the Site Coordinator to make an appointment to come in to the VA Medical Center for the telephone interview or to make arrangements to use a phone elsewhere. Five contact attempts including a final letter will be made before a participant is considered to be

unreachable at that time point. Participants who fail to participate in a scheduled assessment will be contacted by phone (or mail, when necessary) for rescheduling. After two missed appointments without explanation, a participant will be considered to have missed that assessment interval.

*4. Blinding*

Assessors located at the Boston VA Medical Center who conduct the interviews will be blind to a participant's treatment condition. Although the use of centralized telephone assessment can help to minimize unblinding that may occur at the sites (e.g., by seeing a participant come out of a given therapist's office), at each interview, the Assessor will remind the participant to not reveal the treatment condition to which the participant has been assigned. After each follow-up assessment, Assessors will indicate on the CRF if they become unblinded to a participant's treatment assignment.

Because Site Coordinators will deliver information about treatment assignment to participants, we have taken different precautions to ensure the validity of the secondary questionnaire assessments that will be collected by the Coordinators. The Site Coordinators will provide folders containing the questionnaire measures to participants and then collect these folders from participants after completion. Site Coordinators also will remind participants to not discuss their treatment assignment during the visit. The Site Coordinators will remain available to answer participant's questions but will not stay in the room with participants during questionnaire completion in order to minimize contact with participants while the questionnaires are being completed. However, Site Coordinators will check forms for completeness before a participant leaves so that any missed items can be completed. It is preferred that the secondary questionnaire assessments be completed by study participants in-person. However, it will be acceptable for Site Coordinators to send and collect secondary questionnaire assessments by mail for participants who are not able to attend an in-person visit (e.g., if the participant moved away after completion of treatment). If an assessment needs to be completed by mail, the Site Coordinator should contact the participant by phone and inform him/her that the assessment will be mailed. If the Site Coordinator finds, upon receiving the mailed assessments, that the participant endorses moderate or severe suicidal ideation, the Site Coordinator will inform the Local Site Investigator or a study clinician. The clinician will follow the procedures outlined in the CSP 591 high-risk protocol, and appropriate actions may include transferring the participant to the Veterans Crisis Line with the VA Warm Transfer Protocol (2-800-273-TALK, PRESS 1).

*5. Reliability*

All SCID and CAPS interviews will be digitally recorded.  Approximately one hundred SCIDs and 200 CAPS (sampled equally from each of the 5 assessment periods) will be randomly selected in an ongoing way in order to monitor and maintain the reliability of the interview process.  An Assessment Adherence Monitor, a doctoral-level clinical psychologist at the Behavioral Science Division of the National Center for PTSD, will be employed to specifically conduct reliability assessment under the supervision of Dr. Marx.  In order to maintain reliability, the Monitor will provide feedback to Assessors during biweekly supervision sessions that will continue throughout the study period.

Training for the Assessors will be standardized and systematically conducted by Dr. Marx.  All Assessors will have been required to have undergone formal training in the administration of the SCID and CAPS, although training will be standardized nonetheless.  Assessors will conduct practice interviews during training at the study's kickoff meeting and then again upon return to their performance site.  They will continue to conduct practice interviews and receive feedback from the Assessment Adherence Monitor until the Monitor judges them to be calibrated to a standard of administration consonant with the intentions of the developers of the respective interviews.

*6. Compensation*

**Table VIII.2** shows the estimated payment schedule.  Assessments will be compensated at a payment schedule designed to maximize retention by reflecting the additional effort that is needed to continue to participate in assessments after study treatment has been completed. Participants may find the continued assessment burdensome, so we propose to increase the 3- and 6-month assessments by modest amounts in relation to the additional burden.  The maximum payment will be $410.  The questionnaire assessment during treatment is extremely brief and will not be compensated because it is part of the standard of care.  Payment for screening and baseline assessment will be graduated. Participants who rule out of screening based on the suicide screen or the MoCA will be paid $30.  Those who are eligible to continue and complete the baseline questionnaires will receive an additional $20.  Participants will be paid $50 for completing the phone interview.  Participants will also be reimbursed for travel over 50 miles.

***Table VIII.2.  Estimated Participant Payment Schedule for Study Assessments***

| Assessment | Payment ($) |
|---|---|
| Screening/baseline/interview | 30/20/50 |
| Mid-treatment | 50 |
| Posttreatment | 75 |
| 3-month follow-up | 85 |
| 6-month follow-up | 100 |

## B. Treatment

### *1. Assignment*

Participants will be randomly assigned to treatment following the completion of baseline assessment measures that establish their eligibility for the trial.

### *2. Delivery*

Both study treatments, which are summarized in **Section V**, will be delivered according to standardized manuals.  We will use the manuals and the therapist and patient materials developed for the PE and CPT rollouts to administer the treatments.  The manual for each treatment is provided in Volume 2 of the study protocol.  Treatment will be delivered in an outpatient setting.

PE and CPT will be administered approximately weekly, although sessions may be held more or less frequently than once a week if needed, e.g., to accommodate a patient’s scheduling needs or to help a patient finish treatment within 20 weeks. For the sake of brevity, session frequency is described as “weekly” throughout this protocol.  The standard protocol for PE is 8-15 sessions (10 sessions were used for PE in CSP #494), whereas the standard protocol for CPT is 12. Given the flexibility in the number of sessions allowed according to the protocols in the VA rollouts, it would be difficult to constrain the total number of sessions to 12 in CPT and 10 in PE. Therefore, we propose to administer 12 sessions of each treatment as a standard “dose” but to allow participants who improve more rapidly to finish in 10 or 11 sessions and participants who have not attained adequate improvement by session 12 to have up to 2 additional sessions.

Data from the rollouts suggests that this range will address the needs of most Veterans who participate in the trial (**Table VIII.3**).  The average number of sessions among treatment completers is 11-12.  There are not fixed criteria for determining completion in either rollout, however.  In PE, completion is defined as attending at least 8 sessions.  In CPT, completion is

based on improvement as determined by clinical judgment and decrease in PCL scores, which is more comparable to what we propose to use.

***Table VIII.3. Number of Sessions in Veterans Completing Treatment in the VA Rollouts***

| | N | Mean (*SD*) | < 10 | 10-14 | > 14 |
|---|---|---|---|---|---|
| Prolonged Exposure | 1,723 | 11.09 (2.62) | 28.9% | 59.7% | 11.4% |
| Cognitive Processing Therapy | 689 | 11.83 (1.88) | 8.1% | 87.0% | 4.9% |

We chose to standardize the number of sessions at 12 in both treatments based on the available evidence showing that number of sessions is related to improvement in psychotherapy (up to a point, at which a longer number of sessions is a reflection of nonresponse to treatment) (e.g., Baldwin et al., 2009; Howard et al., 1986; Steenbarger, 1994). Almost none of the literature on the dose-response relationship in psychotherapy discusses session length. In fact, session length is hardly ever reported nor is it treated as a potentially influential variable. We found no discussions or evidence relevant to our decision to equate number of sessions and not total amount of treatment. We did find a small naturalistic study from the PTSD literature in which investigators were required to shorten exposure sessions from 90 minutes to 60 minutes during the course of a trial (van Minnen & Foa, 2006). The results suggested that total amount of treatment did not matter. There were no differences between the 60 and 90 minutes groups in PTSD and other outcomes. Although the study was not designed to examine session and was not powered to detect anything other than large differences, the similarity of findings in both groups was striking. However, we did not feel that this evidence was sufficient to support a formal change in the PE protocol from 90 to 60 minutes.

Our approach is an attempt to optimally balance standardization (to ensure internal validity) and flexibility (to enhance generalizability). Although there are inherent differences in duration of sessions in each treatment, we believe it is important to administer them in an ecologically valid way—that is, to not artificially equate the duration of sessions. Because this is a comparative effectiveness trial and not an efficacy study, we believe it is important to administer the treatments as they would be used in practice. We will perform sensitivity analyses to examine whether amount of treatment is differentially related to outcome.

### *3. Procedures for Early Completion and Additional Sessions*

The standard number of sessions of PE and CPT will be 12. However, participants may complete treatment with more or fewer sessions depending on their response to treatment.

Therefore, in addition to reporting number of sessions attended in each condition, we will also examine variability in early versus late completion in PE and CPT and explore how the variables relate to outcomes.

There are no standardized criteria for determining number of sessions for completion in the rollouts, so we are proposing procedures based on experience in the rollouts as well as studies that have used flexible dosing of manualized protocols for treating PTSD (Foa et al., 2005; Galovski et al., 2012; Levitt et al., 2007). Our aim is to optimize standardization and flexibility by ensuring that participants achieve substantial gains before terminating early (to ensure that they are stably improved) and at the same time not requiring extra sessions unless participants have failed to achieve an adequate response.

Participants who have experienced stable remission before completing 12 sessions many terminate early. Stable remission will be defined as 2 consecutive sessions in which the study participant reports (the DSM-5 equivalent of) a PCL score below 29 according to DSM-IV criteria. Beginning at session 8, participants who have a PCL below 29 for 2 consecutive sessions may terminate treatment early and receive the final session content at the subsequent session if they prefer to not complete all 12 sessions (i.e., in session 10 for someone remitted in sessions 8 and 9 and session 11 for someone remitted in sessions 9 and 10). A score of 29 was chosen because it is the lowest possible score on the PCL that could meet the DSM-IV symptom criteria for PTSD, and also corresponds to the definition of remission as a score below 20 on the CAPS (Weathers et al., 2001).

Participants whose PCL scores have not dropped below 50 by session 12 may receive up to 2 additional sessions depending on their preference for more treatment. We considered a range of scores between 40 and 50 as a threshold for determining whether participants would be offered more sessions. A score of 45 is the average posttreatment PCL among completers in the rollouts (**Section I.C.1, Table I.2**) and Monson et al.'s (2006) trial of CPT in Veterans; the posttreatment average in CSP #494 was 42 at the end of 10 sessions. By using 50, we are proposing a realistic threshold that is clinically meaningful and that many participants can attain without the need for additional treatment. According to data from the CPT rollout, of the 519 participants who completed 12 or more sessions (a standard dose), 60.3% were at or under 49 on the PCL at session 12. Note that this does not mean that 40% of participants in CSP #591 would require extra sessions, because it does not take into account participants who completed

in fewer than 12 sessions. What it does show is that the majority of participants who do not complete before session 12 will not require additional treatment.

It is common in psychotherapy trials for treatment to last more than the number of weekly sessions due to missed appointments, vacations, and other scheduling difficulties. In CSP #494, we attempted to have participants complete treatment within 16 weeks, although 20 weeks was allowed if there was consensus between the participant's therapist, supervisor, and study leadership overseeing that treatment condition. However, although the standard dose will be 12 sessions in CSP #591, we propose to attempt to have participants complete within 20 weeks because many participants are expected to finish in less than 12 sessions. If participants do not complete treatment within 20 weeks, we will offer them 1-2 additional sessions, including the termination session of their assigned treatment.

*4. Adjunctive Services and Attrition Prevention*

We will add a maximum of 2 additional sessions to be used in the event of significant participant crises or emergencies that present obstacles to study participation. These "stressor sessions" will be allocated within the PE and CPT protocols according to a procedure described by Galovski et al. (2012). We anticipate, however, that such sessions will be needed infrequently. In a recent study that incorporated this procedure in CPT (Galovski et al., 2012), only 13 out of 100 participants required such a session, at a rate of one per participant.

These sessions will be used to address significant psychosocial stressors or emergencies (such as death in the family, diagnosis of life-threatening illness, notice of home foreclosure, sudden loss of job with family needs dependent on income) that (a) occur during the course of treatment, (b) cannot be integrated into the ongoing PE or CPT treatment, and (c) are deemed likely to significantly interfere with a participant's ability to take part in PE or CPT if not addressed in depth.

If after a collaborative discussion with a participant a study therapist judges that a stressor session is necessary, the therapist will offer the participant the option of skipping one session of treatment in order to discuss and consider solutions for this stressor. Participants will be informed that a maximum of two such special sessions will be available to them as part of the study and that they can decide whether they need to use one of these extra sessions to discuss the stressor or continue with the PE or CPT protocol as usual.

The stressor session will focus on providing support, problem-solving the stressor situation, and/or applying PE- or CPT-related intervention components to the issue at hand. Therapists will be asked to ensure that they do not collude with avoidance by stopping the PE or CPT protocol, while also respecting the need to attend to emerging crises. These procedures are broadly consistent with usual practice in VA PTSD treatment. In the PE and CPT rollouts, therapists are instructed to help their patients deal with such obstacles and remain flexible in arranging additional help for their patients while still retaining components of the protocol that are useful in addressing the crisis.

Stressor sessions may occur current with, but outside of, study treatment. Alternatively, study treatment may be stopped temporarily if this is necessary for the participant to address the crisis. In the event that the therapist and participant decide that more than 2 sessions are needed to attend to a crisis, then the participant will be removed from study treatment, but allowed to resume therapy outside of the study if and when the participant chooses to do so.

*5. Additional Procedures to Address Suicide Risk*

Study therapists may learn of suicidal intent or ideation through the administration of the PHQ-9 at the beginning of each therapy session. If risk is indicated by the participant's responses to Item 1.9 or by the study therapist's observations during the course of normal interactions with the participant, a careful assessment guided by the study Suicide Assessment Procedures will be triggered.

If at any time a participant is found to be at risk for suicide and in need of intervention, the study therapist will develop a safety plan with the participant. All suicide safety plans will be created according to the standard procedures described in the VA manual, "Safety Plan Treatment Manual to Reduce Suicide Risk: Veteran Version" (Stanley & Brown, 2008). This plan may include support from the VA, family contacts and friends, and other people the participant trusts. The safety plan will also incorporate the VA Veterans Crisis Line phone number: 1-800-273-TALK (8255) as a support outlet. We will report these events as an AE if identified during the course of treatment. Failure of the participant to comply with the safety plan will require stopping study treatments and aggressively treating the suicidality.

During phone interviews at posttreatment and follow-up, the telephone assessor will screen for suicide risk using the protocol that is used for screening at enrollment. If responses indicate significant risk, the telephone assessor will conduct a risk assessment. If the assessment

indicates that the Veteran is at risk and in need of intervention, the assessor will actively facilitate referral to a Veteran Crisis Line counselor using warm telephone transfer for high or imminent risk Veterans. In addition, the assessor will contact the Site Coordinator and Local Site Investigator to inform them about the participant. The Site Coordinator will contact the referring VA clinician to ensure that participant and clinician work together to address any clinical need. Any safety plan that has been created by the project-affiliated clinician will be communicated to the referring VA clinician. The Assessor will contact the SI or referring clinician to refer moderate and low risk Veterans for further follow-up. The SI will be responsible for ensuring that necessary communications and procedures are followed for all study participants who are judged to be a risk during assessments.

All safety plans will be created according to the standard procedures described in the VA manual, "Safety Plan Treatment Manual to Reduce Suicide Risk: Veteran Version" (Stanley & Brown, 2008). This plan may include support from the VA, family contacts and friends, and other people the participant trusts. All safety plans will also incorporate the VA Veteran Crisis Line phone number: 1-800-273-TALK (8255) as a support outlet.

All suicide attempts and completions will be considered SAEs, and as such, will be reported to the study Executive Committee, Central IRB, and the DMC by the PI and Study Biostatistician, in addition to other standard VA reporting requirements. The DMC will monitor all SAEs regularly (at least every 6 months) throughout the study and assess potential for increased risks to participants. The DMC may also impose requirements for more frequent monitoring of SAEs.

*6. Additional Treatment*

As in CSP #420 and CSP #494, participants may receive some additional types of non-study treatment while receiving study therapy. They are allowed to stay on medication, attend self-help groups, and receive treatment for mental health problems other than PTSD. Participants who have a usual therapist also are allowed to see the therapist for brief supportive sessions if necessary. In addition, participants who develop problems requiring additional inpatient or outpatient treatment will be allowed to receive the additional treatment. They may stay enrolled in study treatment if this would be clinically appropriate, as determined by discussion involving the therapist, the therapist's study supervisor, and the Local Site Investigator. After completing treatment, participants will be allowed to resume any PTSD treatment that was discontinued or to seek additional treatment for PTSD. We will use a specifically detailed measure to assess

medication use during treatment that was developed for CSP #494, expanding the measure to capture psychotherapy.

Based on our prior experience, the majority of participants will be on some kind of medication and the clinicians prescribing the medication may wish to change drugs or dose while the participant is receiving study treatment.  In CSP #494, this happened more in the comparison group than in the PE group, we suspect, because clinicians were attempting to compensate for participants' assignment to the comparison group.  Our approach in both CSP #420 and CSP #494 was to try to discourage unnecessary medication changes but to respect participant preferences.  Medication changes were handled at the site level by individual clinicians, sometimes with the involvement of the LSI.

In CSP #591 we will offer consultation to study therapists or prescribing clinicians on best practices in medication management.  Dr. Friedman will perform this function by serving as the Medication Monitor, utilizing his experience providing similar consultation through VA's National PTSD Consultation Program.  The goal is not to prevent clinicians from doing what they feel is in the best interests of participants, but rather, to standardize insofar as possible the use of medications across participants and sites and discourage ineffective or potentially harmful prescribing practices.  We will use a blinded consult request form (modeled after the form currently used in the PTSD Consultation Program) so that Dr. Friedman can remain blind to a participant's assigned treatment condition during these consults.  Clinicians also will be reminded to not discuss participants' treatment assignment with Dr. Friedman during any discussions that follow.

### C. Discontinuation of Study Treatment

Experience with PE and CPT in the rollouts indicates that some participants will have temporary disruptions of study treatment due to other comorbid problems or life events, but that participants typically can come back into treatment after being stabilized.  However, participants will be discontinued from treatment if they show substantial worsening of PTSD, other symptoms, or functioning requiring lengthy hospitalization of if the worsening is due to treatment.  For intent-to-treat purposes, all participants, including those who are terminated from treatment early, will be followed at posttreatment and at 3 and 6 months.

Participants can be identified for discontinuation from treatment by their therapist or their non-study clinician (if they have one).  When a participant is identified as requiring discontinuation

for any of the reasons listed above, the individual who has made the recommendation for discontinuation will communicate with the other clinicians involved in the care of that participant, as well as with the LSI. The LSI will call a meeting as soon as possible to discuss this issue with all involved parties. If the discussion at this meeting confirms that the participant should be discontinued for any of the reasons, the LSI will communicate with the Master Therapist (Dr. Foa for PE and Dr. Resick for CPT) and Supervisor for that participant's assigned condition in order to obtain official study permission to discontinue treatment. Then, in conjunction with the appropriate clinicians, the PI will decide what method will be the most clinically sensitive mode for communicating the study team's decision.

### D. Withdrawals

Participants may withdraw from study treatment or from the study at any time. Those who withdraw from study treatment but wish to continue in the study will participate in study assessments according to the protocol. Those who withdraw consent will not be followed.

If a participant withdraws from the study (or is declared lost to follow-up), the participant may be re-consented and complete his or her participation in study treatment and/or study assessments.

### E. Post Follow-up Procedures

After completing study treatment and follow-up, participants will not be followed. They may continue any ongoing treatment or initiate new treatment for PTSD.

### F. Training and Supervision

Therapists will be chosen from among those who have completed the full training for either PE or CPT and are registered on VA rosters as providers of one of those therapies; this requires comprehensive review of training cases. Study training, aside from specific instruction regarding the protocol and documentation, will consist of a 1-day review of the therapy protocols to ensure that the therapists are implementing the therapy uniformly. To establish therapist proficiency, potential study therapists will be asked to submit two audio-recorded treatment sessions prior to selection. These sessions will be reviewed by a senior clinician who is approved to provide case consultation in the VA rollouts in order to establish adherence and competence with the treatments.

Therapy Supervisors, two for PE and two for CPT, will be selected to train and consult with

study therapists throughout the course of the study. The Therapy Supervisorswill provide case consultation in weekly group conference calls with no more than 8 therapists per call. The consultation will focus on problem-solving and other issues that arise in the course of delivering the study treatments. Review of audio recordings of therapy sessions will not be necessary for supervision because therapists will be required to have completed all VA provider training elements and review of audio recordings will be necessary to confirm therapists' proficiency before entry into the trial. However, therapists will be asked to audio-record every session for quality control. If there are concerns about a therapist or if a therapist requests more intensive consultation, one of the Master Therapists will review audio recordings and provide more specific feedback to address the problem.

### G. Therapy Fidelity Monitoring

Monitoring of therapist behavior and interventions in both treatment conditions is necessary to ensure treatment fidelity, i.e., that therapists are delivering the interventions specified in the manual and not using interventions that are not part of the treatment. Independent monitoring will provide a detailed assessment of adherence to the manuals and therapist competence. Using procedures developed in our prior studies (CSP #420 and #494), an independent Fidelity Monitor (a senior clinician who is not involved in training or consultation in the study), will rate two audio recordings from each study therapist for adherence and competence.

# IX. DATA COLLECTION AND MANAGEMENT

## A. Data Management

The Palo Alto CSPCC will be responsible for the management and the quality control of the data. After the study is approved, data forms will be finalized and field tested. An Operations Manual will be provided to the investigators to guide the operation and management of the study. A training session at the kickoff meeting is planned prior to the initiation of participant enrollment for all study personnel to assure uniformity in participant management and data collection procedures, and to train all study personnel in study procedures. The Study Coordinator at each medical center and the Independent Assessors at the Boston VA Medical Center will complete data forms on a daily basis and transmit them to the CSPCC. If paper forms are used, the original forms will be kept in the LSI's study files.

DataFax, a clinical trial data management system (by Clinical DataFax Systems, Inc.), will be used for data collection and management. DataFax allows for paper data form collection as well as electronic data capture (EDC). The Study Coordinators from the sites and the Independent Assessors will complete case report forms (CRFs) and fax them directly to the DataFax computer server, where data images of the CRFs are stored as files. The system uses an optical character recognition (OCR) paradigm to automatically process and store the information from the image as data into the study database. The original fax image is also stored. Data management staff at CSPCC will review each CRF by comparing the faxed image with the OCR data and ensure that the two match. If electronic CRFs are used, the Study Coordinators and the Independent Assessors will log into the web-based DataFax system and enter study data directly, rather than completing and sending a paper CRF.

In the case of the Veteran Feedback Forms, since Veterans will have the option of hand-writing responses to open-ended questions, the SC will scan the form and load it directly onto the CSPCC secure server, in a site-specific folder. The original scanned image will be stored, and manual data entry will be used for both quantitative and qualitative responses.

Data management staff at CSPCC will review CRFs for protocol adherence data consistency, and add data queries to items that fail these checks. Checks will be performed manually and programmatically. On a regular basis, data management staff will produce site-specific Quality Control reports that list all unresolved data queries. Data management staff will make the reports available to each site and work with the Study Coordinators and the Independent

Assessors to help them resolve queries. Queries will be resolved when the appropriate corrections to the CRF are made and data resent, or when an explanation is provided that allows for data management staff to resolve the query. All corrections and changes to the data will be reviewed by data management staff. In addition to the Quality Control report, CSPCC may generate and distribute targeted data edit reports on an as needed basis.

The Study Co-Chairs, the Study Coordinators and the Independent Assessors will receive periodic reports regarding the quality and quantity of data submitted to the CSPCC. Other quality control measures include periodic reports containing participant recruitment information and relevant medical data for review by the Study Co-Chairs. The CSPCC will also prepare summary reports for the Study Co-Chairs, the Data Monitoring Committee, and other monitoring groups of the data to track progress, and conduct final analyses of the study data.

Study reports will be generated using DataFax, SAS, Atlas.ti (for qualitative data analyses), and other tools (e.g., Microsoft Excel and Access). SAS and other statistical software packages will be used to conduct data analysis for the study. The CSPCC was using SAS Version 9.1 in October 2012 and will upgrade to newer versions once they are purchased and validated.

**B. Data Security**

The DataFax system is fully compliant with US Federal regulations regarding electronic data capture systems established by the Food and Drug Administration under 21 CFR 11. Data entered directly into the database provides the official clinical record for data collection. Source documentation is handled in the same manner as a paper-based system. All paper-based records will be kept in locked file cabinets at the sites and Boston VA Medical Center. The servers housing the study databases will be located at a secure VA facility and housed behind the VA firewall on VA-owned and -maintained servers. The system will be monitored to ensure that all applicable VA regulations and directives are strictly followed.

Access to the study data is restricted by the CSPCC to properly-credentialed research staff who have completed required VA security trainings. Only CSP-approved individuals (such as: staff at the study site, CSPCC, and CSP Clinical Research Pharmacy Coordinating Center (CSPCRPCC)) will have access to the personal health information (PHI) of study participants.

Research data will only be stored on secure VA servers within the VA firewall (and not on desktops or on University affiliate servers). The data will be coded with a unique study identifier for each participant and stored using that study identifier. Identifiable information will be

collected for participant tracking and safety purposes, and to collect health care usage data. Coded clinical data will be stored separately from the participant's name, contact information, and real SSN. Access to the cross-walk file linking the participant's identifiers and their study data will be restricted to the clinical site and to the study staff at the CSPCC.

In case of improper use or disclosure of study data, the facility's ISO and Privacy Officer, and the individual's direct supervisor will be notified immediately per VA Directive and Handbook 6500. Records will be maintained and destroyed per the VHA Records Control Schedule (RCS 10-1).

Quality control checks and clinical monitoring will enable the CSPCC to examine the database and the clinical sites to ensure data have not been improperly used or accessed. Audit trails and access logs compliant with 21 CFR part 11 will be checked routinely, and clinical monitors will provide continuing education on GCP and check clinical site operations for violations of data security policies and best practices.

### C. Proposed Data Collection Forms

Copies of the proposed data collection forms can be found in **Appendix C**.

## X. BIOSTATISTICAL CONSIDERATIONS

### A. Expected Treatment Effect

This study is a prospective randomized clinical trial aimed to compare the effectiveness of Prolonged Exposure (PE) to Cognitive Processing Therapy (CPT) for the treatment of PTSD in veterans. The primary outcome is the change of CAPS total score from baseline (pre-treatment) to the average in the six months post-treatment (measured at immediate post-treatment, 3 and 6 months follow-up visits). We chose to use the average in the six months post-treatment in the definition of primary outcome (versus using a single post-treatment timepoint) because we anticipate that improvement established during the course of treatment will be sustained in the 6 months after treatment for both PE and CPT. Incorporating multiple measurements from the same participant will also reduce the required sample size. The Planning Committee considered an effect size of 0.25 to be a clinically meaningful difference, where the effect size is defined as $\Delta\mu/\eta$, $\Delta\mu$ is the mean difference in the primary outcome between PE and CPT, and $\eta$ is the standard deviation of the change of CAPS total score from baseline to a specific post-treatment timepoint. By using CSP #494's estimated standard deviation $\eta$=19.6, the effect size of 0.25 translates to a $\Delta\mu$=4.9 points difference in the primary outcome. For simplicity, the sample size for this study is aimed to have 90% power to detect $\Delta\mu$=5 in the primary outcome.

Cohen (1988) defined 0.25 as a small effect. We have powered the study to detect this difference because both PE and CPT are effective treatments. It is implausible based on existing data to think that the true difference between them is much larger. Conversely, if we did not have adequate power to detect a difference as small as 0.25, then any failure to find a difference between treatments could be seen as inconclusive—which was the problem with the only study that directly compared the treatments (Resick et al., 2002). If the true difference between the effects of PE and CPT is less than 0.25, this would be clinically insignificant.

Although our sample size calculation is based on DSM-IV CAPS total score data observed in the CSP#494, the effect size of 0.25 is independent of DSM versions. In addition, a correlation coefficient among participants treated by the same therapist is a robust statistics for both location and scale change of CAPS total scores. We expect a negligible difference in inflation factors between DSM versions. Therefore, the sample size derived below is appropriate for CAPS on DSM-5 .

## B. Sample Size and Power Considerations

### *1. Planned primary analysis for the primary outcome*

Linear mixed effects models (SAS PROC MIXED) will be used to compare the primary outcome between the two treatment groups. The mixed effects model will include time, treatment, treatment by time interaction and site as fixed effects, and participant and therapist as random effects. Specifically let $Y_{ijk}$ denote the CAPS total score measured at timepoint $k$ for the $j^{th}$ participant treated by therapist $i$. The mixed effects model for the primary analysis is:

$$Y_{ijk} = \alpha + \beta_k + \gamma_{z(i)} + (\beta\gamma)_{kz(i)} + \theta^T w_{ij} + u_i 1_{\{k \neq 0\}} + b_{ij} + \varepsilon_{ijk},$$

where $i$ = 1, .., $I$, $j$ = 1,…, $J_i$, $k$= 0, 1, 2, 3 with $k$ = 0 indicating baseline and $k$ = 1, 2, 3 indicating the three post-treatment timepoints, $z(i)$ is the treatment that participant *j* treated by therapist $i$ is randomized to, $\beta_k$ is the time effect (fixed), $\gamma_{z(i)}$ is the treatment effect (fixed), $(\beta\gamma)_{kz(i)}$ is the time by treatment interaction (fixed), $w_{ij}$ denotes the vector of other covariates in the model (such as site or key baseline characteristics) and θ is the associated regression parameter, $u_i$ is the random therapist effect for therapist $i$, $1_{\{k \neq 0\}}$ is the indicator for $k$ not equal to zero, $b_{ij}$ is the random participant effect for participant $j$ treated by therapist $i$, $\varepsilon_{ijk}$ is the random error at the $k^{th}$ timepoint for participant *j* treated by therapist *i*. While other covariance structures will be used to assess their impact on study results, for simplicity and ease of interpretation we assume in the primary analysis that $u_i$, $b_{ij}$ and $\varepsilon_{ijk}$ are independent and have the following distributions:

$$u_i \sim N(0, \sigma_P^2),\ b_{ij} \sim N(0, \sigma_{BS}^2),\ \varepsilon_{ijk}, \sim N(0, \sigma_{WS}^2).$$

Under this mixed effects model, the variance of CAPS total score at timepoint *k* is

$$Var(Y_{ijk}) = \sigma^2 = \sigma_P^2 1_{\{k \neq 0\}} + \sigma_{BS}^2 + \sigma_{WS}^2$$

Note that we allow the improvement in CAPS total score to vary at these three post-treatment timepoints in the mixed effects model; the contrast or estimate statement in SAS will be used to estimate and compare the primary outcome between PE and CPT. Although we anticipate the improvement in CAPS total score established in the treatment course will sustain for 6 months for both PE and CPT, this more flexible model allows the possibility of worsening PTSD symptoms after study treatment is discontinued and the possibility of improving PSTD symptoms if participants initiate other PTSD treatments post study treatment.

*2. Sample size ignoring therapist effect*

For an individual participant with CAPS total score measured at pre-treatment and at *t* post-treatment timepoints, the variance of the primary outcome for this participant is

$$\tau^2 = \sigma_P^2 + \frac{\sigma_{WS}^2}{t} + \sigma_{WS}^2,$$

where $\sigma_P^2$ is the variance of the therapist random effect and $\sigma_{WS}^2$ is the within-subject variation of the CAPS total score. Under the simplified assumptions that all study participants have CAPS total score measured at pre-treatment and at *t* post-treatment timepoints and that each study participant is treated by a different therapist, the sample size per treatment group needed to achieve power *1-β* to detect a mean difference of $\Delta\mu$ between PE and CPT in the primary outcome, using a two-sided t-test with two-sided significance level *α* is:

$$n = \frac{2\left(z_{1-\alpha/2} + z_{1-\beta}\right)^2}{(\Delta\mu/\tau)^2}$$

The estimated variance components from CSP #494 are $\sigma^2 = 678, \sigma_P^2 = 36, \sigma_{BS}^2 = 504, \sigma_{WS}^2 = 174$, and thus $\tau = 16.4$ when *t=3* and $\tau = 17.2$ when *t = 2*. Therefore if we assume all participants have complete follow-up CAPS total score (*t = 3*), it requires a total of 452 participants (226 per group) to have 90% power to detect a difference of $\Delta\mu$ = 5 between PE and CPT in the primary outcome. If each participant has only 2 post-treatment CAPS total scores (*t = 2*), it requires 498 participants (249 per group) to have 90% power.

*3. Adjusting for correlations due to therapist*

In this study, each therapist will deliver either PE or CPT to a number of study participants. Although the treatment will be delivered on an individual basis, observations from the participants treated by the same therapist are likely to be correlated. Assuming each therapist treats *m* study participants, the sample size obtained under the independent participants assumption (in **Section X.B.2**) needs to be inflated by the following inflation factor *f* to retain the same power (Campbell et al., 2007; Machin et al., 2009):

$$f = 1 + (m-1)\rho,$$

where ρ is the intraclass correlation due to therapist, or equivalently the correlation between the primary outcomes from two individuals receiving treatment from the therapist. When each of these two individuals has *t* post-treatment measurements, ρ can be expressed as

$$\rho = \frac{\sigma_P^2}{\tau^2} = \frac{\sigma_P^2}{\sigma_P^2 + \frac{\sigma_{WS}^2}{t} + \sigma_{WS}^2}.$$

Using the variance estimates from CSP #494, ρ = 0.134 when $t = 3$. The Planning Committee determined that it is reasonable to assume each therapist will deliver either PE or CPT to eight participants over the course of the study ($m = 8$). It follows that $f = 1+(8-1)*0.134 = 1.94$. Hence a total of 878 participants (439 per group) is needed to provide 90% power to detect Δμ = 5 in the primary outcome (assuming each participant has baseline CAPS total score and complete follow-up CAPS total score at immediate post-treatment and at 3 and 6 months post treatment) as shown in **Table X.1** below.

***Table X.1 Total sample size needed for a range of parameter values.***

| Δμ | t | τ | Δμ/ τ | ρ | *f* | Power | |
|---|---|---|---|---|---|---|---|
| | | | | | | 85% | 90% |
| 5 | 3 | 16.4 | 0.30 | 0.13 | 1.94 | 750 | 878 |
| 5 | 2 | 17.2 | 0.29 | 0.12 | 1.85 | 788 | 920 |
| 5 | 1 | 19.6 | 0.26 | 0.09 | 1.66 | 916 | 1072 |

We anticipate some participants may not have complete follow-up CAPS total scores at the three post-treatment timepoints. If each participant has $t = 2$ follow-up CAPS total score (instead of 3), then ρ = *0.121, f = 1.85,* and it requires a total of 920 participants (460 per group) to provide 90% power to detect Δμ=5 in the primary outcome, using the same values of variance components as above. In CSP #494, about 75% of participants had all follow-up CAPS total scores, and the average number of follow-up CAPS total scores is 2.45. In this study we expect a higher proportion of participants to have complete follow-up CAPS total score because CAPS assessment will be conducted over telephone, not requiring a clinical visit from the participant. To protect against missing CAPs total scores, potential deviations of the various variances from the assumed values and possible deviation that some therapists will treat more than 8 participants, we plan to randomize 900 participants in this study (450 per group). See

**Table X.2** for the power of the study (with sample size of 900) to detect $\Delta\mu = 5$ or 4 for a range of $\tau$ and $\rho$ values (the number of participants treated by each therapist is fixed at 8).

***Table X.2 Power of the study to detect for $\Delta\mu=5$ or 4 for various vales of $\tau$ and $\rho$.***

| $\Delta\mu$ | $\rho$ | $\tau=16$ | $\tau=17$ | $\tau=18$ | $\tau=19$ | $\tau=20$ |
|---|---|---|---|---|---|---|
| 5 | 0.100 | 95% | 92% | 89% | 86% | 82% |
| | 0.120 | 93% | 90% | 87% | 83% | 79% |
| | 0.134 | 92% | 89% | 85% | 81% | 77% |
| | 0.150 | 91% | 87% | 83% | 79% | 74% |
| 4 | 0.100 | 82% | 77% | 72% | 68% | 63% |
| | 0.120 | 79% | 74% | 69% | 64% | 60% |
| | 0.134 | 77% | 72% | 67% | 62% | 58% |
| | 0.150 | 74% | 69% | 64% | 60% | 55% |

#### *4. Alternative derivation*

We have also derived sample size inflation factor for more general scenarios that allow therapists to treat different numbers of participants and participants to have different numbers of post-treatment CAPS total scores. This sample size inflation factor is calculated by considering the ratio of the variance of the mean change from baseline under the mixed effect model to the variance of the mean change from baseline under the independent observations assumption, in which the mean change from baseline is computed over all individual changes from baseline at all post-treatment points from all participants. While we skip the derivation formula and results here, in the special case that each therapist treats an equal number of participants and each participant has the same number of post-treatment CAPS total score assessed at the same timepoints, the sample size formula reduces to that in the previous subsection.

### C. Number of Participating Sites and Duration of Study

We will select sites that have high patient volume and at least 8 trained therapists (4 PE and 4 CPT) to deliver study treatments. Sites with research infrastructure and research experience are also preferred. Each participating site is expected to randomize 64 participants over the course of the study (8 therapists each treating 8 participants). Therefore, 14.1 sites are needed to achieve the target total randomization of 900. Because some participants are found to be ineligible for the study after signing consent (i.e. during Phase 2 or Phase 3 Screening procedures), the total number of enrolled (i.e. consented) participants in the study may be as high as 2550 (150 per site) to reach the randomization goal of 900.

As mentioned in **Section I.E**, at present 75 sites meet the criterion of having at least 4 therapists who are proficient in PE and 4 who are proficient in CPT, and more sites are expected to qualify as the rollouts continue. **Table I.4** of **Section I.E** shows that many of these qualifying sites have more than 1500 unique PTSD outpatients in FY 2011. We anticipate a large proportion of these PTSD outpatients will be eligible for the current study because our inclusion and exclusion criteria are nonrestrictive. Even if only 5% of these patients are enrolled, a site with at least 512 PTSD patients should be able to randomize 64 patients over a 2.5-year period. We plan to recruit at 17 participating centers at the beginning of the study in case one or more centers have to be terminated.

Each participant will be treated and followed for up to 12 months and, therefore, the total duration of active recruitment, treatment and follow-up is 3.5 years. Start-up and closeout at each VA medical center will both last for 3 months. Start-up and closeout for the Study Chairs' Office, Palo Alto CSPCC and CRPCC at Albuquerque will be 6 months and 3 months, respectively. The Chairs' Office, CSPCC and CRPCC will require 12 months for final statistical analysis.

## D. Final Statistical Analysis

### *1. Baseline comparability*

Because of the large sample size of this study, we expect the randomization process to balance baseline characteristics and produce comparable groups of participants. Baseline comparability between treatment groups will be evaluated with respect to demographic and baseline physical and psychological characteristics. Summary statistics (e.g., means and standard errors for continuous variables, and frequencies and percentages for categorical variables) and graphical techniques (e.g., boxplots for continuous variables, and histograms for categorical variables), will be used to compare the baseline characteristics of the two treatment groups within study sites and the whole study. In addition, we will use t-tests to compare continuous variables and Chi square tests for categorical variables.

### *2. Primary objective*

The primary objective is to compare the effectiveness of PE and CPT for reducing the severity of PTSD symptoms as measured by CAPS total score. The primary outcome is change of CAPS total score from baseline (pre-treatment) to the average in the six months post-treatment (measured at post-treatment and 3 and 6 months follow-up visits).

Primary analysis: The primary analysis will follow the intent-to-treat (ITT) principle. Participants will be counted in the treatment group to which they were randomized, regardless of the number of sessions they completed. Linear mixed effects models, with time, treatment, treatment by time interaction and site as fixed effects and participant and therapist as random effects, will be used to estimate and compare the primary outcome between PE and CPT. The point estimate and the 95% confidence interval for the mean difference will be provided.

Secondary analysis: We will provide point estimates and pointwise 95% confidence intervals for the mean differences in the CAPS total scores at the three post-treatment timepoints as well as the mean differences in changes of CAPS total score from baseline at these post-treatment timepoints. We will also compare the longitudinal profiles of CAPS total score (including the mid-treatment and post-treatment scores) between PE and CPT by testing the treatment by time interaction. When data permits, we will explore site variations in treatment effect and also explore the impact of different covariance variance structures (e.g., allowing the variance of therapist random effect to differ by treatment or allowing certain variances or covariances to vary by time).

Although we anticipate minimum missing data in CAPS total score, we will perform analyses to examine the missing data patterns and the impact of missing data. See **Section X.F** for more details.

As in CSP #494, we will use the CAPS to derive additional measures of clinical outcomes: response (defined as at least 10-point improvement in severity), loss of diagnosis (response plus no longer meeting DSM symptom criteria), and remission (loss of diagnosis plus the DSM-5 score that corresponds to a DSM-IV severity score < 20). The observed proportions and their 95% confidence intervals will be provided for each treatment group at each of the follow-up timepoints. Chi-square tests will be used to compare these outcomes between the two treatment groups.

*3. Secondary objective*

The secondary objective is to compare the effectiveness of PE and CPT for reducing the severity of comorbid mental health problems and service utilization and improving functioning and quality of life.

These secondary outcome measures, listed in **Table X.3** below, will be compared between PE and CPT to support the comparative effectiveness of these two treatments in CAPS total score.

Except for Brief Addiction Monitor and certain service utilization outcomes (which are categorical measures), all other secondary outcomes are continuous measures and will be analyzed in a manner similar to CAPS total score as described above.  Generalized linear mixed effects models (SAS PROC GLIMMIX) will be used to compare the longitudinal profiles of the categorical measures between PE and CPT. Generalized estimating equations may also be used.  We do not plan to adjust for multiple comparisons due to the supportive nature of these secondary outcomes.

***Table X.3  Secondary Outcomes***

| Secondary Outcome | Time Points | Type of Data |
|---|---|---|
| Posttraumatic Diagnostic Scale | Baseline, Post, 3m, 6m | Continuous |
| Beck Depression Inventory-II | Baseline, Post, 3m, 6m | Continuous |
| Spielberger State Anger Inventory | Baseline, Post, 3m, 6m | Continuous |
| Brief Addiction Monitor (2 items) | Baseline, Post, 3m, 6m | Categorical |
| Short Inventory of Problems-Revised | Baseline, Post, 3m, 6m | Continuous |
| WHO-DAS-II | Baseline, Post, 3m, 6m | Continuous |
| WHOQOL-BREF | Baseline, Post, 3m, 6m | Continuous |
| Service Utilization | Baseline, Post, 6m | Categorical, Continuous |
| Client Satisfaction Questionnaire | Post | Continuous |
| PTSD Checklist | weekly during treatment | Continuous |
| Patient Health Questionnaire-9 | weekly during treatment | Continuous |

### *4. Tertiary objective*

The tertiary objective is to examine whether discrepancy between patient preferences and treatment assignment reduces the effectiveness of each treatment.  We will calculate the frequencies and percentages of participants' treatment preference in the entire study sample and also by site, sex, and trauma type.  We will examine the impact of participant's treatment preference on treatment effectiveness.  Participant's treatment preference, collected before randomization, will be included as a covariate in the regression models to assess if the effectiveness of the treatment on the primary and selected secondary outcomes differs between participants who received their preferred treatment and those who did not receive their preferred treatment.  We will also examine if there are differences in treatment adherence and completeness of follow-up.  This analysis will be performed in the combined sample and also within each treatment arm.

*5. Exploratory analyses*

We will explore potential heterogeneities of treatment effect in the primary outcome by performing tests of treatment by subgroup interaction and by displaying treatment effects with their 95% confidence estimates within subgroups. The purpose of these exploratory analyses is to detect apparent reversals of effect or major quantitative interactions, in order to describe the uniformity or variation of effect appropriately. One major subgroup analysis concerns the sex of the participant. By enlisting the help of PBRN, we will try to recruit sites with large pools of female PTSD patients in this study; however, despite our efforts, it is not expected that we will be able to recruit a sufficient number of females to provide more than a preliminary idea of the effect in that subgroup. Other major subgroups will be era, age, race/ethnicity, MST, TBI, comorbid depression, and comorbid substance abuse. Because of the exploratory nature of these analyses, we do not plan to adjust for multiple comparisons.

We will also compare the following variables between PE and CPT: (1) the proportions of study participants meeting the stable remission criterion [early (<12 weeks), on time (12 weeks), late (>12 weeks), did not reach remission], by Chi-square tests; (2) the number of weeks to reach the stable remission criterion, by logrank tests; and (3) the number of sessions and total number of hours delivered, by two-sample t-tests. Mixed effects models or generalized estimating equations may be used to incorporate correlations due to therapists or to explore site variation. Interpretation of these results will incorporate early withdrawal of study treatment, early withdrawal of study, and initiation of non-study treatment for PTSD. We will also examine whether and how treatment dose (such as number of sessions and total number of hours), treatment engagement (homework) and treatment fidelity (therapist adherence and competence) relate to primary and secondary outcomes within and between treatments, and whether and how time to stable remission (such as early vs. on time vs. late) relates to outcomes within and between treatments.

## E. Interim Analysis

In the conduct of clinical trials, there is an ethical obligation to review the data on safety, study conduct and progress, study feasibility, and efficacy over the course of the study. These study data will be reviewed by an independent Data Monitoring Committee (DMC) at least every 6 months or at other frequencies specified by the DMC. A DMC report will be distributed to all members 2-3 weeks prior to meeting. The DMC may recommend early termination of the trial based on interim analyses. At its first meeting, the DMC will discuss and decide how it will

conduct interim monitoring for CSP #591. A prototype set of tables and figures for purposes of monitoring are given in the appendix section called, “Biostatistical Research Data Processing (BRDP)”.

*1. Monitoring of safety and study progress*

Study safety will be monitored by CSPCC and CSP Clinical Research Pharmacy Coordinating Center (CSPCRPCC), and reported to the DMC at least every 6 months. A complete description of monitoring and reporting adverse events is given in **Section XI**. In the event that serious adverse events are noted to be excessive in either group, the DMC may consider recommending the study be stopped.

In addition to interim monitoring for safety, the DMC will also monitor patient intake (overall and within site), site adherence to the study protocol, data quality, completeness and timeliness of follow up and data submission, and baseline comparability of treatment groups. The DMC will review the accumulating data and be responsible for determining whether or not to recommend to the CSR&D Director that the trial be continued or stopped. Data summaries will be prepared for the DMC for these purposes. To aid the DMC in their deliberations, other relevant information pertaining to (e.g., secondary analyses) and outside of (e.g., other studies) CSP #591 will be made available.

*2. Interim analysis for potential early stopping for efficacy or futility*

The study does not plan to conduct interim analyses to allow early stopping of the study for efficacy (when there is sufficient evidence that one treatment is superior to the other treatment) or for futility (when it is futile to establish a statistical significant difference at the end of the trial). The rationales are (1) both PE and CPT are effective treatments for PTSD, so there are no ethical concerns in continuing the study even when it is unlikely to establish a statistical significant difference at the end of the trial; (2) even when there are treatment differences between PE and CPT, the differences are not likely to alter the VA policy to make all evidence-based treatment available to PTSD patients based on interim analysis results; (3) it is important from a public health, policy, and scientific perspective to collect sufficient data on the secondary outcomes to support findings in the primary outcome, in the hope that the totality of the evidence will be able to provide guidance to or change clinical practice; (4) it allows us to examine the impact of patient preference on treatment effectiveness, which is one of the key elements in personalized medicine.

## F. Procedure for Handling Missing Data

It is expected that there will be some missing data. Imputation techniques, such as linear interpolation and multiple imputation methods will be examined to assess the robustness on the results. Completer analyses will also be done based on participants who remained in the study throughout the 6-month follow-up period. When large fractions of information are missing, we will perform sensitivity analyses under weaker assumptions (e.g., non-ignorable missingness). It is recognized, however, that the best approach to missing data is to make all efforts to minimize it, since imputation is difficult when the missing data are non-ignorable or not missing at random. We will attempt to collect outcome data from all participants at all timepoints regardless of whether they continue or complete the study treatment. We will also attempt to collect reasons for missing data when possible. Also, the telephone CAPS assessment can facilitate completeness by enhancing the convenience for participants, who will not have to travel for assessment sessions.

## G. Procedures for Reporting Modifications to the Original Statistical Plan

Changes to the original statistical plan for analyzing study data will follow the CSP standard operating procedures for amending the study protocol, which require review and approval by the CSPCC Director and several oversight groups including the Executive Committee and the DMC. As needed, updates may also be required to other study documents such as the Case Report Forms and Operations Manual.

# XI. MONITORING AND REPORTING ADVERSE EVENTS

## A. Importance of Adverse Event Reporting

Timely and complete reporting of safety information assists study management in identifying any untoward medical occurrence, thereby allowing: a) protection of safety of study participants, b) a greater understanding of the overall safety profile of the study treatments and therapeutic modalities, c) improvements in study design or procedures, and d) compliance with regulatory requirements.

## B. Role of the Local Site Investigator in Adverse Event Monitoring

The LSI will be responsible for the adverse event reporting requirements as outlined below:

- Reviewing the accuracy and completeness of all adverse events (AE) reported.
- Compliance with VA CIRB policies for reporting AEs and/or serious adverse events (SAEs).

[✳ Note: In June 2015, the VA published an update to “Reporting of Adverse Events in Research to the Office of Research Oversight (ORO)” in VHA Handbook 1058.1. Investigators should be aware of these reporting requirements. This, however, does not eliminate the need for investigators to report both adverse events and serious adverse events to the CSP #591 Sponsor as per the study protocol.]

- Closely monitoring research participants at each study visit for any new SAEs.

## C. Collection of Safety Information

### *1. Adverse Events*

Adverse events (AEs) are defined by the International Conference on Harmonization (ICH) for Clinical Safety Data Management (ICH-E2A) as “any untoward medical occurrence in a clinical investigation subject that is subjected to one of the study treatments that does not necessarily have to have a causal relationship with the treatments. An AE, therefore, can be any unfavorable or unintended sign (including an abnormal laboratory finding), symptom, or disease temporally associated with the study interventions.”

For the purposes of CSP #591, the study treatments are a) the Prolonged Exposure (PE) treatment and related activities and b) the Cognitive Processing Therapy (CPT) treatment and related activities.

In this study, information on AEs related to or possibly related to study treatments and on all serious adverse events (SAEs) will be collected and recorded. See the section below on "Relatedness". There is a separate section below that describes the collection of safety information for SAEs.

The reporting period for AEs begins when the participant signs the informed consent form and continues until the participant's completion or early termination of study participation or the end of the study. Each related/possibly related AE will be reported to the Sponsor, the VA Cooperative Studies Program, including any increase in frequency or severity of a condition that was present prior to the start of the study. During the study, adverse events can be spontaneously reported or elicited during open-ended questioning, examination, or evaluation of the participant at study visits.

Related or possibly related adverse events not meeting the criteria for an SAE (see below) must be recorded on the Adverse Event Form. (Those that meet SAE criteria are documented on the SAE Form). One form should be completed for each AE reported. Adverse events should be reported in sequential order as they occur and submitted with the other case report forms for the participant's visit.

a. Adverse Event Classification

*Severity:*

| | |
|---|---|
| Mild | Does not interfere with normal activity (not reportable) |
| Moderate | Interferes with normal activity to some extent |
| Severe | Interferes significantly with normal activity |

Note: Only AEs classified as either "Moderate" or "Severe" must be reported.

*Examples of Mild (not reportable) vs. Moderate (reportable) Adverse Events*

1. Pre-existing conditions that are documented in the medical record at the time of informed consent: only report as AEs (moderate or severe) those that increase in frequency and or severity.
2. Arthritis: Normal waxing and waning – do not report. Pain that requires a steroid injection or prevents patient from moving around as normal → report as an AE.
3. Headache: Common type of headache that may require a non-steroidal agent that occurs every once in awhile → do not report as an AE. Migraine-like headache that

keeps the patient in bed with no light or prevents him/her from going to work → report as an AE.

4. Report any adverse event after informed consent that is associated with new diagnoses, e.g. hypertension, anxiety, depression, etc.
5. Common colds: Do not report as an AE unless cold develops into pneumonia or some other type of serious upper respiratory infection.
6. To further differentiate between mild and moderate – if patient can still do normal activities of living in spite of the “adverse event” → do not report as an AE. If the AE prevents the patient from being able to do one or more activities of daily living, it would be considered “moderate” → report as an AE. If the AE results in the patient needing to go to the ER or hospital, it would be considered an SAE and should also be reported on an SAE Form.

b. Relatedness

The investigator and sub-investigators are responsible for determining if an adverse event may be related with one of the study treatments based on their medical expertise, familiarity with the therapeutic category of the study treatment, and the emergence of the general clinical picture developed over the course of the study. Recall that only related or possibly related AEs that do not meet SAE criteria are reported on study forms.

Relatedness involves an assessment of the degree of causality (attributability) between the study intervention and the event. The assessment provided by the LSI is part of the information used by the sponsor to determine if the adverse event presents a patient safety concern. Pursuant to CSP Global SOP 3.6, an AE is deemed to be associated with the use of a study intervention if “[t]here is a reasonable possibility that the experience may have been caused by the intervention or by participation in the trial.” Thus, all adverse events with a reasonable causal relationship to the study intervention should be considered “related”. A definite relationship does not need to be established. The following levels of relatedness will be used in this trial:

- Not attributed to a study intervention
- Possibly attributed to a study intervention
- Attributed to a study intervention

c. Adverse Event Follow-Up

For each reported AE, investigators follow up with participants until the event resolves and ensure that appropriate care is provided, but there is no case report form to fill for AE follow-up. Adverse events must be reported as Serious Adverse Events (SAE) if they meet the SAE reporting requirements described below.

*2. Serious Adverse Events*

a. Definition of Serious Adverse Event (SAE)

Serious adverse events are defined by the ICH for Clinical Safety Data Management and CSP Global SOP 3.6.3, as any untoward medical occurrence that:

- Results in death,
- Is life threatening,
- Requires inpatient hospitalization or prolongation of existing hospitalization,
- Results in persistent or significant disability or incapacity,
- Is a congenital anomaly/birth defect, or
- Is an important medical event that may not result in death, be life-threatening, or require hospitalization (an event may be considered serious when, based upon appropriate medical judgment, it may jeopardize the participant and may require medical or surgical intervention to prevent one of the outcomes listed in this definition).

Any adverse event that meets the definition of “Serious” will be reported on an SAE Form. All SAEs will be classified as either “related,” "possibly related," or “not related” to study intervention. A definite causal relationship does not need to be established.

b. Serious Adverse Event Monitoring

Participants will be monitored for SAEs at each study visit. Each serious adverse event is reported on an SAE Form. Active monitoring for SAEs begins at the time the Informed Consent Form is signed and continues until the earlier of the 30 days after the participant’s completion or early termination of study participation or the end of the study. The date study participation ends is entered on the Study Completion/Termination Form.

## D. Expedited Reporting of Serious Adverse Events

All SAEs require prompt reporting to the CSP Clinical Research Pharmacy Coordinating Center (CSPCRPCC) within 72 hours of the LSI becoming aware of the event. The CSP #591 Adverse Event (AE) Specialist at the CSPCRPCC is responsible for evaluating all SAEs for patient safety concerns and regulatory reporting. The AE Specialist will consult with the Chairman's office during the review process, as necessary. CSPCPRCC maintains a database of serious events for evaluation, by using the Medical Dictionary for Regulatory Activities (MedDRA) for coding and trending. Periodic summaries will be provided to the Data Monitoring Committee, the Study Chairman's office and Executive Committee (as necessary). Events that are determined to be serious, unexpected, and related to the study treatments will be reported to the LSIs, CIRB, and to the VA Cooperative Studies Program Central Office.

SAE Forms will be sent to the Palo Alto CSPCC. The CSPCRPCC will also have access to the information on the SAE Forms.

### *1. SAE Follow-up Reporting*

Serious adverse events should be followed to resolution, stabilization, or the end of the study, whichever occurs first. If an SAE is still ongoing by the time the SAE Form is submitted to the Palo Alto CSPCC, complete an SAE Follow-up Form every 30 days until the SAE is resolved or stabilized. SAE Follow-up Forms will be sent to the Palo Alto CSPCC.

## E. Reporting Adverse Events and Serious Adverse Events to the VA Central IRB

It is the responsibility of the LSI / SC at each participating site to know and comply with the AE and SAE reporting requirements of the VA CIRB. Information on VA CIRB's reporting requirements can be found on the VA CIRB website (http://www.research.va.gov/vacentralirb/) or by contacting the AE Specialist.

Questions about managing or reporting of adverse events or serious adverse events will be addressed by the AE Specialist at the CSPCRPCC or the CSP #591 National Study Coordinator. In all instances of AE, Central IRB procedures and VHA Handbook 1058.01 will be followed.

# XII. QUALITY CONTROL

## A. Standardization/Validation of Measurements

Details about quality control for assessment procedures are provided in **Sections VIII.A.3-5** (procedures to enhance completion of assessment protocols, double-blinding, and reliability).

## B. Treatment

Details about quality control for treatment are provided in **Sections VIII.F** (training and supervision) and **VIII.G** (therapy fidelity monitoring).

## C. Masking

Centrally located interviews will perform assessment by telephone, which minimizes the risk of unblinding. In addition, assessors will remind participants not to reveal their treatment assignment during the assessment interviews.

## D. Monitoring Participant Intake and Probation/Termination of Participating Centers

During the course of a study, it may be necessary to drop one or more participating medical centers from the study. Such action must have the prior approval of the CSPCC Director and the Director of VA CSR&D. Early termination is usually based on recommendations from the Executive Committee and the DMC and most often reflects inadequate participant intake or serious noncompliance with Good Clinical Practices. This action should always be based on the best interests of the study and study participants and does not necessarily imply poor performance on the part of the SI or the medical center. Termination will be conducted per CSP guidelines.

### *1. Enrollment Issues*

The Study Chairs and the Study Biostatistician will monitor the intake rate and operational aspects of the study. Participating medical centers will continue in the study only if adequate participant intake is maintained. The Executive Committee may take action leading to the discontinuation of enrollment at a center with the concurrence of the CSPCC Director and the Director of VA CSR&D. If recruitment is not proceeding at an appropriate rate, the Study Chairs and Study Biostatistician will scrutinize the reasons for participant exclusions and other barriers to recruitment. Based on this information, the Executive Committee may choose, with the approval of the DMC and the Director, VA CSR&D, to drop centers, add additional centers,

make minor modifications to the inclusion/exclusion criteria, or extend the recruitment period. Participating sites that enroll below target during the first 12 months of the study may be placed on probation and given an opportunity to improve within a reasonable period. If a medical center is placed on probation, the Study Chairs will confer with the site personnel and may visit the site, if necessary, to help improve the rate of recruitment. If there is no improvement in accrual during the probation period, the site may be subject to reduced funding or possible termination as a study site. To prevent the delay in adding new sites, we plan to start the study with 17 recruiting sites, which is about 3 more than needed from the sample size requirement of 14.1. The Executive Committee will take actions leading to discontinuation of a site only with the concurrence of the CSPCC Director. If a site is terminated from the trial, resources will be reallocated to other medical centers or used to start up a back-up site.  Central IRB will be informed of all site terminations and probations.

*2. Non-adherence to the protocol and/or Good Clinical Practice (GCP) Guidelines*

Strict adherence to the protocol and GCP guidelines will be expected of every participating medical center and monitored by the DMC, the Executive Committee, and the Study Group. Documentation of protocol violations will be required.  Medical centers with repeated protocol violations or repeated failures to follow GCP Guidelines will be recommended for termination to the DMC, the CSPCC Director, and the Director of VA CSR&D. If a participating investigator feels that adherence to the protocol may result in an apparent immediate hazard to the participant, the interest of the participant must take precedence.

Protocol violations must be reported to the CSPCC on the appropriate case report form and to the Central IRB to ensure immediate hazard to the participant did not occur.

By agreeing to participate in the study, the medical center delegates responsibility for global monitoring of the ongoing study to the Cooperative Studies Program Committee and personnel listed above. However, the Research and Development (R&D) Committee and the VA Central IRB may require the participating investigator to submit annual and final progress reports concerning the status of the study at the medical center for local monitoring purposes.

## XIII. ORGANIZATION & ADMINISTRATION

The organizational and administrative structure of this cooperative study will be similar to others in the Cooperative Studies Program. Specifically, it will include the following components:

The Cooperative Studies Program (VA Central Office) establishes overall policies and procedures which are applied to all VA cooperative studies through the Study Chairs' offices, and the Palo Alto CSPCC.

The Palo Alto CSPCC and the Study Chairs offices jointly will perform the day-to-day scientific and administrative coordination of the study. These include developing the study protocol, operations manual, and case report forms; ensuring the appropriate support for the participating centers; scheduling meetings and conference calls; answering questions about the protocol; conducting site visits; publishing newsletters; preparing interim and final progress reports; and archiving study data at the end of the study. Interim statistical progress reports will be produced every six months. Participant accrual and data quality will be monitored closely to ensure that the study is progressing satisfactorily.   The PBRN Coordinating Center will assist with advising on strategies for recruitment and retention of women, monitoring recruitment and retention of women, and conducting entry and analysis VFF data.

The CSP Clinical Research Pharmacy Coordinating Center (CSPCRPCC) provides advice and consultation about protocol development, procedure implementation, and participant safety issues. CSPCRPCC is responsible for monitoring and reporting the safety of trial participants through the review, assessment, and communication of adverse events and serious adverse events reported by study personnel with reviewing responsibilities occurring through ongoing communication with the Study Chairs, Executive Committee, Data Coordinating Center, and CSP Central Office. The reporting activities include the filing of regulatory documents involving adverse events to meet federal regulations and CSP policies. In conjunction with the Data Coordinating Center, the CSPCRPCC trends and analyzes safety data in order to prepare reports for various committees including the Data Monitoring Committee (DMC), VA Central Institutional Review Board (CIRB), Study Executive Committee(s), and study investigator meetings.

Each participating VA medical center will designate a Local Site Investigator (LSI) to be responsible administratively and scientifically for the conduct of the study at the center. LSIs will be expected to attend all annual Study Group meetings, as well as to hire and supervise personnel. By agreeing to participate in the study, the medical center delegates responsibility for global monitoring of the ongoing study to the DMC, and the Cooperative Studies Scientific Evaluation Committee. However, the Research and Development Committee (R&D) and the Cental IRB may require the participating investigator to submit annual reports concerning the

status of the study at the medical center for local monitoring purposes.

The Cooperative Studies Scientific Evaluation Committee (CSSEC) reviews the scientific merit of all new cooperative study proposals and all ongoing cooperative studies as deemed necessary. The committee is composed of both VA and non-VA clinical research scientists, most of whom have had experience in managing their own cooperative studies.

The Study Group will be composed of the SIs from each participating center, the Study Chairs, and CSP staff (Biostatistician, Project Manager, Clinical Research Pharmacist, and others). The Study Chairs will head the group, which will meet once per year to discuss the progress of the study, any problems that the investigators have encountered, and any suggestions for improving the study.

The Executive Committee will be concerned with the overall management of the study. It will be headed by the Study Chairs and will consist of the study Biostatistician, study Project Manager, Clinical Research Pharmacist, selected participating investigators, and outside consultants as needed. This committee will meet every month to review study conduct and progress, decide upon changes in the study, determine the fate of hospitals whose performance is substandard, initiate any sub-protocols, and discuss publication of the study results.

The Data Monitoring Committee (DMC) will provide independent and unbiased reviews of the study's progress and will monitor patient intake, outcomes, adverse events, and other issues related to patient safety. This committee will be composed of two biostatisticians and several physicians with expertise in the subject area(s) of the study. The Study Chairs, study Biostatistician, study AE Specialist, and the Director of the CSPCC are *ex officio* (liaison, non-voting) members of the committee. The DMC  will meet every 6 months to monitor the study. Its primary responsibility is to review the progress of the study and to decide whether or not the study should continue. To help them make their assessment, the Study Chairs and Study Biostatistician will furnish the DMC with appropriate monitoring data before each meeting.

The Palo Alto CSPCC Human Rights Committee (HRC), composed primarily of lay people, may be called upon to review new protocols, periodically make site visits to participating centers to monitor participant involvement in the study, and review and consult on any ethical and human rights issues that arise during the conduct of the study. Prior to participation, each site's local R&D and the VA Central IRB (CIRB) must also review and approve its involvement in the study.

The CSP Site Monitoring, Auditing and Resource Team (SMART), located at the CSP Clinical Research Pharmacy Coordinating Center (CSPCRPCC) in Albuquerque, NM will monitor the trial for compliance with Good Clinical Practices (GCP). GCP reviewers from SMART will visit participating sites shortly after enrollment is initiated to monitor investigator regulatory compliance, protocol adherence, and overall research practices. In addition to the regularly scheduled GCP review visits, an independent comprehensive GCP site audit may be conducted at any time at the request of CSP study management.

## XIV. GOOD CLINICAL PRACTICE (GCP)

### A. Role of GCP

This trial will be conducted in compliance with Good Clinical Practice (GCP) regulations. The intent of these regulations is to safeguard subjects' welfare and assure the validity of data resulting from the clinical research. The VA Cooperative Studies Program will assist Local Site Investigators (LSIs) in complying with GCP requirements through its Site Monitoring, Auditing and Resource Team (SMART) based in Albuquerque, NM. SMART serves as the Quality Assurance arm of CSP for GCP compliance. Study site personnel will receive GCP training at the study organizational meeting. SMART will provide training, manuals and materials to assist study personnel in organizing study files and will be available throughout the trial to advise and assist LSIs regarding GCP issues.

### B. Summary of Monitoring and Auditing Plans

*1. Monitoring Visits*

- Initiation visits at each site soon after study start-up
- Additional monitoring visits may be conducted as deemed necessary by study leadership or SMART.

*2. Audits*

- Routine audits – independent site visits to one or more sites per year as determined by SMART.
- For-Cause audits –independent audit of a site as requested by study leadership or CSP Central Office.
- Audits may be scheduled or unannounced.

## XV. PUBLICATIONS

### A. Publication Plan

It is the policy of the CSP that outcome data will not be revealed to the participating investigators and Study Chairman until the data collection phase of the study is completed. This policy safeguards against possible biases affecting the data collection.

All presentations and publications from this study will be done in accordance with the CSP policy as stated in the CSP Guidelines. The presentation or publication of any data collected by investigators on participants entered into the VA cooperative study is under the control of the study's Executive Committee. This is true whether the publication or presentation is concerned with the results of the principal undertaking or is associated with the study in some other way. No individual participating investigator is permitted to perform analyses or interpretations or to make public presentations or seek publication of any of the data other than under the auspices and approval of the Executive Committee.

The Executive Committee has the authority to establish one or more publication committees, usually made up of subgroups of participating investigators and some members of the Executive Committee, for the purpose of producing manuscripts for presentation and publication. All presentations and publications will be circulated to all participating investigators for their review, comments, and suggestions, at least four weeks prior to submission of the manuscript to the presenting or publication body.

Authorship should include the Study Chairs, the National Study Coordinator, members of the Executive Committee, and Participating Investigators from top recruiting centers. The number of authors will not exceed individual journal limitations. All publications must give proper recognition to the Study's funding source. If an investigator's major salary support and/or commitment are from the VA, the investigator must list the VA as his/her primary institutional affiliation. Submission of manuscripts or abstracts must follow the usual VA policy. Ideally, a subtitle is used stating, "A VA Cooperative Study." A copy of the letter to the editor and the manuscript/abstract submitted for publication/presentation should be sent to the CSP Director, and for information purposes, to the members of the study's DMC. The CSP also requires that a copy of every manuscript must be reviewed and approved by the CSPCC Director prior to submission as a last quality control step.

## B. Planned publications

The following is a list of proposed manuscripts for CSP #591:

*1. Comparative Effectiveness of Prolonged Exposure and Cognitive Processing Therapy for the Treatment of PTSD*

This manuscript will present findings pertaining to the primary and secondary objectives of the study: (1) to compare the effectiveness of Prolonged Exposure and Cognitive Processing Therapy for reducing the severity of PTSD symptoms; and (2) to compare the effectiveness of Prolonged Exposure and Cognitive Processing Therapy for reducing the severity of comorbid mental health problems and service utilization and improving functioning and quality of life.

*2. Effect of Participant Preferences for Treatment on Response to Prolonged Exposure and Cognitive Processing Therapy for PTSD*

This manuscript will describe sociodemographic and clinical factors related to preference for Prolonged Exposure versus Cognitive Processing Therapy and will examine whether receiving preferred treatment affects treatment outcome.

*3. Predictors of Clinical Outcome in Cognitive-Behavioral Therapy for PTSD*

This manuscript will examine whether pretreatment sociodemographic and clinical factors (such as gender, race/ethnicity, era, type of trauma, MST, TBI, and comorbidity) differentially predict response to and dropout from Prolonged Exposure and Cognitive Processing Therapy.

*4. The Longitudinal Course of Symptoms during and after Cognitive-Behavioral Therapy for PTSD*

This manuscript will compare the course of change in PTSD and depression during treatment and after treatment and second, during the entire study period, using the study by Resick et al. (2008) as a model. Individual trajectories of symptom change will be explored, and baseline factors associated with these patterns will be examined.

*5. Symptomatic Versus Functional Outcomes in Cognitive-Behavioral Therapy for PTSD*

This manuscript will examine longitudinal relationships between symptoms and functional outcomes during and after the course of treatment, using the manuscript by Schnurr et al. (2006) as a model.

*6. Design of CSP #591: A Comparative Effectiveness Trial of Cognitive-Behavioral Therapies for PTSD*

This manuscript will describe the design of CSP #591. We will describe unique challenges we faced in the designing the study and the rationale for decisions about the methods.

## XVI. REFERENCES


Agency for Healthcare Research and Quality. (2012). *Psychological treatments and pharmacological treatments for adults with posttraumatic stress disorder (PTSD)*. Draft Comparative Effectiveness Review. Rockville, MD: Author.

American Psychiatric Association Work Group on ASD and PTSD. (2004). *Practice guidelines for the treatment of patients with acute stress disorder and posttraumatic stress disorder.* Washington, DC: American Psychiatric Association.

American Psychiatric Association. (1994). *Diagnostic and statistical manual of mental disorders*. Washington, DC: Author.

Asukai, N., Saito, A., Tsuruta, N., et al. (2010). Efficacy of exposure therapy for Japanese patients with posttraumatic stress disorder due to mixed traumatic events: A randomized controlled study. *Journal of Traumatic Stress*, *23*, 744-750.

Attkisson, C.C., & Zwick, R.J. (1982). The Client Satisfaction Questionnaire: Psychometric properties and correlations with service utilization and psychotherapy outcome. *Evaluation and Program Planning*, *5*, 233-237.

Aziz, M.A., & Kenford, S. (2004). Comparability of telephone and face-to-face interviews in assessing patients with posttraumatic stress disorder. *Journal of Psychiatric Practice, 10*, 307-313.

Baldwin, S.A., & Berkeljon, A. (2009). Rates of change in naturalistic psychotherapy: Contrasting dose-effect and good-enough level models of change. *Journal of Consulting and Clinical Psychology*, 77, 203-2011.

Beck, A.T., Steer, R.A., Ball, R., et al. (1996). Comparison of Beck Depression Inventories -IA and -II in psychiatric outpatients. *Journal of Personality Assessment, 67*, 588-597.

Bliese, P.D., Wright, K.M., Adler A.B., et al. (2008). Validating the Primary Care Posttraumatic Stress Disorder Screen and the Posttraumatic Stress Disorder Checklist with soldiers returning from combat. *Journal of Consulting and Clinical Psychology*, *76*, 272-281.

Borkovec, T.D., & Nau, S.D. (1972). Credibility of analogue therapy rationales. *Journal of Behavior Therapy and Experimental Psychiatry, 3,* 257–260.

Cacciola, J.S., Alterman, A.I., DePhilippis, D., et al. (in press). Development and initial evaluation of the Brief Addiciton Monitor (BAM). *Journal of Substance Abuse Treatment.*

Campbell, MJ, Donner, A., & Klar, N. (2007). Developments in cluster randomized trials and statistics in medicine. *Statistics in Medicine*, *26*, 2-19.

Chard, K. (2005). An evaluation of Cognitive Processing Therapy for the treatment of posttraumatic stress disorder related to childhood sexual abuse. *Journal of Consulting and Clinical Psychology*, 73, 965-971.

Chard, K., Schumm, J., McIlvain, S., et al. (2011). *Examining the effectiveness of CPT-Cognitive (CPT-C) for veterans with PTSD and traumatic brain injury in a residential setting*. *Journal of Traumatic Stress, 24*, 347-351.

Chard, K., Schumm, J., Owen, G., et al. (2010). A comparison of OEF/OIF and Vietnam veterans receiving Cognitive Processing Therapy. *Journal of Traumatic Stress, 23*, 25-32.

Cohen, J. (1988) *Statistical power analysis for the behavioral sciences* (2nd ed.). Hillsdale, NJ: Erlbaum.

Cook, J.M. (Principal Investigator). (ongoing) Sustained use of Evidence-based PTSD treatment in VA residential settings. National Institute of Mental Health 1R01MH096810-01A1.

Cook, J.M., O-Donnell, C.O., Dinnen, et al. (2012). Measurement of a model of implementation for health care: toward a testable theory. *Implementation Science, 7*.

Departments of Veterans Affairs and Defense. (2010). *VA/DoD Clinical practice guideline for management of post-traumatic stress.* Washington, DC: Author.

Ehlers, A., Clark, D.M., Hackmann, A., et al. (2005). Cognitive therapy for post-traumatic stress disorder: Development and evaluation. *Behaviour Research and Therapy, 43*, 413-431.

Falik, D., Wang, X.Q., Liu, L., et al. (2010). Percentage of subjects with no heavy drinking days: Evaluaiton as an efficacy endpoint for alcohol clinical trials. *Alcoholism: Clinical and Experimental Research, 34*, 2022-2034.

Feeny, N.C., Hembree, E.A., & Zoellner, L.A. (2003). Myths regarding exposure therapy for PTSD. *Cognitive and Behavioral Practice, 10*, 85-90.

Feeny, N.C., Zoellner, L.A., Mavissakalian, M., et al. (2010, November). *A doubly randomized preference trial: Prolonged Exposure versus sertraline for PTSD*. Annual Meeting of the Association of Behavioral and Cognitive Therapy. Toronto, Canada.

First, M.B., Spitzer, R.L., Gibbon, M., et al. (2002). Structured Clinical Interview for DSM-IV-TR Axis I Disorders, Research Version, Patient Edition. (SCID-I/P) New York: Biometrics Research, New York State Psychiatric Institute.

Foa E.B., & Cahill S.P. (2001). Psychological therapies: Emotional processing. In Smelser, N.J., Bates P.B.,(Eds.), *International Encyclopedia of Social and Behavioral Sciences* (pp.12363-12369). Oxford: Elsevier.

Foa, E.B., & Kozak, M.J. (1986). Emotional processing of fear: Exposure to corrective information. *Psychological Bulletin, 99*, 20-35.

Foa, E.B., Cashman, L., Jaycox, L., et al. (1997). The validation of a self-report measure of posttraumatic stress disorder: The Posttraumatic Diagnostic Scale. *Psychological Assessessment, 9*, 445-451.

Foa, E.B., Dançu, C.V., Hembree, E.A., et al. (1999). A comparison of exposure therapy, stress inoculation training, and their combination for reducing posttraumatic stress disorder in female assault victims. *Journal of Consulting and Clinical Psychology, 67,* 194-200.

Foa, E.B., Hembree, E.A., & Rothbaum, B.O. (2007). *Prolonged Exposure therapy for PTSD: Emotional processing of traumatic experiences*. *Therapist's guide*. New York: Oxford.

Foa, E.B., Hembree, E.A., Cahill, S.P., et al. (2005). Randomized trial of prolonged exposure for posttraumatic stress disorder with and without cognitive restructuring: Outcome at academic and community clinics. *Journal of Consulting and Clinical Psychology, 73*, 953-964.

Foa, E.B., Keane, T.M., Friedman, M.J., et al. (Eds.). (2008). *Effective treatments for PTSD: Practice guidelines from the International Society for Traumatic Stress Studies* (2nd ed.). New York: Guilford Press.

Fontana, A., & Rosenheck, R. (2008). Treatment-seeking veterans of Iraq and Afghanistan: Comparison with veterans of previous wars. *Journal of Nervous and Mental Disease*, *196*, 513−521.

Galovski, T. E., Blain, L., Mott, J., et al. (in press). Manualized therapy for PTSD: Flexing the structure of Cognitive Processing Therapy. *Journal of Consulting and Clinical Psychology*.

Greenhalgh, T., Glenn, R., Bate, P., et al. (2005). *Diffusion of innovations in health service organizations: A systematic literature review*. Oxford, England: Blackwell Publishing.

Griffin, M.G., Uhlmansiek, M.H., Resick, P.A., et al. (2004). Comparison of the Posttraumatic Stress Disorder Scale versus the Clinician-Administered Posttraumatic Stress Disorder Scale in domestic violence survivors. *Journal of Traumatic Stress, 17*, 497-503.

Hembree, E.A., Foa, E.B., Dorfan, N.M., et al. (2003). Do patients drop out prematurely from exposure therapy for PTSD? *Journal of Traumatic Stress, 16*, 555-562.

Hermes, E.D.A., Rosenheck, R.A., Desai, R.A., et al. (2012). Recent trends in the treatment of posttraumatic stress disorder and other mental disorders in the VHA. *Psychiatric Services,* 63, 471–476.

Hoge, C.W., McGurk, D., Thomas, J.L., et al. (2008). Mild traumatic *brain* injuries in U.S. soldiers returning from Iraq. *New England Journal of Medicine*, *358*, 453-463.

Howard, K.I., Kopta, M.S., Krause, M.S, et al. (1986). The dose-effect relationship in psychotherapy. *American Psychologist*, *41*, 159-164.

Institute of Medicine (2008). *Treatment of posttraumatic stress disorder: An assessment of the evidence*. Washington, DC: National Academies Press.

Institute of Medicine (2009). *Initial national priorities for comparative effectiveness research*. Washington, DC: National Academies Press.

Jacobson, N.S., & Truax, P. (1991) Clinical significance: A statistical approach to defining meaningful change in psychotherapy research. *Journal of Consulting and Clinical Psychology*, *59*, 12-19.

Karlin, B.E., Ruzek, J.I., Chard, K.M., et al. (2010). Dissemination of evidence-based psychological treatments for posttraumatic stress disorder in the Veterans Health Administration**.** *Journal of Traumatic Stress*, *23*, 663-673.

Kessler, R.C., Chiu, W.T., Demler, O., et al. (2005). Prevalence, severity, and comorbidity of 12-month DSM-IV disorders in the National Comorbidity Survey Replication. *Archives of General Psychiatry, 62,* 617-627.

Kessler, R.C., Petukhova, M., Sampson, N.A., et al. (2012). Twelve-month and lifetime prevalence and lifetime morbid risk of anxiety and mood disorders in the United States. *International Journal of Methods in Psychiatric Research, 21*, 169-184.

Kessler, R.C., Sonnega, A., Bromet, E., Hughes, M., & Nelson, C.B. (1995). Posttraumatic stress disorder in the National Comorbidity Survey. *Archives of General Psychiatry*, *52*, 1048-1060.

Kiluk, B.D., Dreifuss, J.A., Weiss, R.D., et al. (in press). The Short Inventory of Problems-Revised: Psychometric properties within a large, diverse sample of substance use disorder treatment seekers. *Psychology of Addictive Behaviors*.

Kimerling, R., Gima, K.S., Smith, M.W., et al. (2008). The Veterans Health Administration and military sexual trauma. *American Journal of Public Health*, *97*, 2160-2166.

Kroenke, K., Spitzer, R.L., & Williams, J.B.W. (2001). The PHQ-9: Validity of a brief depression severity measure. *Journal of General Internal Medicine*, *16*, 606-613.

Kulka, R.A., Schlenger, W.E., Fairbank, J.A., et al. (1990). *Trauma and the Vietnam War generation: Report of findings from the National Vietnam Veterans Readjustment Study.* New York: Brunner/Mazel.

Levitt, J.T., Malta, L.S., Martin, A., et al. (2007). The flexible application of a manualized treatment for PTSD symptoms and functional impairment related to the 9/11 World Trade Center attack. *Behaviour Research and Therapy, 45*, 1419-1433.

Linehan, M.M., Comtois, K.A., & Ward-Ciesielski, E.F. (2012). Assessing and managing risk with suicidal individuals. *Cognitive and Behavioral Practice, 19*, 218-232.

Lowe, B., Unützer, J., Callahan, C.M., et al. (2004). Monitoring depression treatment outcomes with the Patient Health Questionnaire-9. *Medical Care*, *42*, 1194-1201.

Machin, D., Campbell, M.J, Tan, S.B., et al. (2009). *Sample size tables for clinical studies, 3rd ed*. (Chapter 6). Oxford, UK: Wiley-Blackwell.

Magruder, K.M., Frueh, B.C., Knapp, R.G., et al. (2005). Prevalence of posttraumatic stress disorder in Veterans Affairs primary care clinics. *General Hospital Psychiatry, 27*, 169-79.

Miser, WF, Wallace, LS. Research Study Participant Satisfaction Survey. The Ohio State University Center for Clinical and Translational Science. Request online: https://ccts.osu.edu/research-support-services/recruitment-and-retention/research-study-participant-satisfaction-survey

Monson, C.M., Fredman, S., MacDonalnd, A., et al. (2012). Effect of cognitive-behavioral couple therapy for PTSD: A randomized controlled trial. *JAMA, 308*, 700-709.

Monson, C.M., Gradus, J.L., Young-Xu, Y., Schnurr P.P., Price, J.L., & Schumm, J.A. (2008). Change in posttraumatic stress disorder symptoms: Do clinicians and patient agree? Psychological Assessment, 20, 131-138.

Monson, C.M., Schnurr, P.P., Resick, P.A., et al. (2006). A randomized controlled trial of Cognitive Processing Therapy for veterans with military-related posttraumatic stress disorder. *Journal of Consulting and Clinical Psychology*, *74*, 898-907.

Nacasch, N., Foa, E.B., Huppert, J.D., et al. (2011). Prolonged exposure therapy for combat- and terror-related posttraumatic stress disorder: a randomized control comparison with treatment as usual. *Journal of Clinical Psychiatry, 72*, 1174–1180.

Nasreddine, Z.S., Phillips, N.A., Bedirian, V., et al. (2005). The Montreal Cognitive Assessment, MoCA: A brief screening tool for mild cognitive impairment. *Journal of the American Geriatric Society, 53*, 695–699.

Pietrzak, R.H., Goldstein, R.B., Southwick, S.M., & Grant, B.F. (2011). Prevalence and axis I comorbidity of full and partial posttraumatic stress disorder in the United States: Results from Wave 2 of the National Epidemiologic Survey on Alcohol and Related Conditions. *Journal of Anxiety Disorders, 25*, 456-465.

Ramchand, R., Schell, T.L., Karney, B.R. et al. (2010). Disparate prevalence estimates of PTSD among service members who served in Iraq and Afghanistan: Possible explanations. *Journal of Traumatic Stress, 23*, 59-68.

Rauch, S.A.M, Defever, E., Favorite, T., et al. (2009). Prolonged Exposure for PTSD in a Veterans Health Administration PTSD clinic. *Journal of Traumatic Stress*, *22*, 60-64.

Resick, P.A, Galovski, T.E., Uhlmansiek, M.O., et al. (2008). A randomized clinical trial to dismantle components of cognitive processing therapy for posttraumatic stress disorder in female victims of interpersonal violence. *Journal of Consulting and Clinical Psychology, 76*, 243-258.

Resick, P.A., Monson, C.M. & Chard, K.M. (2010). *Cognitive processing therapy for PTSD: Veteran/Military version. Washington, DC: Department of Veterans Affairs*.

Resick, P.A., Nishith, P., Weaver, T.L., et al. (2002). A comparison of cognitive-processing therapy with prolonged exposure and a waiting condition for the treatment of chronic posttraumatic stress disorder in female rape victims. *Journal of Consulting and Clinical Psychology*, *70*, 867-879.

Resick, P.A., Williams, L.F., Suvak, M.K., et al. (2012). Long-term outcomes of cognitive-behavioral treatments for posttraumatic stress disorder among female rape survivors. *Journal of Consulting and Clinical Psychology, 80*, 201-210.

Rizvi, S.L., Vogt, D.S., & Resick, P.A. (2009). Cognitive and affective predictors of treatment outcome in Cognitive Processing Therapy and Prolonged Exposure for posttraumatic stress disorder. *Behavior Research and Therapy*, *47*, 737-743.

Rogers, E.M. (2003). *Diffusion of innovations*. 5th edition. New York: Free Press.

Rohde, P., Lewinsohn, P.M., & Seeley, J.R. (1997). Comparability of telephone and face-to-face interviews in assessing axis I and II disorders. *American Journal of Psychiatry,* 154, 1593-1598.

Rosetti, H.C., Lacritz, L.H., Munro Cullum, C., et al. (2011). Normative data for the Montreal Cognitive Assessment (MoCA) in a population-based sample. *Neurology*, 77, 1272-1275.

Rothbaum, B.O., Astin, M.C., & Marsteller, F. (2005). Prolonged Exposure versus Eye Movement Desensitization and Reprocessing (EMDR) for PTSD rape victims. *Journal of Traumatic Stress*, *18*, 607-616.

Sartor, C.E., Grant, J.D., Lynskey, M.T., et al. (2012). Common heritable contributions to low-risk trauma, high-risk trauma, posttraumatic stress disorder, and major depression. *Archives of General Psychiatry, 69*, 293-299.

Schnurr, P.P. (2007). The rocks and hard places in psychotherapy research. *Journal of Traumatic Stress*, *20*, 779-792.

Schnurr, P.P., Ford, J.D., Friedman, M.J., et al. (2000). Predictors and outcomes of PTSD in World War II veterans exposed to mustard gas. *Journal of Consulting and Clinical Psychology*, *68*, 258-268.

Schnurr, P.P., Friedman, M.J., Engel, C.C., et al. (2007). Cognitive-behavioral therapy for posttraumatic stress disorder in women: A randomized controlled trial. *Journal of the American Medical Association*, *297*, 820-830.
Schnurr, P.P., Friedman, M.J., Foy, D.W., et al. (2003). A randomized trial of trauma focus group therapy for posttraumatic stress disorder: Results from a Department of Veterans Affairs Cooperative Study*. Archives of General Psychiatry*, *60*, 481-489.
Schnurr, P.P., Friedman, M.J., Lavori, P.J., et al. (2001). Design of VA Cooperative Study #420: Group treatment of PTSD. *Controlled Clinical Trials*, *22*, 74-88.
Schnurr, P.P., Friedman, M.J., Oxman, T.E., et al. (2013). RESPECT-PTSD: Reengineering systems for the primary care treatment of PTSD, a randomized controlled trial. *Journal of General Internal Medicine, 28*, 32-40.
Schnurr, P.P., Lunney, C.A., Bovin, M.J., et al. (2009). Posttraumatic stress disorder and quality of life: Extension of findings to veterans of the wars in Iraq and Afghanistan. *Clinical Psychology Review*, *29*, 727-735.
Schnurr, P.P., Spiro, A. III, Vielhauer, M.J., et al. (2002). Trauma in the lives of older men: Findings from the Normative Aging Study. *Journal of Clinical Geropsychology*, *8*, 175-187.
Shalev, A.Y., Ankri, Y.L.E., Israeli-Shalev, Y., et al. (2012). Prevention of posttraumatic stress disorder by early treatment: Results from the Jerusalem Trauma Outreach and Prevention Study. *Archives of General Psychiatry, 69*, 166-176.
Spielberger, C.D. (1988). *State-Trait Anger Expression Inventory*. Odessa, FL: Psychological Assessment Resources.
Stanley, B., & Brown, G.K. (2008). *Safety plan treatment manual to reduce suicide risk: Veteran version*. Washington, DC: Department of Veterans Affairs.
Steenbarger, B.N. (1994). Duration and outcome in psychotherapy: An intergrative review. *Professional Psychology: Research and Practice, 25*, 111-119.
Stein, N. R., Mills, M. A., Arditte, K., et al. & the STRONG STAR Consortium. (2012). A scheme for categorizing traumatic military events. *Behavior Modification, 36*, 785-805.
Tanelian, T., & Jaycox, L.H. (Eds). (2008). *Invisible wounds of war: Psychological and cognitive injuries, their consequences, and services to assist recovery*. Santa Monica, CA: RAND.
VA Northeast Program Evaluation Center. (2012). *PTSD Fact Sheet, FY 2011*. West Haven, CT: Author.
van Minnen, A. (2012). Don't be afraid of the big bad wolf: Prolonged Exposure therapy for PTSD patients with severe comorbidity. Keynote address presented at the Dutch Cognitive Behavioral Therapy conference, Veldhoven, The Netherlands.
van Minnen, A., & Foa, E.B. (2006). The effect of imaginal exposure length on outcome of treatment for PTSD. *Journal of Traumatic Stress,* 19, 427-438.
van Minnen, A., Hendriks, L., & Olff, M. (2010). When do trauma experts choose exposure therapy for PTSD patients? A controlled study of therapist and patient factors. *Behaviour Research and Therapy, 48*, 312-320.
Veterans Benefits Association (2012). *Annual benefits report: Fiscal year 2011*. Washington, DC: Department of Veterans Affairs.
Vogt, D. (ongoing). "Gender, Stigma, and Other Barriers to VHA Use for OEF/OIF Veterans." D. Vogt, Principal Investigator. VA HSR&D Merit Review.
Wang, P.S., Berglund, P., Olfson, M., et al. (2005). Failure and delay in initial treatment contact after first onset of mental disorders I the National Comorbidity Survey Replication. *Archives of General Psychiatry, 62*, 603-613.
Watts, B.V., & Schnurr, P.P. (ongoing). "Decision Aid for Veterans with PTSD." V. Watts & P.P. Schnurr, co-Principal Investigators. VA HSR&D Merit Review.
Watts, B.V., Schnurr, P.P., Mayo, L., et al. (2012). *Meta-analysis of the efficacy of treatments for posttraumatic stress disorder*. Manuscript submitted for publication.

Weathers, F.W., Keane, T.M., & Davidson, J.R.T. (2001). The Clinician-Administered PTSD Scale: A review of the first ten years of research. *Depression and Anxiety*, *13*, 132-156.

Weathers, F.W., Litz, B.T., Herman, D.S., et al. (November, 1993). *PTSD Checklist: reliability, validity, and diagnostic utility*. Paper presented at the 9th Annual Meeting of the International Society for Traumatic Stress Studies, Chicago, IL.

World Health Organization (1996). *WHOQOL-BREF: Introduction, administration, coding, and generic version of the assessment.* Geneva, Switzerland: Author.

World Health Organization (2000). *WHODAS-II training manual: A guide to administration*. Geneva, Switzerland: Author.

Yoder, M., Tuerk, P.W., Price, M., et al. (2012). Prolonged Exposure therapy for combat-related posttraumatic stress disorder: Comparing outcomes for veterans of different wars. *Psychological Services, 9*, 16-25.

This page intentionally left blank.

| Department of Veterans Affairs | **RESEARCH CONSENT FORM**<br>***Version Date: October 13, 2017*** |
|---|---|

Participant Name: ______________________________ Date:__________

Title of Study: Comparative Effectiveness Research in Veterans with PTSD (CERV-PTSD)

Principal Investigator: ______________________________ VA Facility: __________________

Principal Investigator for Multisite Study: Paula P. Schnurr, PhD

## INTRODUCTION

You are being invited to take part in a research study that is funded by the Department of Veterans Affairs. Before you decide to take part, it is important for you to know why the research is being done and what it will involve. This includes any potential risks to you, as well as any potential benefits you might receive.

Read the information below carefully, and discuss it with family and friends if you wish. Ask one of the study staff if there is anything that is not clear or if you would like more details. Take your time to decide. If you do decide to take part, your signature on this consent form will show that you received all of the information below, and that you were able to discuss any questions and concerns you had with a member of the study team.

## BACKGROUND AND PURPOSE

### WHY IS THIS RESEARCH BEING DONE?

The purpose of this research study is to compare two types of individual therapies for the symptoms of Posttraumatic Stress Disorder (PTSD). One of the treatments is Prolonged Exposure (PE) and the other is Cognitive Processing Therapy (CPT). Both therapies are routinely used in the VA and have been found to be effective with Veterans in prior studies. However, the two therapies have never been compared to one another in Veterans.

PE involves learning a method of dealing with traumatic memories and stressful situations to help you overcome the distress in a safe manner. The other treatment, CPT, looks at the impact the traumatic event has had on your life and helps you to examine and change your unhelpful thoughts and feelings related to the event, yourself, others and the world. The purpose of this research study is to compare the effectiveness of these two therapies on PTSD symptoms, along with related symptoms such as depression and anxiety, to see which treatment is better. The study will also try to determine if there are people who respond better to one treatment or the other.

| Department of Veterans Affairs | **RESEARCH CONSENT FORM**<br>***Version Date: October 13, 2017*** |
|---|---|

Participant Name: ______________________________ Date:__________

Title of Study: Comparative Effectiveness Research in Veterans with PTSD (CERV-PTSD)

Principal Investigator: ______________________________ VA Facility: ________________

Principal Investigator for Multisite Study: Paula P. Schnurr, PhD

## WHY HAVE YOU BEEN ASKED TO TAKE PART IN THIS RESEARCH STUDY?

You are being asked to take part in this research study because you are over 18 and you may have PTSD. PTSD is a psychological disorder in some people who have had a trauma experience such as combat, sexual abuse, physical abuse, or natural disasters.

## WHO IS CONDUCTING THE RESEARCH STUDY?

This study is sponsored by the Department of Veterans Affairs. The study is directed by Paula P. Schnurr PhD, a researcher at the White River Junction VA Medical Center. Co-directors are Kathleen M. Chard, PhD at the Cincinnati VA Medical Center and Josef Ruzek, PhD at the Palo Alto VA. They are assisted by staff at the White River Junction VA Medical Center, the Palo Alto VA Cooperative Studies Program Coordinating Center (CSPCC), the Boston VA Medical Center, and your local VA hospital.

## HOW MANY PEOPLE WILL TAKE PART IN THE RESEARCH STUDY?

The CERV-PTSD study team at your medical center will ask Veterans like you to provide consent to participate in this research study. Study participation involves two parts. The first part is to go through screening procedures that will determine if you are eligible to receive PTSD therapy (PE or CPT) as part of the study. The second part of study participation is randomization to either PE or CPT treatment (“randomization” is described below in “Study Procedures”) and post-treatment follow-up. Not everybody who signs a consent form and goes through screening will qualify and receive PTSD therapy. We expect that approximately half of the participants will be eligible to receive study treatment. Up to 2550 Veterans at 17 or more VA sites across the country will be enrolled in this study, with up to 150 participants enrolled at each site.

## DURATION OF THE RESEARCH

The study will last four years, but you will be in the research study for approximately one year.

## STUDY PROCEDURES

### WHAT IS INVOLVED IN THE RESEARCH STUDY?

On your first visit you will be asked some questions to find out if you might be eligible for the study. The questions include background information and questions about your current mood and how you are coping. If it seems you are eligible for the study, you will fill out some additional paper-and-pencil questionnaires about PTSD, depression, anger, health and general well-being.

After this visit an assessor located in Boston or Long Beach will contact you by telephone for a clinical interview designed to determine if you have PTSD and other related symptoms. You also will be asked about your preferences for treatment, but this will not affect which treatment you receive. You do not need to return to the VA for this interview. However, if you do not have access to a phone you must agree to come to the local VA clinic for the phone interview. This phone call interview will take between two and four hours and can be done in two sessions. After this assessment it will be decided if you are eligible to participate in the study.

To be eligible for the study you must meet the following criteria:

- be enrolled in the VA system and referred to the study by a VA staff member,
- be a Veteran with a current diagnosis of PTSD due to any trauma during your military service,
- agree to be placed in either treatment (PE or CPT),
- agree to not receive other psychotherapy or counseling for PTSD while you are receiving therapy as part of this study,
- agree to let us access your medical record so we can learn about how much you are using VA services before and during the study,
- have regular access to a telephone or agree to come into the VA clinic for telephone interviews,

- agree to have your telephone interviews and treatment sessions recorded, and
- be at least eighteen years of age.

If more than 30 days go by between this telephone interview and your first study therapy session, you will be asked to re-do part of this interview. While receiving therapy as part of the study, you will be allowed to attend self-help groups, have brief check-in visits with any therapist or counselor you have now, seek treatment for substance abuse and mental health problems other than PTSD, and take medication for PTSD and other mental or physical conditions.

You will not be eligible for the study if you (1) have any current psychotic symptoms, (2) have plans to harm yourself or someone else or are making plans to do so, (3) have mania that is not in remission, (4) have current drug or alcohol dependence, or (5) show severe problems with memory or other problems with thinking and reasoning. If you are currently dependent on drugs or alcohol you will be referred to an appropriate clinic. You will be considered for the study one month after you are no longer dependent on drugs or alcohol. If you are currently suicidal or homicidal with intent and a plan we will help you obtain mental health care and you may be eligible for the study at a later time.

If you are eligible for the study you will be "randomized" into one of the two treatments described below. Randomization means that you are put into one treatment or the other completely by chance. It is like flipping a coin. If you take part in the study, you will be assigned to either PE or CPT with a trained therapist. You will have the same therapist for the entire study. Both therapies are commonly used in the clinical care of PTSD. They both involve 10-14 sessions that last 90 minutes in PE or 60 minutes in CPT. Sessions will be scheduled weekly, although you may attend some sessions more than once a week or skip a week (for a scheduling conflict, for example). You and your therapist will determine what frequency works best for you. Both therapies require practice assignments between sessions. At each session you will be asked to fill in two paper-and-pencil questionnaires.

The schedule for study assessments will be as follows:

| | Enrollment (On-site) | Baseline (Phone) | Therapy (8-14 weekly sessions) | Mid-treatment (Phone) | Post-treatment (Phone) | Post-Treatment (On-site) | 3 months post-treatment (Phone) | 3 months post-treatment (On-site) | 6 months post-treatment (Phone) | 6 months post-treatment (On-site) |
|---|---|---|---|---|---|---|---|---|---|---|
| PTSD symptom questionnaires | | X | X | X | X | | X | | X | |
| General mental health questionnaires | X | X | X | | | X | | X | | X |
| Life history questionnaires | X | X | | | | | | | | |
| Mood questionnaires | X | | | | | X | | | | X |
| End of study forms | | | | | | X | | | | X |

Halfway through your study treatment sessions, you will receive a telephone clinical interview that lasts about 1.5 hours. Within one week after the end of therapy you will be asked to come to the VA clinic for a post-treatment follow-up and have another telephone interview. You will also be asked to return for a follow-up assessment and complete phone interviews three and six months after you complete the treatment. The post-treatment and follow-up assessments will include all of the measures you filled in

on your first visit, with the exception of the general background questions. These visits will last one to two hours for the questionnaires in the clinic and about 1.5 hours for the telephone interviews. If you do not have access to a telephone we will ask you to come into the VA clinic for the telephone interview. This part of the study happens over the phone to make sure that the clinicians asking you certain types of questions are "blinded" – that is, that they do not know which treatment you were assigned to.

All treatment and questionnaire assessments will take place at your local VA. The interviews will be conducted on the telephone. All assessments will be digitally recorded and all therapy sessions will also be digitally recorded to ensure the quality of services being provided to you. You are free to skip any questions on any of the paper-and-pencil questionnaires that you would prefer not to answer. If you miss any of the treatment and questionnaire assessments the site coordinator will try to reschedule them by contacting you initially by phone (up to 5 times) and then by mail with a letter. If you are unable to attend a follow-up assessment at your local VA (for example, if you move out of the area), you may choose to complete some questionnaires by mail instead of in person). The site coordinator will contact you by phone prior to sending the study questionnaires by mail.

You will meet with several people as part of your research participation, including your study-assigned therapist, local site coordinator, and telephone assessor. Your local site coordinator will explain the potential risks and benefits of your participation. Study staff including the leaders of the project and your local site coordinator will monitor your treatment and whether undesirable events result from your participation. They will also alert you if there is a problem with the treatment. Your therapist will provide your PTSD therapy and also document your clinical course while you receive the treatment.
If you decide to participate in the research study, it will be your responsibility to:

- Attend scheduled treatment sessions
- Attend scheduled assessment appointments, and contact the site investigator or research staff to reschedule as soon as you know you will miss the appointment.

- Participate in the PTSD treatment process and complete treatment tasks as discussed with your therapist.
- Fill out your practice assignment forms as instructed.
- Complete your questionnaires as instructed.
- Ask questions as you think of them.
- Tell the site investigator or research staff if you change your mind about staying in the study.
- Not take part in any other research project without discussion with the research staff. Taking part in another research study without first discussing it with the investigators of this study may invalidate the results of both research studies..

**POSSIBLE RISKS OR DISCOMFORTS**

**WHAT ARE THE RISKS AND DISCOMFORTS OF THE RESEARCH STUDY?**

It is possible that during the assessments or therapy sessions you may feel some increase in unpleasant emotions while recalling and describing the traumatic event. Because you have PTSD you may have been trying to block or avoid thoughts and feelings. The goal of PE and CPT is to have you feel less stress and other painful emotions related to the traumatic event. We expect that any distress you may experience will be temporary. However it is possible that your condition may worsen. If at any time you are feeling overwhelmed or upset you may call the study staff between the hours of 8:00 AM and 4:00 PM, come to the main VA emergency room to be seen by a mental health professional, or call the Veterans Crisis Hotline at 1-800-273-8255.

If any significant new findings develop during the study that relate to your willingness to continue, you will be informed.

Risks of the usual care you receive (PE or CPT therapy for your PTSD) are not risks of the research. Those risks are not included in this consent form. You should talk with your health care providers if you have any questions about the risks of usual care.

## WHAT ARE THE RISKS OF STOPPING YOUR CURRENT TREATMENT?

The only risk to stopping your current therapy is the discomfort you may feel at changing from one therapist to another. You may have some discomfort with discontinuing your current treatment, but brief check-ins with your current therapist will be allowed.

## ARE THERE BENEFITS TO TAKING PART IN THE RESEARCH STUDY?

If you agree to take part in this research study, there may not be a direct benefit to you. The investigators hope the information learned from this research study will benefit you and other Veterans with PTSD in the future. Potential benefits to you may include a reduction in your PTSD symptoms over the course of therapy. The knowledge gained from this study will serve to guide future research and clinical care for Veterans. For society in general, this study will provide useful information regarding treatment effectiveness, recovery from trauma, and long-term benefits of therapeutic interventions.

## ALTERNATIVES TO PARTICIPATING IN THIS RESEARCH

## WHAT OTHER CHOICES FOR CARE ARE THERE?

PE and CPT are not investigational therapies. Both of these PTSD treatments have been found to be effective in past studies and they are available to you even if you decide not to participate in this study. This research study will compare the two treatments to one another.

Instead of being in this research study, you have these options:

If you have PTSD you may request PE, CPT, or other types of PTSD treatment based on VA guidelines and availability at your local VA or Vet Center. If you do not have PTSD you may contact the mental health clinic at your local VA or Vet Center to discuss your eligibility for other services.

**CONFIDENTIALITY**

**HOW WILL INFORMATION ABOUT YOU BE KEPT PRIVATE AND CONFIDENTIAL?**

The information collected for this study will be kept confidential. We will include information about your study participation in your medical record. There are times when we might have to show your records to other people. For example, someone from the Office of Human Research Protections, the Government Accountability Office, the Office of the Inspector General, the VA Office of Research Oversight, the VA Central IRB, our local Research and Development Committee, and other study monitors may look at or copy portions of records that identify you.

The data from the study may be published; however, you will not be identified by name. All data will be identified by code number. These data will be stored in locked file cabinets that will be accessible only to project staff.

The key listing names and code numbers will be kept in a separate locked filing cabinet or separate secure computer drive. Destruction of all research records pertaining to this study will be in accordance with the Department of Veterans Affairs record retention schedule. The electronic recordings of the assessments and sessions will be stored in the VA system with password protection.

Your information will be combined with information from other people taking part in the study. We will write about the combined information we have gathered. Any talks or papers from this study will not identify you.

If you are a VA patient, you already have a VA medical record. If you are not a current VA patient, we will create a VA medical record for you. Also, the VA Cooperative Studies Program requires us to collect Social Security Numbers (SSNs) from everyone who participates in this study in case there is new information about this study in the future that needs to be told to the participants. You will not be able to participate in this study unless you give us your SSN.

We will put the following information about you from this study into your medical record: A note that you are receiving one of the treatments and the session you are on, and the PTSD Checklist that you will fill out each session. This electronic record will be kept for 75 years after your last contact with us. All authorized users in the national VA system can have access to your medical record. We will also collect demographic information and VA services that you have received from your medical record.

By signing this document, you authorize the Veterans Health Administration (VHA) to permit (insert name of Site Investigator) and his or her research team to use and disclose the following information about you and to contact and discuss your research activities with your referring VA clinician to mutually address any clinical needs:

- Information about you that is created during the research study. This includes the number of times you have used VA services, the results of diagnostic exams that become part of the study records, and information collected as part of interviews you have with the study staff and questionnaires you fill out during the study.
- Information in your medical record that is needed for this research study. This might include the results of past physical exams, diagnostic interviews, lists of medications you are currently taking, diagnostic procedures and your medical, social, and psychiatric history.

## WHAT IS A CERTIFICATE OF CONFIDENTIALITY?

To further protect your privacy, the investigators have obtained a Certificate of Confidentiality from the Department of Health and Human Services (DHHS).

This helps protect your privacy by allowing us to refuse to release your name or other information outside of the research study, even by a court order. The Certificate of Confidentiality will not be used to prevent disclosures to local authorities of child abuse and neglect, elder abuse or neglect, or harm to self or others. The Certificate does not

prevent you or your family from releasing data about yourself or your involvement in this study.

A description of this clinical trial will be available on http://www.ClinicalTrials.gov as required by U.S. Law. This website will not include information that can identify you. At most, the website will include a summary of the results. You can search this website at any time.

**COSTS TO PARTICIPANTS AND PAYMENT**

**WHAT ARE YOUR COSTS TO BE IN THIS STUDY?**

There are no costs for your participation in the study. All study therapy is free of charge to study participants. Department of Veterans Affairs patients may be financially responsible for non-study related care at the Department of Veterans Affairs. Some Veterans are required to pay co-payments for medical care and services; these co-payment requirements will continue to apply to medical care and services provided by the Department of Veterans Affairs that are not part of this study.

**WILL YOU BE PAID TO PARTICIPATE IN THIS RESEARCH STUDY?**

You will be paid $30 for the screening. If it seems you are eligible for the study, you will be paid $20 for the baseline questionnaire measures before treatment and $50 for the initial telephone interview. You will receive $50 for the telephone interview during treatment and $75 for the phone interview and questionnaires at the end of treatment. At the three month follow-up, you will receive $85, and at the final assessment at 6 months, you will receive $100. Payments will be either in cash, gift card or check depending on the rules for each VA hospital in the study. If you receive payments for being a part of this research study, you may be asked to complete an Internal Revenue Service (IRS) form. The amount you receive may count as income and may affect your income taxes. Your social security number may be required to complete the IRS 1099 form. You will also be reimbursed for travel over 50 miles.

## MEDICAL TREATMENT AND COMPENSATION FOR INJURY

**WHAT COMPENSATION IS AVAILABLE IN CASE OF INJURY?**

Every reasonable safety measure will be used to protect your well-being. If you are injured as a result of taking part in this study, the VA will provide necessary medical treatment at no cost to you unless the injury was due to your not following the study procedures. Financial compensation is not available for such things as lost wages, disability or discomfort due to an injury. The Department of Veterans Affairs does not normally provide any other form of compensation for injury. You have not released this institution from liability for negligence.

If you should have a medical concern or get hurt or sick as a result of taking part in this study, call: *(List local site contacts)*

DURING THE DAY:

Dr./Mr./Ms. __________________________ at ________________________ and

AFTER HOURS:

Dr. /Mr./Ms.__________________________ at ________________________.

Emergency and ongoing medical treatment will be provided as needed.

You do not give up any of your legal rights and you do not release the VA from any liability by signing this form.

## PARTICIPATION IS VOLUNTARY

It is up to you to decide whether or not to take part in this study. If you decide to take part you may still withdraw at any time. If you do not wish to be in this study or decide to leave the study early, you will not lose any benefits to which you are entitled. If you

do not take part, you can still receive all usual care that is available to you. Your decision not to take part will not affect the relationship you have with your doctor or other staff, and it will not affect the usual care that you receive as a patient.

If you decide to withdraw from therapy you will be asked to complete remaining assessments, but again, this is voluntary and you will not be penalized for declining. If you withdraw from the study, data that has already been collected as part of the study can be utilized by the study team, but no future data will be collected without your permission.

**RIGHT OF INVESTIGATOR TO TERMINATE PARTICIPATION**

The investigative team may terminate your participation in the study if they believe it is in your best interest or if you are not following study requirements for treatment or assessments. If so, your therapist will explain the reasons and arrange for your usual medical care to continue. Termination from the study will not affect the relationship you have with your doctor or other staff, and it will not affect the usual care that you receive as a patient.

**PERSONS TO CONTACT ABOUT THIS STUDY**

**WHO DO YOU CALL IF YOU HAVE QUESTIONS OR PROBLEMS?**

If you have any questions regarding this study, if you experience side effects or want to report a research-related injury or illness, or if you have any additional concerns or complaints while you are participating in this study, you can contact the site investigator [insert SI name here] at [(xxx) xxx-xxxx].

If you have questions about your rights as a study participant, or you want to make sure this is a valid VA study, you may contact the VA Central Institutional Review Board (IRB). This is the Board that is responsible for overseeing the safety of participants in this study. You may call the VA Central IRB toll free at 1-877-254-3130 if you have

questions, complaints or concerns about the study or if you would like to obtain information or offer input.

To report complaints or concerns to an independent agency in an anonymous and confidential manner, please call the Research Compliance Hotline at 1-800-889-1547.

**SIGNIFICANT NEW FINDINGS**

Sometimes during the course of a research study, new information becomes available about the therapies being studied that might change a person's decision to stay in the study. If this happens, your therapist will tell you about it and discuss with you whether you want to continue in the study. If you decide to withdraw from the study, your therapist will arrange for your mental health care to continue. If you decide to continue in the study, you might be asked to sign an updated informed consent form.

**AGREEMENT TO PARTICIPATE IN THE RESEARCH STUDY**

[insert SI name here] or a member of his/her research team has explained the research study to you. You have been told of the risks or discomforts and possible benefits of the study. You have been told of other choices of treatment available to you. You have been given the chance to ask questions and obtain answers.

You voluntarily consent to participate in this study. You also confirm that you have read this consent, or it has been read to you. You will receive a copy of this consent after you sign it.

**I agree to participate in this research study as has been explained in this document.**

| Department of Veterans Affairs | **RESEARCH CONSENT FORM**<br>***Version Date: October 13, 2017*** |
|---|---|

Participant Name: ______________________________ Date:__________

Title of Study: Comparative Effectiveness Research in Veterans with PTSD (CERV-PTSD)

Principal Investigator: ______________________ VA Facility: ______________

Principal Investigator for Multisite Study: Paula P. Schnurr, PhD

| | | |
|---|---|---|
| ______________________<br>Participant's Name | ______________________<br>Participant's Signature | __________<br>Date |
| ______________________<br>Name of person obtaining authorization and consent | ______________________<br>Signature of person obtaining authorization and consent | __________<br>Date |

2921

SUBJECT'S IDENTIFICATION

VA Form **10-10-86**
MAR 2006

**VA CENTRAL IRB APPROVAL STAMP**

*VA Central IRB Template October 5, 2011*

## Appendix B. BIOSTATISTICAL AND RESEARCH DATA PROCESSING (BRDP)

### A. Data Management

See **Section IX** of the protocol for a description of the procedures for data collection, management, and security for the study.

### B. Statistical Reports

The figures and tables in this section are examples of the type of information that will be generated during the study for periodic evaluation by the Executive Committee and the Data Monitoring Committee. They are listed as follows:

- Figure B.1: Screened vs. Randomized by Hospital
- Figure B.2: Participant Intake Graph
- Table B.1: Recruitment by Hospital
- Table B.2: Balance on Major Subgroup Variables
- Table B.3: Randomizations over Time
- Table B.4: Number Failed Inclusion/Exclusion Criteria
- Table B.5: Baseline Characteristics
- Table B.6: Assessment Summaries at Baseline (and Follow-up)
- Table B.7: Psychotropic Medications at Baseline (and Follow-up)
- Table B.8: Termination from Study
- Table B.9: SAE from Study
- Table B.10: Number of Forms Received/Missing
- Table B.11: Number of Forms Received with Errors

Tables on participant accrual will be used to monitor the progress of participant enrollment into the study, overall and by participating hospital. Baseline characteristics will be compared by hospital to ensure the comparability of participants across hospital. Terminations, SAE and counts of data forms missing or with errors will also be compared by hospital. The data in the figures and tables will be supplied to the DMC by treatment group as well as overall.

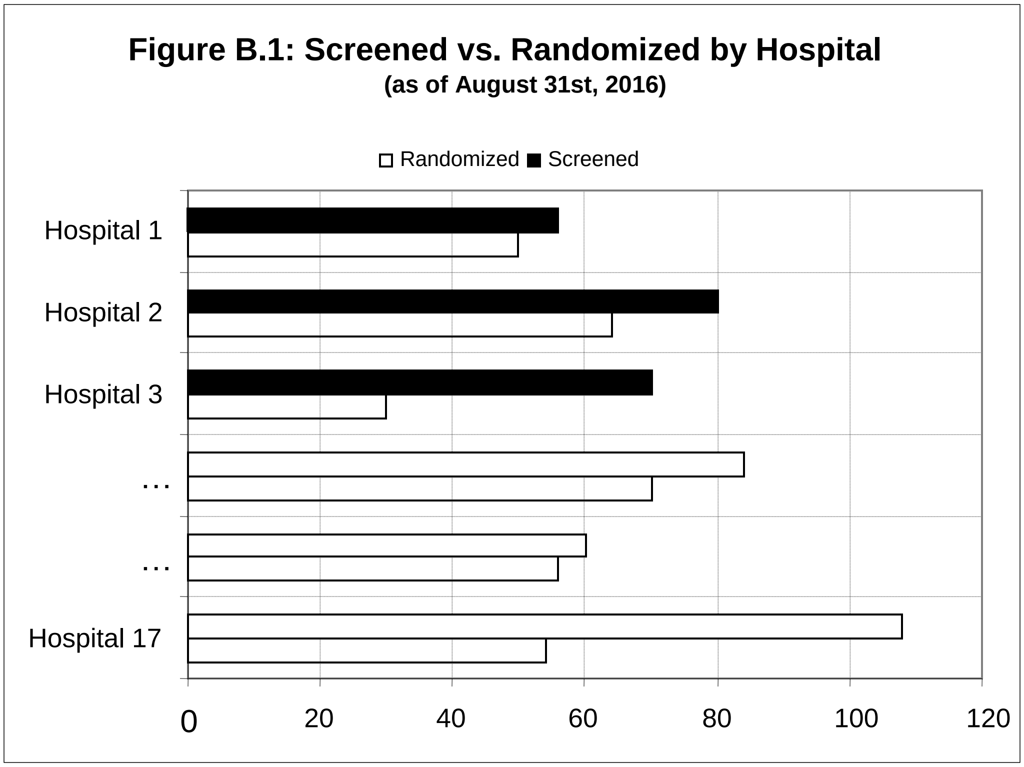


**Figure B.1: Screened vs. Randomized by Hospital**
**(as of August 31st, 2016)**

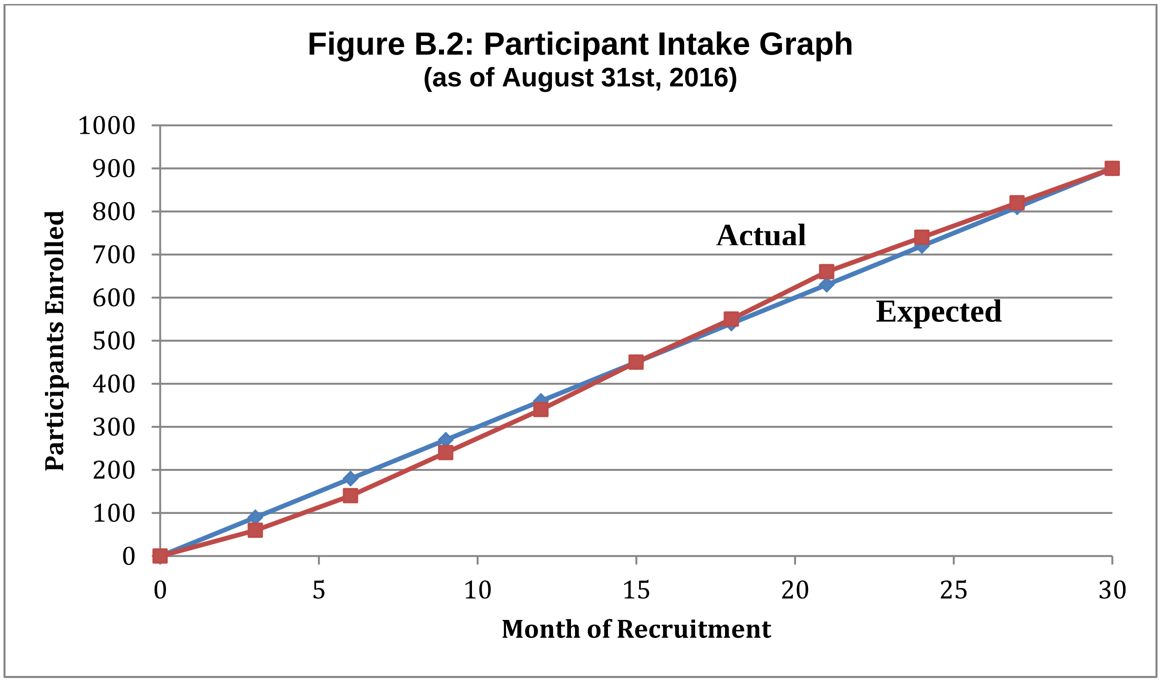


**Figure B.2: Participant Intake Graph**
**(as of August 31st, 2016)**

**Table B.1: Recruitment by Hospital**

| | No. Screened | No. Randomized (%) |
|---|---|---|
| Hospital:<br>- Hospital 1<br>- Hospital 2<br>- …<br>- Hospital 17 | | |
| Total | | |

**Table B.2: Balance on Major Subgroup Variables**

| | Treatment Group | | |
|---|---|---|---|
| | Treatment A | Treatment B | Total |
| Hospital | | | |
| - Hospital 1<br>- Hospital 2<br>- …<br>- Hospital 17 | | | |
| Gender<br>- Male<br>- Female | | | |
| OEF/OIF/OND<br>- Y<br>- N | | | |
| Race<br>- White<br>- Black<br>- Others | | | |
| Total | | | |

**Table B.3: Randomizations over Time**

| Study Month | Hosp1 Hosp2 Hosp3 .... Hosp17 | Total |
|---|---|---|
| 1 | | |
| 2 | | |
| 3. | | |
| ... | | |
| 30 | | |
| Total | | |

**Table B.4: Number Failed Inclusion/Exclusion Criteria**

| | Hosp1 Hosp2 .... Hosp17<br>(N and %) | Total<br>N % |
|---|---|---|
| Number Screened | | |
| Failed Inclusion Criteria:<br>- Current conformed diagnosis of PTSD<br>- One or more military trauma event<br>- Agrees not to receive other psychotherapy for PTSD during study treatment<br>- Stable psychoactive medication 30 days prior to entering the study<br>- CAPS total score greater than or equal to 45<br>- … | | |
| Failed Exclusion Criteria:<br>- Substance dependence not in remission for at least one month<br>- Current psychotic symptoms<br>- Current mania or manic phase of bipolar disorder<br>- Significant current suicidal or homicidal ideation<br>- Moderate to severe cognitive impairment<br>- … | | |
| Did not sign consent form | | |
| Number Randomized | | |

**Table B.5: Baseline Characteristics**

| Characteristic | Hosp1 Hosp2 .... Hosp17<br>(N and %, or mean and SD) | Total<br>N % |
|---|---|---|
| Age | | |
| Work Status<br>- Employed full-time<br>- Employed part-time<br>- … | | |
| VA Service Disability<br>- Applied, but denied<br>- Approved (nonzero%)<br>o Percentage | | |
| PTSD Service Disability<br>- Applied, but denied<br>- Approved (nonzero%)<br>o Percentage | | |
| Medical/Psychiatric History: | | |

| | | |
|---|---|---|
| - … | | |

**Table B.6: Assessment Summaries at Baseline (and Follow-up)**

| | Hosp1 Hosp2 .... Hosp17<br>(mean and SD) | Overall<br>Mean SD |
|---|---|---|
| Primary Assessments:<br>- CAPS<br>o Reexperiencing Symp.<br>o Avoidance Symp<br>o Numbing Symp.<br>o Hyperarousal Symp. | | |
| Secondary Assessments:<br>- PDS<br>- BDI-II<br>- STAXI<br>- WHO-DAS-II<br>- WHOQOL-BREF<br>- … | | |

**Table B.7: Psychotropic Medications at Baseline (and Follow-up)**

| Medication | Hosp1 Hosp2 .... Hosp17<br>(N and %) | Total<br>N % |
|---|---|---|
| | | |
| | | |
| | | |
| | | |
| | | |
| | | |
| | | |
| … | | |
| Total | | |

**Table B.8: Termination from Study**

| | Hosp1 Hosp2 .... Hosp17 | Total |
|---|---|---|
| Reason for Termination:<br>- Participant withdrew (related to treatment)<br>- Participant withdrew (unrelated to treatment)<br>- Moved away<br>- Lost to follow-up<br>- Died | | |
| Withdrew HIPAA Authorization:<br>- Y<br>- N | | |
| Total | | |

**Table B.9: SAE from Study**

| | Hosp1 Hosp2 .... Hosp17<br>(N and %) | Total<br>N % |
|---|---|---|
| Adverse Event:<br>- Event1<br>- Event2<br>- … | | |
| Total | | |

**Table B.10: Number of Forms Received/Missing**

| Form | Hosp1 Hosp2 ....<br>rec'd missing rec'd missing | Total<br>rec’d missing |
|---|---|---|
| 01 | | |
| 02 | | |
| ... | | |
| xx | | |
| Total | | |

**Table B.11: Number of Forms Received with Errors**

| Forms | Hosp1 Hosp2 .... Hosp17 | Total |
|---|---|---|
| Number submitted | | |
| Number with missing data | | |
| Number with data out of range | | |
| Number randomized by mistake | | |
| Number of invalid codes | | |

**Appendix C. RESEARCH DATA FORMS**

The sample forms that are used to collect the assessments in **Table VIII.1** are listed as follows:

- Clinician-Administered PTSD Scale
- Posttraumatic Diagnostic Scale
- Patient Health Questionnaire-9
- Spielberger State Anger Inventory
- Brief Addiction Monitor (2 items)
- Short Inventory of Problems-Revised
- WHO-DAS-II
- WHOQOL-BREF
- Client Satisfaction Questionnaire
- Treatment preference
- Expectancy of Therapeutic Outcome
- Service Utilization
- Structured Clinical Interview for DSM-5
- MoCA
- Demographic information
- VA TBI Screen
- VA MST Screen
- Life Events Checklist
- PTSD Checklist
- Beck Depression Inventory-II
- Blinded Consult Request Form

## Appendix D. OWNERSHIP, CONTROL AND ACCESS TO STUDY DATA

### A. Ownership and Control

The CSP will retain all ownership rights to the study data, including original data and any derived data generated from the original data. If the CSP study is being conducted with a clinical research agreement with another institution, the terms of that agreement will define any alternate conditions for ownership and control of study data. All study data will reside at the CSPCC/ERIC or on its designated VA Research and Development servers and will not be released until the objectives as stated in the protocol and the primary manuscript(s) identified in the protocol publication plan have been completed. The CSPCC/ERIC will act as the repository of all study data from a completed cooperative study.

The study Executive Committee has the authority to determine all uses of the data, provided that these uses do not conflict with the study protocol, CSP policies, VA policy or other regulations. Potential uses include analyses of the data, publication of the results of analyses, or distribution of copies of all or part of the study dataset.

VA CSP is authorized to share the data for the purposes indicated under the Health Insurance Portability and Accountability Act (HIPAA) Privacy Rule, 45 CFR 164.512(i) and authorities stated in VHA Handbook 1200.12. Data may be released to other investigators after the planned objectives and primary manuscript(s) are completed and upon approval of the Study Chairman, Executive Committee (if it still exists), CSPCC/ERIC Director and Director, CSR&D (see next section). Data use agreements, including assurance that all VA data security policies will be strictly adhered to, will be instituted prior to any data being released.

### B. Release and Sharing of Study Data Sets

While the CSP is the custodian of study data, the CSP does not seek to limit the use of the data, but rather to ensure that these data sets are being appropriately used scientifically and ethically and that the rights and welfare of study participants are protected. Local Site investigators (LSIs) are encouraged to submit proposals to the Executive Committee for use of the data and these will be approved if scientifically and ethically sound. Data sets will not be released before the study database is locked and until the objectives as stated in the protocol and the primary manuscript(s) have been completed.

The Director, CSPCC/ERIC will have the authority to release data sets to Local Site Investigators/Executive Committee members, who have been given approval for access to

these data sets by the Executive Committee or by the Study Chairs if the Executive Committee is no longer functioning. Investigators outside of the study, both VA and non-VA, must obtain approval for release of data by the Executive Committee (if still functioning), the Director, CSPCC/ERIC, and the Director, CSR&D. All recipients of CSP databases must sign a data use agreement that stipulates that the recipient:

- will only use the data for the purposes stated in the data use agreement,
- will give proper credit to the study and the CSP and VA in all presentations and publications,
- will not give this data to other investigators without consent of the Directors, CSPCC/ERIC and CSR&D,
- will destroy or return the data when they have completed their work,
- will not try to identify any study participant, and
- will consider the data sets as confidential information and will keep the data sets in a secure location.

In addition, sharing of the data with another facility or institution will require evidence of approval and any appropriate waivers by the IRB and/or Research Committee and Information Security Officer of that institution. Sharing of CSP Study data outside of the CSP Study may be limited by specifications in the language of the study informed consent or HIPAA privacy authorization form.

The CSPCC/ERIC, for its part, will provide the investigator requesting the data with de-identified datasets or aggregate data, making identification of study participants as difficult as possible. HIPAA guidelines for de-identified data sets will be used, whenever possible. Investigators will not be provided with full study databases, but rather just with the information that they will need to do their research. The CSPCC/ERIC's main responsibility is to prepare the needed analyses for the primary manuscript(s) and secondary manuscripts identified in the protocol or planned by the Executive Committee. Secondary analyses by the CSPCC/ERIC may be delayed until the primary analyses and manuscripts are completed. Preparation of datasets and developing data use agreements for sharing data will have a lower priority than the completion of study analyses planned by the Executive Committee. Alternatively, the CSPCC/ERIC may provide the LSIs with appropriate data sets if they have the resources to use these data sets.

**Appendix E. UPDATES TO THE PROTOCOL**

On occasion, changes will be made to the study protocol. It is vital that these changes are reflected in your copy of the protocol. The following serves as a permanent record of all changes and additions (other than minor editorial changes) that have occurred since initial printing.

| **Version number and date** | **Section(s)** | **Page numbers in version where changes were implemented** | **Description** |
|---|---|---|---|
| Version 11.0. February 16, 2018 | All | All | Added new version number and version date to footer throughout the protocol. |
| | VIII.G | 51 | The Fidelity Monitoring process has been updated. |
| Version 10.0. October 18, 2017 | All | All | Added new version number and version date to footer throughout the protocol. |
| | VII.C | 36-37 | Added a VFF Follow-up form. |
| | VIII.A.1 | 39 | Table VIII.1 was updated to include the VFF Follow-up form. |
| | VIII.B.2 | 43 | Language was added to clarify that therapy sessions can occur more or less frequently than once per week. |
| | VIII.D | 50 | Language was added to allow for re-consent if a participant withdraws from the study (or is declared lost to follow-up). |
| | Appendix A | All | Consent form template was updated to reflect the most current version. |
| Version 9.0. August 1, 2017 | All | All | Added new version number and version date to footer throughout the protocol. |
| | IV.A | 19 | Added information regarding excluding patients who are currently incarcerated. |
| | IV.C.3 | 26 | Added language regarding the requirement to re-administer the CAPS if more than 30 days elapses between the Phase 3 appointment and the first treatment session. |

| Version number and date | Section(s) | Page numbers in version where changes were implemented | Description |
|---|---|---|---|
| | Appendix A | All | Consent form template was updated to reflect the most current version |
| Version 8.0. August 23, 2016 | All | All | Added new version number and version date to footer throughout the protocol. |
| | X.C | 59 | Added language to clarify that the study may enroll (i.e. consent) up to 2550 participants in order to reach the randomization goal of 900 participants. |
| Version 7.0, May 23, 2016 | All | All | Added new version number and version date to footer throughout the protocol. |
| | IV.B | 19, 20 | Added information about the Women's Enhanced Recruitment Process (WERP). |
| | IV.B | 21-23 | Added the following sub-categories to the Recruitment section: "SC-initiated direct contacts to women Veterans who appear to be potentially eligible for the study", "SC-initiated direct contacts to all women patients at a facility", and "Women's Enhanced Recruitment Process (WERP)". |
| | IV.C | 23 | Added language that will allow primary care providers to refer to the study. The sentence now reads "All participants, including self-referrals, will enter the study through referral by a mental health clinician or other qualified clinician at the participating site". |
| | VII.C | 36-37 | Added information about the Veteran Feedback Form (VFF). |
| | VIII.A.1 | 39 | Added the Veteran Feedback Form to the Assessment Schedule (Table VIII.1). |
| | IX.A | 52, 53 | Added information regarding the data management of the Veteran Feedback Form. |

| Version number and date | Section(s) | Page numbers in version where changes were implemented | Description |
|---|---|---|---|
| | XIII | 73 | Added language to indicate that the PBRN Coordinating Center team members will assist with monitoring recruitment and retention of women, and will conduct data entry of VFF data, and analyses of VFF findings. |
| Version 6.0, October 23, 2015 | All | All | Added new version number and version date to footer throughout the protocol. |
| | VIII.A.4 | 38 | Added language to allow on-site follow-up assessments to be completed via mail when a participant is unable to complete these assessments in-person (e.g., if the participant moved away after completion of treatment). |
| | XI.B,C, E | 63, 66, 67 | The language in this section was updated to reflect the current procedures for monitoring and reporting Adverse Events. |
| | Appendix A | All | Consent form template was updated to allow on-site follow-up assessments to be completed via mail when a participant is unable to complete these assessments in-person. |
| | Appendix B | B.5 | Table B.7 was edited to remove the names of medications (to prevent any confusion that this is meant to be a complete list of psychotropic medications that are monitored for the study). |
| Version 5.0, August 20, 2015 | All | All | Added new version number and version date to footer throughout the protocol. |
| | IV.A | 19 | The medication stabilization criteria was changed from 2 months prior to study entry to 30 days prior to study entry. |
| | VIII.C | 46 | Changed the name of the PE Master Therapist from Peter Tuerk to Edna Foa. |
| | Appendix A | All | Consent form template was updated. |

| Version number and date | Section(s) | Page numbers in version where changes were implemented | Description |
|---|---|---|---|
| Version 4.0, March 25, 2015 | All | All | Added new version number and version date to footer throughout the protocol. |
| | I.E | 12 | Clarified that the exclusion is for psychotic symptoms or mania (including manic phase of bipolar disorder). |
| | III | 17 | The footnote for Table II.1 was updated to state that “Enrollment typically will take about 1 month”. |
| | IV.A | 19 | Clarified that the exclusion is for psychotic symptoms or mania (including manic phase of bipolar disorder). |
| | VIII.B.5 | 43-44 | Updated the language to indicate that the study Suicide Assessment Procedures should be followed if risk is indicated by the participant’s responses to Item 1.9 of the PHQ-9 or by study therapist observations during the course of normal interactions with the participant. The previous language had incorrect guidance about what should be reported as an AE. |
| | Appendix A | All | Consent form template was updated (the previous language suggested that bipolar disorder is an exclusion criterion, but that was never the intention) |
| Version 3.0, February 11, 2015 | All | All | Added new version number and version date to footer throughout the protocol. |
| | IV.A | 19 | The requirement to sign the VA Form 10 3203 (Consent for Use of Picture and/or Voice) has been removed. Per the new VHA Handbook 1200.05, study participants are no longer required to sign a separate audio consent form if the appropriate language is included in the study consent form. |
| | V.A | 25 | Removed language about participants signing a VA Form 10 3203 to record therapy sessions for imaginal exposure homework exercises for PE. |

| Version number and date | Section(s) | Page numbers in version where changes were implemented | Description |
| --- | --- | --- | --- |
| | X.B.3 and X.C | 54-55 | Changed language to specify that we expect 900 participants to be *randomized* into the study, and around 64 participants to be *randomized* at each participating study site (the previous language was not in accordance with CIRB's definition of "enrolled", which includes screen failures after consent). |
| | Appendix A | All | Consent form template was updated. |
| Version 2.0, October 24, 2014 | All | All | Added new version number and version date to footer throughout the protocol. |
| | IV.A | 19 | Added language to clarify that only psychoactive medication must be on a stable regimen for 2 months prior to study entry, and defined "stable regimen" to be no dose or medication change. |
| | VIII.A.2 | 37 | Language referencing the specific Konexx USB Phone 2 PC program used to record telephone assessments has been deleted. |
| | XI.C.1 | 64 | The reporting period for AEs was corrected. |
| | Appendix A | All | Consent form template was updated. |
| Version 1.5, March 6, 2014 | All | All | Added new version number and version date to footer throughout the protocol. |
| | Appendix A | All | Draft consent form template was updated. |
| Version 1.4, November 18, 2013 | All | All | Added new version number and version date to footer throughout the protocol. |
| | IV.A | 19 | Updated the inclusion criteria to include the completion of the VA Form 10-3203. |
| | V.A | 25 | Included an explanation that participants will bring home recordings of their session per the usual manualized PE treatment protocol and that this is not behind the VA Firewall. |

| **Version number and date** | **Section(s)** | **Page numbers in version where changes were implemented** | **Description** |
|---|---|---|---|
| | Appendix A | All | Draft consent form template was updated. |
| Version 1.3, October 22, 2013 | All | All | Added new version number and version date to footer throughout the protocol. |
| | IV.A | 19 | Substance abuse was added to the inclusion criteria. |
| | IV.C.1 | 22 | Language was added to clarify that the SC will contact the potential participants initially by phone (up to 5 times) and then by mail with a letter. |
| | IV.C.3 | 23 | Language was added to clarify the procedures for contacting participants to provide them with the details of their treatment assignments and to schedule their initial therapy appointment. |
| | VIII.A.2 | 36 | Language was added to clarify that the independent assessors are members of the research team and are VA or WOC employees. |
| | VIII.A.6 | 39 | Added language to indicate that participants will be reimbursed for travel over 50 miles. |
| | VIII.F | 47 | “Supervisors” was changed to “Therapy Supervisors” and language was added to clarify their position and duties. |
| | VIII.G | 48 | Information was added about digital recordings. |
| | IX.B | 51 | Update was made to reflect that records will be maintained and destroyed per the VHA Records Control Schedule (RCS-10-1). |
| | XI.B | 63 | The publication date of Handbook 1058.01 was updated to the most current version date of November 2011. |
| | XI.E | 67 | Added language that CIRB procedures and VHA Handbook 1058.01 will be followed for reporting AEs. |
| | XII.D.1 | 69 | Language was added to indicate that CIRB will be informed of all site terminations and probations. |

| Version number and date | Section(s) | Page numbers in version where changes were implemented | Description |
| --- | --- | --- | --- |
| | XII.D.2 | 69 | Updates were made to indicate that CIRB will be the IRB of record for all study sites and that protocol violations must be reported to CIRB. |
| | XIII | 70 | Mention of the Human Studies Subcommittee was removed and replaced with Central IRB. |
| | Appendix A | All | Draft consent form template was updated. |
| | Appendix C | C-1 | Blinded Consult Request Form was added to the list of Research Data Forms. |
| Version 1.2, October 9, 2013 | All | All | Added new version number and version date to footer throughout the protocol. |
| | Appendix A | All | Draft consent form template was updated. |
| Version 1.1, September 4, 2013 | All | All | Added study number, version number, and version date to footer throughout the protocol. |
| | IV.B | 19-21 | More details about study recruitment were added. |
| | IV.C | 21 - 23 | More details about screening and consent were added |
| | VII.C | 33-34 | Language was updated to reflect the rollouts current usage of PHQ-9 to monitor depression symptoms in PE and CPT (rather than BDI-II). |
| | VIII.A.1 | 35-36 | Table VIII.1 (Assessment Schedule) was updated, switching the BDI-II and PHQ-9 measures as a result of the change in the rollout practice. |
| | VIII.A.2 | 36-37 | More details were added about the telephone assessment |
| | VIII.A.6 | 39 | Justification for the payment schedule was added. |
| | VIII.B.3 | 42 | Language was added to indicate that if participants do not complete treatment within 20 weeks, they will be offered 1-2 additional sessions, including the termination session of their assigned treatment. |

| **Version number and date** | **Section(s)** | **Page numbers in version where changes were implemented** | **Description** |
|---|---|---|---|
| | IX.A | 49 | Updates were made regarding the data forms. |
| | X.D.3 | 59 | Table X.3 (Secondary Outcomes) was updated, switching the BDI-II and PHQ-9 measures as a result of the change in the rollout practice. |
| | Appendix A | All | Draft consent form template was updated. |
| | Appendix E | All | Added Appendix E to track updates to the protocol. |
| Version 1.0, June 12, 2013 | NA | NA | Approved by CSP Central Office on 8/27/2013 |
